# Extending single-cell proteomics from HeLa to small primary human immune cells

**DOI:** 10.1038/s41467-026-77675-x

**Published:** 2026-09-19

**Authors:** Ivo A. Hendriks, Sara C. Buch-Larsen, Martin Rykær, Maico Y. Lechner, Anders H. Kverneland, Tabiwang N. Arrey, Daniel Hermanson, Eugen Damoc, Jesper V. Olsen

**Affiliations:** 1 Proteomics program, Novo Nordisk Foundation Center for Protein Research, Department of Cellular and Molecular Medicine, Faculty of Health and Medical Sciences, University of Copenhagen, Copenhagen, Denmark; 2 Copenhagen Center for Glycocalyx Research, Department of Cellular and Molecular Medicine, Faculty of Health and Medical Sciences, University of Copenhagen, Copenhagen, Denmark; 3 National Center for Cancer Immune Therapy, Department of Oncology, Copenhagen University Hospital, Herlev, Denmark; 4 Thermo Fisher Scientific, 11 Hanna-Kunath Strasse, Bremen, Germany

**Keywords:** Proteomic analysis, Proteomics, Mass spectrometry

## Abstract

Single-cell proteomics (SCP) holds the promise of decoding cellular heterogeneity at the functional level, yet achieving deep and reproducible proteome coverage from individual cells has remained a formidable challenge. Here, we establish a high-sensitivity, label-free SCP platform that extends current depth and reproducibility. By integrating optimized low-input sample processing, refined liquid chromatography, and the Orbitrap Astral Zoom mass spectrometer, our approach routinely quantifies up to 7000 proteins per individual HeLa cell at over 100 samples-per-day (SPD), capturing thousands of low-abundance proteins that eluded prior SCP studies. Applied to very small human peripheral blood mononuclear cells (PBMCs), we identify up to 4000 proteins per cell, including key markers that distinguish monocytes, T cells, and activated lymphocytes within heterogeneous populations, underscoring that single-cell proteomics can now directly elucidate clinically relevant primary samples with both depth and precision.

## Abstract (web summary)

Single-cell proteomics can reveal cellular heterogeneity but remains challenging for small cells. Here, the authors establish a sensitive label-free workflow that quantifies up to 7,000 proteins per HeLa cell and up to 4,000 proteins in small human immune cells.

## Introduction

The recent technological advances made in single-cell proteomics (SCP) have revolutionized our ability to interrogate the proteome at the resolution of individual cells, offering unprecedented insights into cellular heterogeneity, functional diversity, and the dynamics of biological processes that are often masked by dilution in bulk analyses. Unlike traditional bulk proteomics, which averages protein abundances across large populations of cells, SCP enables the direct quantification and characterization of proteins, including their post-translational modifications, within single cells. This granular approach is critical for understanding complex biological phenomena such as cellular differentiation, disease progression, immune responses, and drug resistance, as well as for identifying rare cell types that may play pivotal roles in health and disease.

The last few years have witnessed remarkable progress in both the sensitivity and throughput of SCP, driven by innovations in sample preparation workflows and liquid chromatography-mass spectrometry (LC-MS) acquisition strategies^1^. The primary challenge in SCP lies in the minute amount of protein available from individual cells, necessitating highly efficient and low-loss sample processing workflows coupled with ultra-sensitive MS detection. The SCP field has seen a surge in the development of miniaturized and automated sample preparation platforms. This includes the nanoPOTS (Nanodroplet Processing in One pot for Trace Samples) and the more recent nested NanoPOTS (N2) chip, which leverage microfluidic and nanodroplet technologies to minimize sample loss, reduce reaction volumes, and enable the parallel processing of hundreds of single cells^2^. These platforms have demonstrated robust quantification of thousands of proteins per cell, with the latest iterations, such as the proteoCHIP EVO 96 and the Chip-Tip workflow, pushing the boundaries further by enabling the identification of >5000 proteins from individual cells in a high-throughput and nearly loss-less manner^3^. Parallel to advances in sample preparation, highly sensitive and fast sequencing mass spectrometers have been introduced to meet the demands of SCP^3–5^. Data-independent acquisition (DIA) has emerged as a preferred MS acquisition strategy due to its reproducibility, comprehensive coverage, and suitability for low-input samples. Optimized narrow-window DIA workflows^6^ often coupled with short LC nanoflow gradients and spectral library-free data analysis, have enabled the identification of thousands of proteins from single cells with high reproducibility and quantitative accuracy. Additionally, isobaric labeling techniques, such as those employed in SCoPE-MS^7^, utilizing tandem mass tags and carrier channels to boost signal intensity and facilitate multiplexed analysis, further enhancing throughput and sensitivity^8^.

Despite these advances, SCP still faces challenges related to sample loss, data analysis, and the need for further improvements in sensitivity and throughput. Ongoing efforts in miniaturization, automation, and the development of robust computational tools are essential for overcoming these limitations and realizing the full potential of SCP in both basic and translational research. Using a pulsed-SILAC strategy, we have recently demonstrated that SCP can be used to study the relationship between the turnover and concentrations of certain proteins compared to cell size in human cells^9^.

Protein content in most cell types across the tree of life is in the range of 15–35% of the total cell volume, with cellular protein concentration typically ~200–300 mg/mL. This cellular constraint is thought to be a result of evolutionary pressure to optimize metabolic efficiency^10^. A single cell’s total protein mass can therefore vary significantly with cell size, with larger cells having more protein copies. However, not all proteins scale equally; while some, like histones, are diluted, others are concentrated as cells grow. Cell size is a major source of proteome heterogeneity, and consequently cell size effects have a confounding role in SCP. Even though this effect is obfuscated in the majority of published SCP datasets since they consist of relatively homogeneous cell types (e.g., epithelial cells like HeLa) with little variation in cell sizes, proteome coverage in SCP generally scales with the size of the cells studied^11^, with a significantly lower number of proteins identified and quantified in smaller cell types such as white blood cells^12^. This is likely due to technical factors such as raw MS sensitivity, as smaller cells contain fewer copies of each protein, making it harder to detect low-abundance proteins from smaller cells. Recent studies extended SCP workflows to smaller hematopoietic and immune cell populations, although proteome depth and data completeness generally remained lower than for larger epithelial cell models^5,12,13^. Moreover, even minor losses during SCP sample preparation disproportionately affect smaller cells. Therefore, further improvements in sample preparation and MS sensitivity are still required to achieve deep and reproducible proteome coverage from smaller immune cell types by SCP.

Here, we provide a systematic optimization of a label-free SCP workflow for high-sensitivity SCP, with a particular emphasis on improving SCP coverage by leveraging improved sample preparation strategies and enhanced mass spectrometry acquisition methods on the Thermo Scientific^TM^ Orbitrap™ Astral™ Zoom mass spectrometer. Based on these technological innovations, we demonstrate routine coverage of >7000 proteins in single HeLa cells, and ~4000 proteins from individual small cells prepared from human peripheral blood mononuclear cells (PBMCs), providing proteome coverage of >50% of the adaptive immunity pathways. This SCP technology outlines future directions for advancing SCP towards routine, large-scale applications in biology and medicine.

## Results

### Systematic optimization of LC-MS conditions for low load

**Fig. 1:**
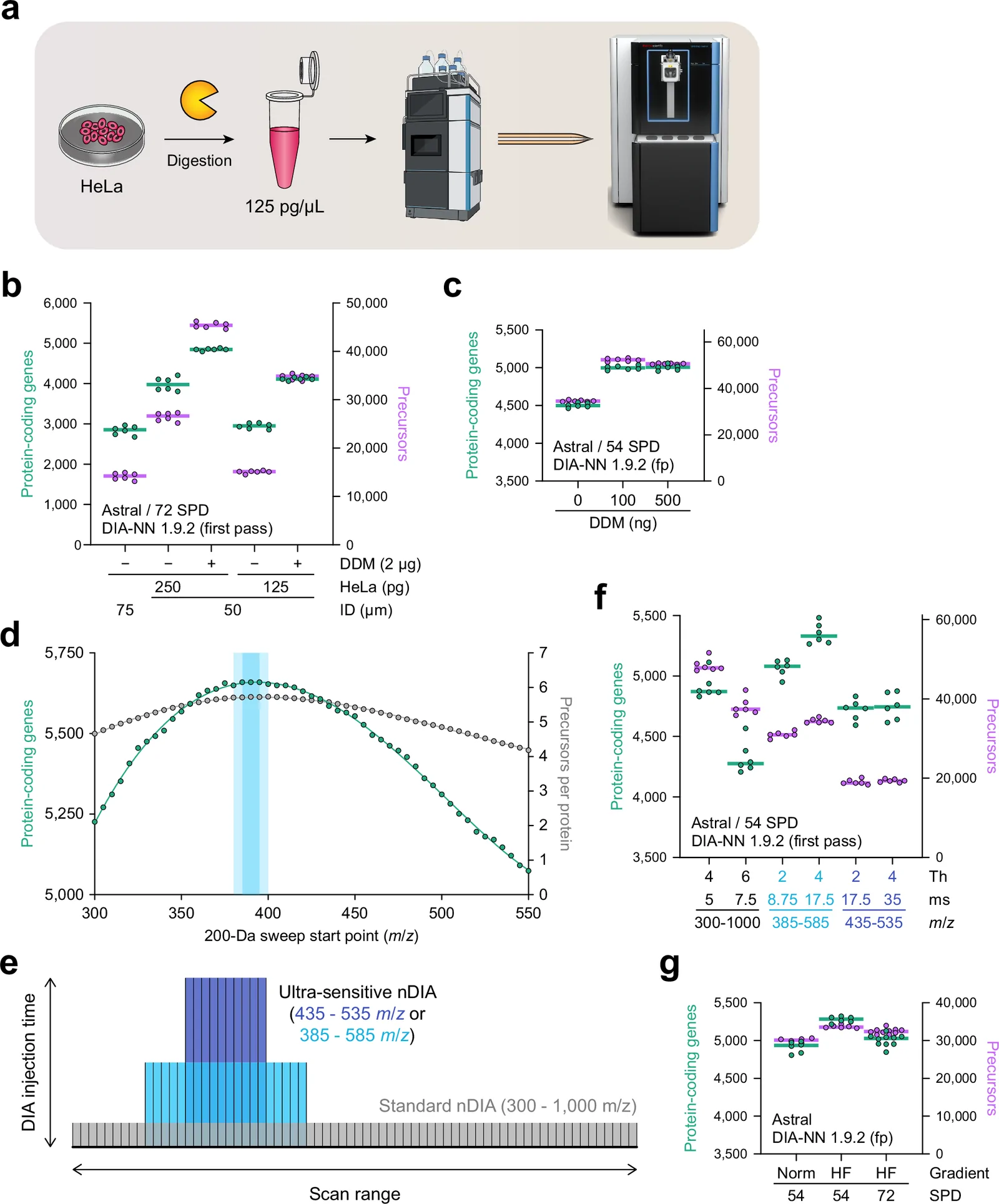
Optimization of low-input LC-MS methodology for enhanced protein identification rates. **a** Schematic overview of the experimental design. HeLa cells were lysed, digested with Lys-C and trypsin, and purified HeLa peptides were diluted to a concentration of 125 pg/µL prior to analysis using a Vanquish HPLC, laser-pulled and in-house packed analytical columns, and an Orbitrap Astral mass spectrometer. Sample tube art adapted from Servier Medical Art (https://smart.servier.com), licensed under CC BY 4.0 (https://creativecommons.org/licenses/by/4.0/). Partially created in BioRender. Olsen, J. (2026) https://BioRender.com/ygoith1. **b** Number of protein-coding genes (green, left axis) and precursors (purple, right axis) identified when comparing different column inner diameters, HeLa load, and the addition of n-dodecyl β-D-maltoside (DDM). Bars represent the median. *n* = 6 technical replicates. ID internal column diameter. Data analysis using DIA-NN (v1.9.2; throughout this study) without matching between runs. Coefficient of variation distributions are available in Supplementary Fig. 1. **c** As in (**b**), but comparing different amounts of DDM injected for analysis of 250 pg HeLa. *n* = 6 technical replicates. **d** Number of protein-coding genes (green, left axis) and precursors per protein (gray, right axis) identified when restricting identification to a 200 Da window, e.g., from 300 to 500 *m*/*z* when starting at 300 *m*/*z*, based on the data from (**b**) that was recorded from 300 to 1000 *m*/*z*. Blue highlighting indicates the local optimum range for identification of the most protein-coding genes. **e** Visualization of the ultra-sensitive narrow-window DIA (nDIA) concept, where sampling is restricted to a narrower but more proteotypic *m/z* range, while increasing maximum DIA ion injection time to enhance sensitivity. **f** As in (**b**), but comparing different ultra-sensitive nDIA methods for analysis of 250 pg HeLa. “ms”; maximum DIA ion injection time in milliseconds. *n* = 6 technical replicates. **g** As **b**, but evaluating different gradient lengths and throughput speeds for analysis of 250 pg HeLa. From left to right, *n* = 6, 6, 10 technical replicates. Norm standard steady-flow chromatography, HF method employing Higher Flow (i.e., 500 nL/min) at the start and end of the gradient to maximize effective peptide elution time, SPD samples per day.

Mass spectrometry (MS)-based SCP is an emerging field, with a plethora of sample preparation and technological improvements resulting in enhanced depth of sequencing from highly limited starting material. Here, we endeavored to systematically assess, optimize, and apply cutting-edge methodology and technology in order to maximize insight that can be gleaned from analysis of the smallest single cells. To this end, we first set out to optimize LC and MS methodology for analysis of low amounts of peptides derived from HeLa digests (Fig. 1a), using a Thermo Scientific^TM^ Vanquish™ Neo UHPLC System (Vanquish) and narrow-window data-independent acquisition (nDIA) on a Thermo Scientific™ Orbitrap™ Astral™ MS (Orbitrap Astral). A comprehensive overview of all LC-MS acquisition parameters for all experiments in this work is provided in Supplementary Data 1.

To optimize LC-MS settings for SCP, we first analyzed dilutions of a bulk tryptic digest of HeLa lysate. We compared analysis of 250 or 125 pg peptide digest (peptide content roughly equivalent to one or half of a single HeLa cell), in the absence or presence of the MS-compatible non-ionic detergent n-dodecyl β-D-maltoside (DDM), and using laser-pulled in-house packed C18 reversed-phase columns with an internal diameter (ID) of either 50 or the standard 75 µm (Fig. 1b and Supplementary Fig. 1A–C). The different column diameters were tested to take advantage of the fact that the electrospray process is concentration-dependent, and since the ideal flow rate for nanoLC is proportional to the column diameter, narrower columns support lower flow rates, resulting in greater sensitivity^14^. Notably, LC-MS analysis was performed at comparable backpressure, with flow rates of 125 or 250 nL/min for the 50 and 75 µm columns, respectively. The number of identified precursors almost doubled when using a 50 µm ID column, and was further increased by the addition of DDM to the runs. Similar increases in identifications were observed for protein-coding genes (referred to as proteins hereafter), with a 50 µm ID column facilitating identification of as many proteins from 125 pg, as a 75 µm ID column with 250 pg (Fig. 1b). Importantly, we observed essentially identical chromatographic and proteomic performance when comparing our in-house packed 50 µm ID column with a commercially available analytical column of identical dimensions and stationary phase, demonstrating that this workflow can readily be implemented without in-house column fabrication (Supplementary Note 1). Along the same lines, addition of DDM to the 50 µm ID workflow resulted in as many proteins profiled from 125 pg as compared to 250 pg without DDM. Sample loss due to adsorption at hydrophobic surfaces is a major bottleneck in SCP, but DDM can mitigate this by improving recovery of hydrophobic peptides and thereby enhancing signal intensity in MS-based SCP^15^. We reduced the amount of DDM by up to 20-fold (Fig. 1c and Supplementary Fig. 1D–F), and found that even the smallest amount tested here was sufficient to limit peptide loss and enhance identification rates, overall allowing identification of ~5000 proteins and ~50,000 precursors. Notably, substantially higher DDM amounts resulted in gradual accumulation of hydrophobic material on the LC system, whereas the optimized concentrations used throughout this study did not (Supplementary Note 2).

While we employed a conventional broad-range DIA-MS/MS scanning method (300–1000 m/z) at first, we reasoned that restricting the scan range while increasing the allowed injection time per window could enhance sensitivity while retaining proteotypicity. We scrutinized our first data to extract 200 m/z DIA-MS/MS scan ranges and determined the number of proteins within each of the scan ranges (Fig. 1d). We found that starting the scan range ~385 m/z resulted in the maximum number of protein precursors per protein. To validate this, we set up several ultra-sensitive narrow-window DIA (nDIA) methods (Fig. 1e), in all cases keeping the overall scan cycle time constant at ~1 second, and evaluated their performance on 250 pg HeLa (Fig. 1f and Supplementary Fig. 1G–I). Indeed, although we observed a drop in the number of precursors identified when using a narrow-scan range (385–585 m/z with 4-Th nDIA-MS/MS isolation windows and allowing 17.5 ms injection times), the number of proteins increased from ~4900 to ~5300, while also resulting in considerably lower coefficient of variation (Fig. 1f). Reducing the DIA-MS/MS scan range to only 100 m/z was not as efficient, and for the 200 m/z method we found that 4 Th windows were superior to 2 Th windows.

The number of samples per day (SPD) that can be analyzed is a pivotal metric for high-throughput and especially SCP, as numerous single cells need to be measured to achieve sufficient statistical power. We designed a chromatographic method for the Vanquish Neo UHPLC whereby we initiated the gradient at higher flow (i.e., 500 nL/min) and pressure, and then gradually reduced the flow prior to elution of peptides, effectively reducing column delay from 6 to 2 min. This allowed us to acquire essentially the same number of precursors and proteins in a 15 min gradient (72 SPD) compared to a 20 min gradient (54 SPD), with only a slightly increased coefficient of variation (Fig. 1g and Supplementary Fig. 1J–L). Notably, peak shape and width were minimally affected in our higher flow method (Supplementary Note 3).

Several other MS-technical parameters were evaluated, including scanning loop control and fragment ranges (Supplementary Note 4). Together, these optimizations in LC-MS methodology—encompassing column selection, surfactant-assisted peptide recovery, refined scan ranges, and accelerated chromatographic gradients—substantially improved proteome coverage and throughput from minimal sample inputs, thereby establishing a robust foundation for subsequent experiments and SCP analyses.

### Evaluating the Orbitrap Astral Zoom MS for enhanced sensitivity and proteome depth

**Fig. 2:**
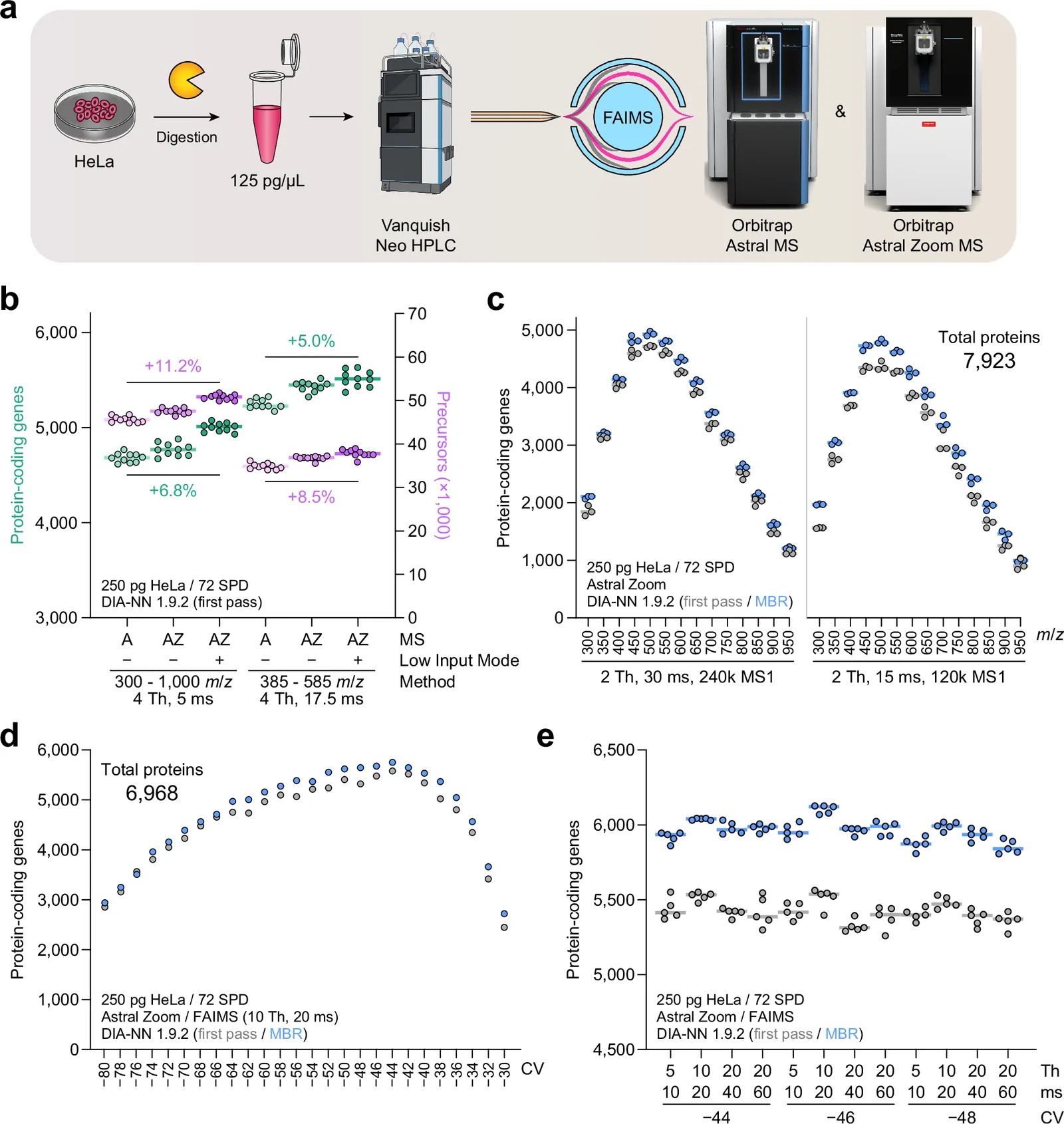
Evaluation of the Orbitrap Astral Zoom MS for low-input analysis. **a** Schematic overview of the experimental design. HeLa cells were lysed, digested with Lys-C and trypsin, and purified HeLa peptides were diluted to a concentration of 125 pg/µL prior to analysis using a Vanquish HPLC, laser-pulled and in-house packed analytical columns, and an Orbitrap Astral Zoom prototype mass spectrometer. Sample tube art adapted from Servier Medical Art (https://smart.servier.com), licensed under CC BY 4.0 (https://creativecommons.org/licenses/by/4.0/). Partially created in BioRender. Olsen, J. (2026) https://BioRender.com/ygoith1. **b** Number of protein-coding genes (red, left axis) and precursors (blue, right axis) identified, when comparing Orbitrap Astral (“A”) mode with Orbitrap Astral Zoom (“AZ”) mode, with the latter either in regular or Low-Input mode. Bars represent median, percentage increases of median values are indicated; *n* = 10 technical replicates. Data analysis using DIA-NN without matching between runs. **c** Gas-phase fractionation experiment, wherein precursors are isolated from a 50 *m/z* range starting at the *m/z* indicated on the *x* axis. Gray and blue dots correspond to the number of identified protein-coding genes with data analysis using DIA-NN first-pass ass mode and MBR mode, respectively. *n* = 3 technical replicates, total number of protein-coding genes identified cumulatively across all runs is indicated. **d** As **c**, but evaluating a range of FAIMS compensation voltages (CV), using a single constant CV across the entire gradient. Scan range was 400–800 *m*/*z*. *n* = 1 technical replicate. **e** As **d**, but investigating different combinations of DIA window size in Thomson (“Th”) and DIA ion injection time in milliseconds (“ms”) in combination with three different CVs. *n* = 5 technical replicates.

Recently, the Orbitrap Astral Zoom MS (Orbitrap Astral Zoom) was introduced commercially. This instrument incorporates several new features, including pre-accumulation^16^, higher acquisition rate (HAR) of up to 270 Hz, and enhanced spectral processing^17^. Further, the Orbitrap Astral Zoom has a low input mode (LIM), which increases sensitivity for low input samples. For this study, we had access to a prototype Orbitrap Astral Zoom that allowed us to toggle these features individually, and thus perform a fair comparison between Orbitrap Astral and Orbitrap Astral Zoom (Fig. 2a). Building on recent studies exploring low-input Orbitrap Astral acquisition strategies and FAIMS-assisted SCP workflows^4,6,11^, we systematically evaluated how these capabilities could be integrated and optimized for ultra-low-input DIA proteomics.

First, we evaluated performance when analyzing 250 pg HeLa, for both ultra-sensitive nDIA and standard methods (Fig. 2b and Supplementary Fig. 2). At the precursor level, we observed an increase of 8.5% and 11.2% when running the Orbitrap Astral Zoom with LIM, and similarly the number of identified proteins increased by 5.0% and 6.8%. The coefficient of variation was similar or slightly lower with the Orbitrap Astral Zoom (Supplementary Fig. 2), and significantly lower when using the ultra-sensitive nDIA method. We performed a more in-depth evaluation of the LIM, which is detailed in Supplementary Note 5.

We investigated optimal scan ranges for ultra-sensitive nDIA, initially approximating these empirically by determining the number of proteins within 200 m/z DIA-MS/MS scan ranges (Fig. 1d). To further validate this, we performed a gas-phase fractionation (GPF) experiment, where the scan range of each run was limited to 50 m/z, using two different nDIA acquisition speeds (Fig. 2c and Supplementary Fig. 3). Analyzing 250 pg HeLa per LC-MS run, we found that the 450–500 m/z and 500–550 m/z fractions resulted in the highest identification rates, with ~5000 proteins identified, and consistent with an optimal scan range centered between 475 and 525 m/z. Based on these experimental results, we refined our narrow acquisition window from 385–585 Th to 425–625 Th for subsequent method development; direct comparison between the two acquisition windows confirmed a modest but consistent improvement in identification performance with the refined method (Supplementary Note 6). Moreover, cumulatively ~110,000 unique peptides and ~7900 proteins could be identified across the entire GPF experiment (Supplementary Fig. 3A–C), without ever injecting >250 pg per run.

An alternative strategy of fractionation in the gas phase is through application of high-field asymmetric ion mobility spectrometry using the FAIMS Pro Duo interface (FAIMS), and utilizing different compensation voltages (CVs), which impart selectivity mediated by mass-to-charge and mobility properties of precursors. We performed a FAIMS CV sweep by analyzing 250 pg of HeLa using single CVs in steps of 2 V ranging from −80 to −30 V (Fig. 2d and Supplementary Fig. 4), both to determine an optimal CV range, and to investigate the complementarity of a multi-CV fractionation approach. Although there was some variance between data analysis with DIA-NN and Spectronaut, as well as at the precursor and protein levels, CVs of −44, −46, and −48 V were proximate to the optimum (Fig. 2d and Supplementary Fig. 4A–C). Cumulatively, we identified ~90,000 unique peptides and ~7900 proteins using Spectronaut (Supplementary Fig. 4B, C). Interestingly, there was a direct linear correlation between the number of observed tryptic missed cleavages in identified peptides and the compensation voltage applied (Supplementary Fig. 4D). In terms of CV fraction complementarity, neighboring CVs demonstrated a high Pearson correlation (Supplementary Fig. 4E), suggesting that large jumps in CV are necessary for maximum orthogonality. Finally, to determine an optimal CV to use for further experiments, we performed replicate analyses using CVs of −44, −46, and −48 V and four different combinations of isolation window and maximum injection times (Fig. 2E and Supplementary Fig. 5). Based on this, we did not observe large differences between the CVs, but found that −46 V with a 10 Th/20 ms method yielded the highest number of identifications. However, the coefficient of variation was the lowest using a 20 Th/40 ms method (Supplementary Fig. 5B, C).

Collectively, these experiments demonstrate that the Orbitrap Astral Zoom MS, particularly when operated in LIM, provides measurable gains in proteome coverage, sensitivity, and reproducibility, thereby advancing the analytical capabilities of low-input proteomics.

### Defining the limit of detection and quantitative precision at ultra-low input

The amount of protein derived from a single HeLa cell is typically assumed to be in the range of 200 to 250 pg^18,19^. However, even within an asynchronous population of HeLa cells, there are considerable differences in cell size and shape. Moreover, many other cell types can have dramatically different sizes. Total protein amount per cell increases proportionally with cell size, and protein coverage in SCP scales positively with cell size due to both more total protein and higher abundance of detectable proteins, in addition to technical factors such as improved extraction and detection sensitivity. Thus, to anticipate analysis of smaller and more challenging cells, we performed a series of HeLa dilution experiments to determine the limit of detection of our LC-MS setup, as well as to further optimize our methodology in the context of ultra-low input amounts.

**Fig. 3:**
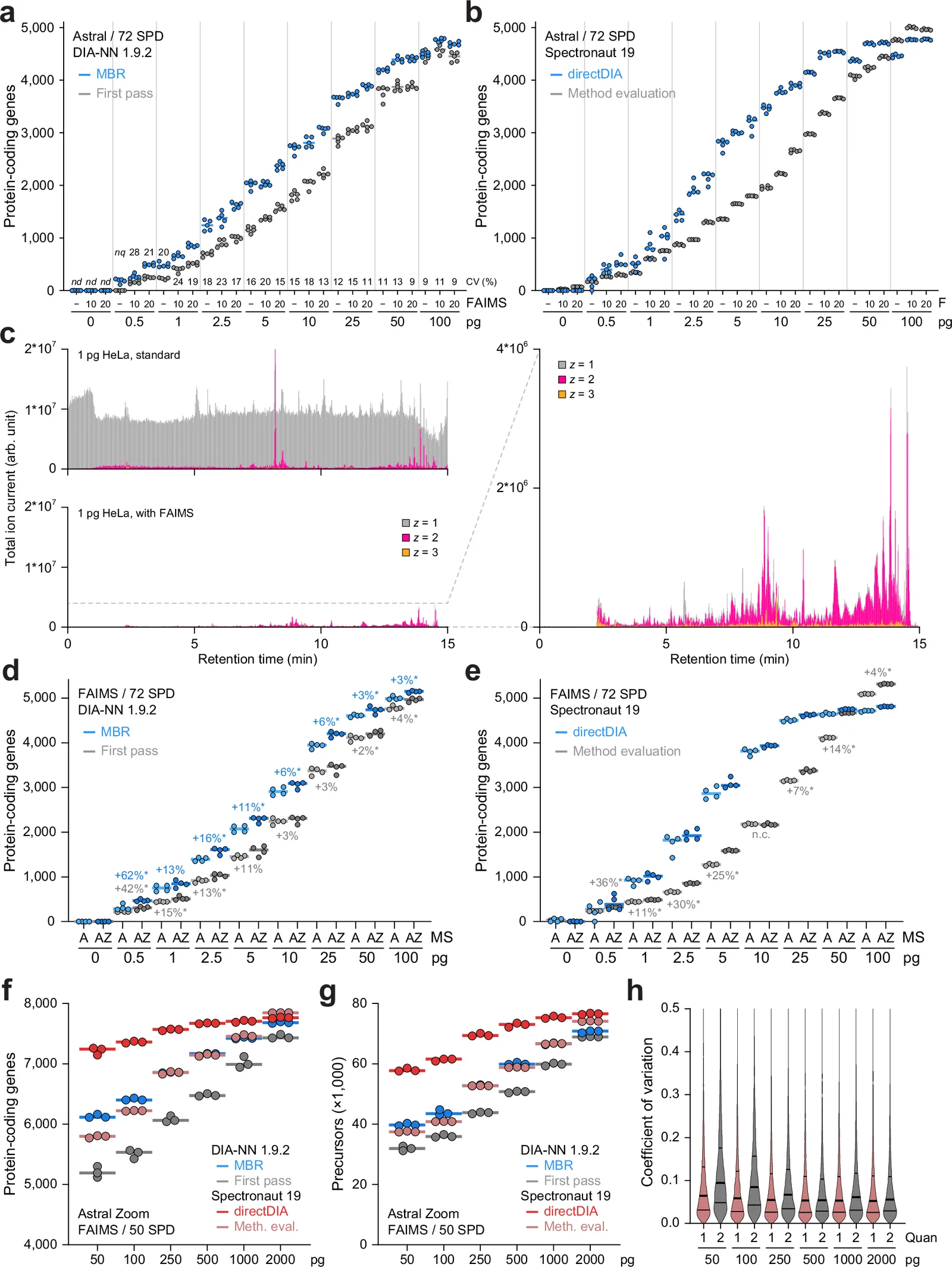
Orbitrap Astral Zoom MS and FAIMS are superior for ultra-low load analysis. **a** Dilution series experiment, ranging from 0.5 pg to 100 pg of injected HeLa lysate, comparing standard analysis to FAIMS analysis. All injections were performed in ascending amounts, starting with a blank to ensure negligible sample carryover. The number of identified protein-coding genes is indicated; bars represent the median, with gray and blue corresponding to data analysis using DIA-NN in first pass mode and MBR mode, respectively. Numbers above the *x* axis represent median coefficient of variation percentage, *n* = 5 technical replicates. “F”; indicator for analysis strategy with “−”; no-FAIMS, “10”; with FAIMS using 10 Th window width and 20 ms DIA injection time, “20”; with FAIMS using 20 Th window width and 40 ms DIA injection time. **b** As **a**, but data analysis using Spectronaut v19, with gray and blue corresponding to data analysis using Method Evaluation mode and directDIA mode, respectively. *n* = 5 technical replicates. **c** Total extracted ion current (XIC) profile across the chromatographic gradient, for a representative 1 pg run without FAIMS (top) or when using FAIMS (bottom). Ion current was stratified by charge state 1–3, with unknown or 4+ charge states omitted from the visualization. The plot on the right side is a zoomed-in version of the FAIMS run, highlighting the almost total absence of atmospheric contamination (*z* = 1). **d** Dilution series experiment as described in (**a**), but using FAIMS in all cases and comparing the difference between the Orbitrap Astral (“A”) and Orbitrap Astral Zoom (“AZ”) using a prototype mass spectrometer. Percentage increases of median values are indicated, * = *p* < 0.05 via two-tailed Student’s *t* testing, *n* = 4 tech*n*ical replicates. **e** As **d**, but data analysis using Spectronaut v19, with gray and blue corresponding to data analysis using Method Evaluation mode and directDIA mode, respectively. Differences of medians were not assessed in directDIA mode due to the non-linear behavior of the directDIA identification strategy. *n* = 4 tech*n*ical replicates. **f** Dilution series experiment as described in (**a**), injecting between 50 and 2000 pg of HeLa lysate on a 50 SPD gradient, and using MS and FAIMS methodology optimized for MS1-based quantification and for each sample load. Number of identified protein-coding genes is shown for data analyses using either DIA-NN or Spectronaut. Bars represent median, *n* = 3 tech*n*ical replicates. **g** As **f**, but showing the number of precursors (DIA-NN) or stripped peptide sequences (Spectronaut). *n* = 3 tech*n*ical replicates. **h** Violin plot distribution of coefficient of variation for all protein-coding genes from (**f**). MS1-based quantification via Spectronaut (method evaluation), MS2-based quantification via DIA-NN (first pass). Thick line represents median, thin lines 1st and 3rd quartiles. *n* = 3 technical replicates.

Utilizing the low-input methodology we established for analysis without and with FAIMS (Figs. 1 and 2), we compared the performance of the methods over a dilution series starting at 100 pg and going down to as little as 0.5 pg (Fig. 3a, b and Supplementary Fig. 6A, B). Down to 50 pg, we did not observe a notable difference between the methods when performing a first-pass analysis using DIA-NN (Fig. 3a), demonstrating that both no-FAIMS and FAIMS approaches can be efficiently applied to loads above 50 pg. Of note, the FAIMS approaches performed slightly better when analyzing the data using Spectronaut (Fig. 3b), indicating that different software can lead to distinct conclusions. Consistently, when analyzing 25 pg or less, the FAIMS methodology started to outperform the no-FAIMS methodology, with up to ~twofold increases in the number of identified proteins at 0.5–2.5 pg loads. Quantitatively, coefficients of variation were highly comparable between the ultra-sensitive nDIA method and the FAIMS-specific MS acquisition method using 20 Th DIA windows and maximum allowed 40 ms injection time, demonstrating similar quantitative precision. We also assessed quantitative accuracy for nDIA, and found that it is minimally impacted when restricting scan range to 425–625 m/z (Supplementary Note 7). In addition, direct comparison with broader 300–1000 m/z and intermediate 400–800 m/z acquisition strategies confirmed that the 425–625 m/z method provides the best overall balance between protein identification depth and quantitative precision under low-input conditions (Supplementary Note 8). Finally, to illustrate the effect of FAIMS in the context of ultra-low loads, we investigated the charge state distribution within the total ion current in two representative 1 pg HeLa runs, either with no-FAIMS analysis or FAIMS analysis (Fig. 3c). Without FAIMS, the vast majority of ions were singly charged and likely chemical background noise, with a high polysiloxane presence^20^. With FAIMS operated at optimal single CV of −46 V, singly charged precursors were almost entirely abolished, leaving predominantly doubly and triply charged peptide precursors.

Next, we investigated the gain in performance across the dilution series using our Orbitrap Astral Zoom MS prototype, comparing regular Orbitrap Astral mode versus Orbitrap Astral Zoom mode with all its features enabled (Fig. 3d, e and Supplementary Fig. 6C–H). In the range from 10 to 100 pg, we observed consistent increases of 5–9% at the precursor level and 2–4% at the protein level when using DIA-NN in first pass mode (Fig. 3d and Supplementary Fig. 6C). In the 1–5 pg range, precursors increased by 9–12% and proteins increased by 11–15%. At the lowest quantified load of 0.5 pg, increases in the range of 40–60% were observed; however, these occurred at the very limit of detection. Similar increases were found with Spectronaut data analysis (Fig. 3e and Supplementary Fig. 6D), although more sporadically depending on input amount. Quantitatively, when inspecting coefficient of variation of the MS2-level quantification, we found that the Orbitrap Astral Zoom was able to achieve similar coefficient of variation with approximately half the input amount as compared to the Orbitrap Astral (Supplementary Fig. 6E, F). Pearson correlations between same-load replicates were comparable or slightly enhanced with the Orbitrap Astral Zoom (Supplementary Fig. 6G, H).

Quantitative precision and reproducibility are important tenets of proteomics method design. We have primarily performed MS2-based quantification throughout this study; however, a core strength of the Orbitrap Astral and Orbitrap Astral Zoom is that they can dedicate the Orbitrap mass analyzer to MS1-based quantification without sacrificing the ability to sequence precursors in parallel using the Astral mass analyzer^6^. To exemplify the capability of the Orbitrap Astral Zoom to leverage MS1-based quantification, we performed another dilution series experiment ranging from 2000 pg down to 50 pg of HeLa, using 50 SPD gradients and FAIMS (Fig. 3f, g). From 50 pg, we could identify ~5100 proteins using DIA-NN in first-pass mode, and up to ~7200 proteins when co-analyzing the 50 pg runs together with all other loads using Spectronaut in directDIA mode. Importantly, using MS1-based quantification, we achieved very low median coefficients of variation of 5.2–6.5% at the protein level and 10.4–11.8% at the precursor level (Fig. 3h and Supplementary Fig. 6I).

Overall, our data showed that the combination of FAIMS and the Orbitrap Astral Zoom enables reproducible and accurate proteome profiling from protein inputs far below that of a typical single HeLa cell, paving the way for analysis of smaller or less protein-rich cells.

### Optimization of the CellenOne-based SCP workflow

**Fig. 4:**
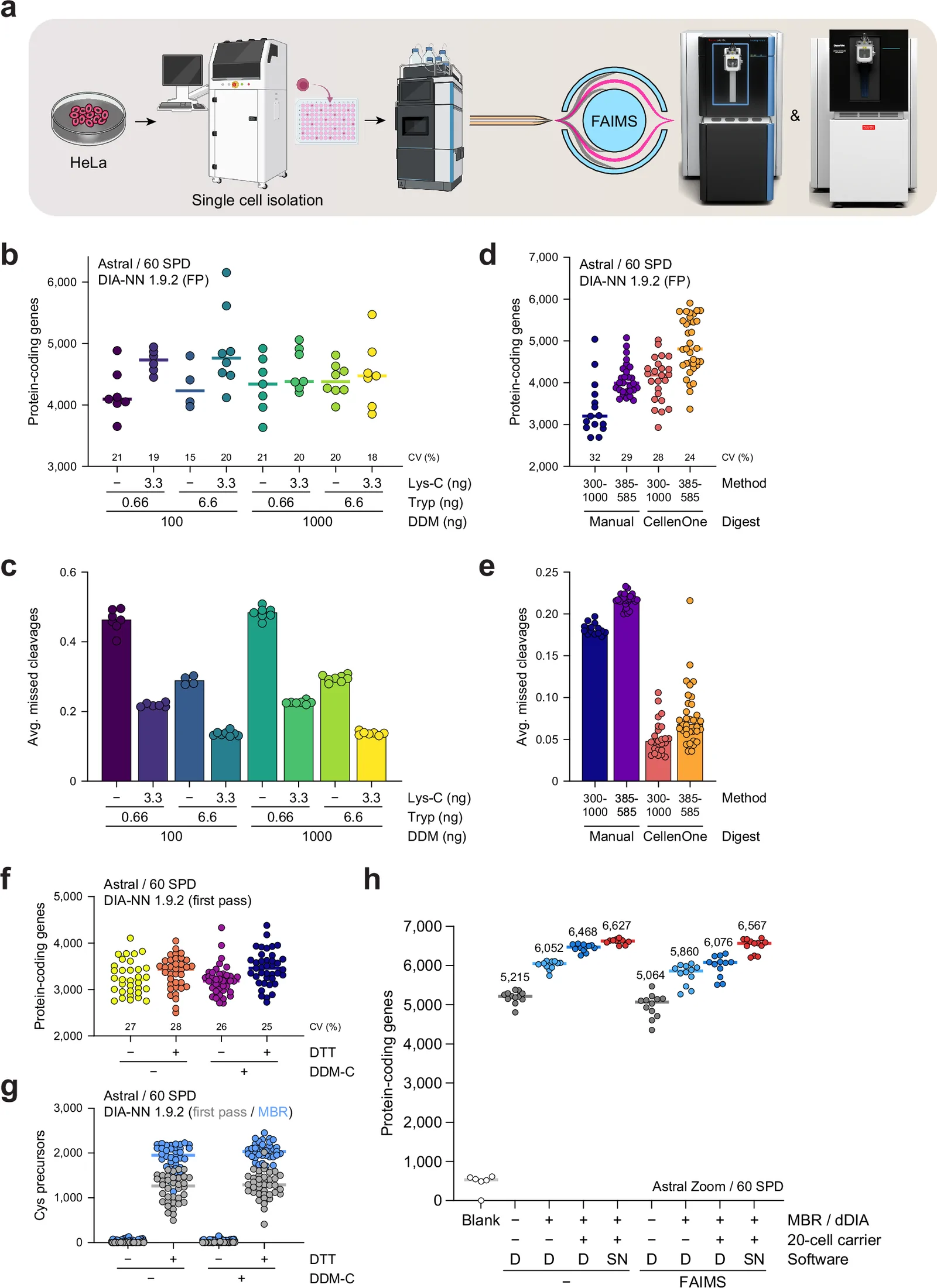
Optimization of single-cell proteomics MS methodology for standard and FAIMS workflows. **a** Schematic overview of the experimental design. HeLa cells were grown, single cells were sorted into and digested in 96-well plates using the CellenOne platform, and digests were directly analyzed using a Vanquish HPLC, laser-pulled and in-house packed analytical columns, and an Orbitrap Astral Zoom mass spectrometer prototype. Partially created in BioRender. Olsen, J. (2026) https://BioRender.com/ygoith1. **b** Number of protein-coding genes identified from single HeLa cells when using various digestion mixes. Cells were sorted by the CellenOne platform, but otherwise manually digested. Bars represent the median. Numbers above the *x* axis are the median coefficient of variation. From left to right, *n* = 7, 6, 4, 8, 7, 7, 8, 7 single cells. **c** As **b**, but showing the average number of missed tryptic cleavages observed per peptide. From left to right, *n* = 7, 6, 4, 8, 7, 7, 8, 7 single cells. **d** As **b**, but comparing manual digestions (with the CellenOne only used to dispense single cells) to fully automated handling and digestion using the CellenOne, as well as comparing the broader- scan range nDIA method with ultra-sensitive narrow-scan range nDIA. From left to right, *n* = 15, 28, 24, 35 single cells. **e** As **d**, but showing the average number of missed tryptic cleavages observed per peptide. From left to right, *n* = 15, 28, 24, 35 single cells. **f** As **b**, but additionally comparing the effect of dithiothreitol (DTT) in the digestion mix, as well as pre-coating the 96-well plate with n-dodecyl-ß-D-maltoside (DDM). From left to right, *n* = 33, 36, 40, 39 single cells. **g** As **f**, but showing the number of cysteine-containing precursors identified in the data. From left to right, *n* = 33, 36, 40, 39 single cells. **h** Number of protein-coding genes identified from single HeLa cells (median diameter ~26.5 μm) when using optimized sample preparation workflows, computational inclusion of 20-cell carriers, and using either standard- or FAIMS-optimized methodology. Data analysis using either DIA-NN (“D”) or Spectronaut (“SN”) as indicated. *n* = 6 blanks and 12 single cells. MBR DIA-NN matching-between-runs, dDIA Spectronaut directDIA.

Having optimized and benchmarked our LC-MS methodology using a diluted HeLa lysate, we next sought to adapt these advances to the analysis of individual, physically sorted and isolated HeLa cells. SCP introduces unique experimental challenges, including limited material, surface adsorption, and variability in lysis and digestion efficiency. To address these, we established and evaluated a single-cell workflow using the CellenOne for cell isolation and digestion (Fig. 4a). Notably, for all SCP experiments in this study, we did not perform peptide clean-up, and digested single cells were directly injected onto the analytical LC column for online LC-MS analysis.

We first performed an evaluation of different compositions of the combined cell lysis, protein extraction, and digestion buffer (e.g., master mix), wherein we varied concentrations of DDM, trypsin, and Lys-C (Fig. 4b, c and Supplementary Fig. 7A–C). For this experiment, we manually performed deposition of the master mix and digestion, whereas single-cell isolation into the wells was performed using a CellenOne. Overall, we were able to identify between 4000 and 5000 proteins per cell using DIA-NN in first pass mode (Fig. 4b), and up to ~5500 proteins per cell when using match-between-run (MBR) mode (Supplementary Fig. 7B), regardless of master mix composition, although there was a trend for higher identification rates when both Lys-C and trypsin were present in the master mix. Notably, when considering missed tryptic cleavage rate (Fig. 4c), we observed a very consistent digestion efficiency for all cells digested with the same master mix, with the Lys-C and trypsin combination necessary for achieving the lowest fraction of missed tryptic cleavage rates.

Next, we compared manual dispensing of master mix and digestion with fully automated processing using the CellenOne, which was used for single-cell isolation in both cases (Fig. 4d, e and Supplementary Fig. 7D–H). For each of the approaches, we analyzed single cells using either regular nDIA or ultra-sensitive nDIA methods. Consistent with what we observed with diluted HeLa lysate (Fig. 1f), we identified fewer precursors, but considerably more (~15–25%) proteins when using the ultra-sensitive nDIA method (Fig. 4d). Strikingly, using the CellenOne for all steps of the single-cell processing dramatically reduced the number of missed cleavages from ~20% to ~5% (Fig. 4e), with a concomitant increase of 20–30% more proteins identified per cell (Fig. 4d). With computational inclusion of a 20-cell carrier and using DIA-NN in MBR mode, we were able to identify a median of ~6000 proteins per cell (Supplementary Fig. 7G).

Cysteine disulfide bridge reduction and alkylation of proteins is not routinely performed in SCP workflows^3,11^, due to the challenges associated with sample loss, introduction of chemical contaminants, and the potential for off-target chemical modifications when handling minute amounts of protein. As our workflow does not include a peptide clean-up step, we were limited to using only volatile or otherwise MS-compatible reagents. We reasoned that reduction during SCP digestion using dithiothreitol (DTT) would be sufficient to reduce cysteine residues until stabilization via acidification of the digest. We performed a manual master mix dispensing and single-cell digestion step, wherein we compared the absence or presence of DTT, as well as the effect of coating the 96-well plate in DDM prior to master mix deposition (Fig. 4f, g and Supplementary Fig. 8A–G). Coating the 96-well plate with DDM had no discernible effect, likely because the master mix itself also contained DDM. Addition of DTT resulted in an ~8% increase at the protein level (Fig. 4f), and an ~18% increase at the precursor level (Supplementary Fig. 8A). Expectedly, in the absence of DTT, we could not detect any cysteine-containing precursors, whereas up to ~2000 cysteine-containing peptide precursors were readily detectable with DTT (Fig. 4g).

Finally, we compared analyzing single HeLa cells on the Orbitrap Astral Zoom using either standard or FAIMS acquisition (Fig. 4h and Supplementary Fig. 8H, I). Overall, we identified ~10% fewer precursors when using FAIMS (Supplementary Fig. 8H), but a comparable number of proteins (Fig. 4h), with ~5100 proteins per cell when using DIA-NN in first-pass mode, and ~6500 proteins per cell when including 20-cell carriers and using either DIA-NN in MBR mode or Spectronaut in directDIA mode. Coefficients of variation were not notably different between standard and FAIMS methods (Supplementary Fig. 8I). Using blank injections between single-cell runs, only a few hundred proteins were identified, indicating a run-to-run carryover of ~0.4% (Supplementary Fig. 8J). We also performed an investigation of the computational proteome carryover effect via co-analysis of single or several HEK cells, as described in Supplementary Note 9.

Taken together, our results highlight key parameters that govern SCP performance, including enzyme composition, reduction conditions, and automation of sample handling. The resulting CellenOne-based workflow delivers high digestion efficiency, minimal carryover, and proteome coverage exceeding 6000 proteins per cell at a throughput of 60 SPD, underscoring its suitability for sensitive and reproducible SCP.

### Ultra-deep profiling of single HeLa cells uncovers low-abundant proteins

**Fig. 5:**
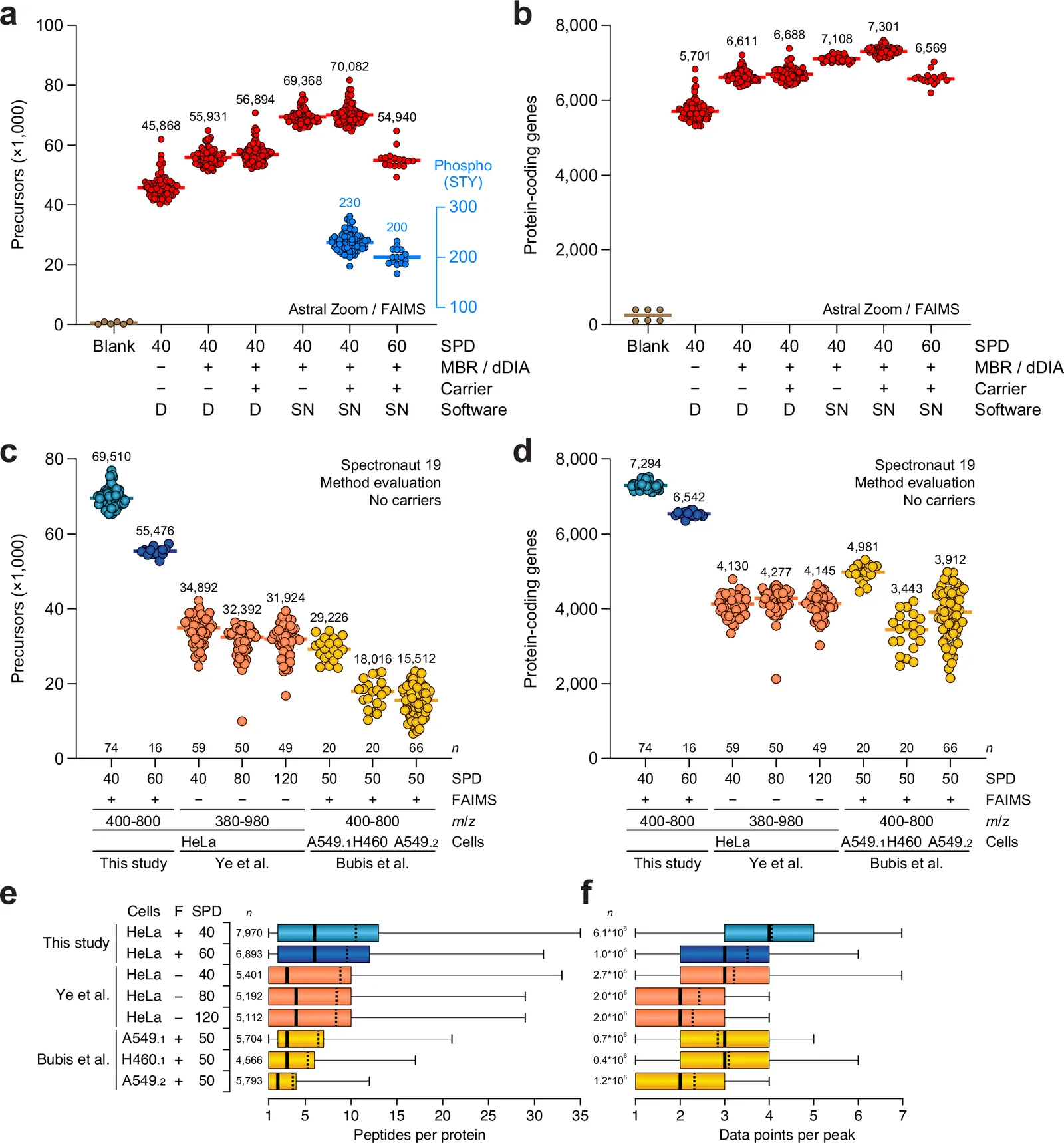
Robust profiling of >7000 proteins from single HeLa cells. **a** In red, number of precursors (DIA-NN) or stripped peptide sequences (Spectronaut) identified from single HeLa cells (median diameter ~26.5 μm). In blue, number of localized ( > 0.75 probability) phosphorylation sites identified from single HeLa cells. Automated single-cell isolation and digestion using the CellenOne, all analysis on Orbitrap Astral Zoom MS with FAIMS, comparing 40 and 60 samples-per-day (SPD) gradients. Bars represent median values; exact medians are displayed above each group of data. Data analysis using DIA-NN (“D”) or Spectronaut (“SN”) as indicated. “Carrier”; computational carrier of 20, 40, and 60 cells. “MBR”; DIA-NN matching-between-runs; “dDIA”; Spectronaut directDIA. *n* = 74 single cells (for 40 SPD) and *n* = 16 single cells (for 60 SPD). **b** As **a**, but for the number of protein-coding genes. *n* = 74 single cells (for 40 SPD) and *n* = 16 single cells (for 60 SPD). **c** Comparison between the number of precursors identified from single cells in this study, to two recent landmark papers using similar single-cell approaches^3,11^. Publicly available raw MS data files were downloaded and re-processed in one computational run using Spectronaut in Method Evaluation mode. Bars represent median values, exact medians displayed above each group of data. Number of replicate single cells (*n*) is displayed above the *x* axis. Appendix numbers following cell type indicate two separate batches of A549 cells from Bubis et al. **d** As **c**, but for protein-coding genes. **e** As **d**, quantifying the number of peptides identified per protein-coding gene, visualized as a box plot. Thick line; median, dashed line; average, box limits; 1st and 3rd quartile, whisker limits; 5th and 95th percentile. *n* = the total number of cumulative protein-coding genes identified within each cell group, as indicated. **f** As **e**, but visualizing the number of datapoints per peptide peak, with higher numbers of data points allowing more precise quantification. *n* = the total number of traceable peaks within the entire cell group, as indicated.

Having established an efficient workflow for isolation and digestion of single cells, as well as enhanced MS methodology for analysis of low input amounts, our next goal was to achieve comprehensive depth of sequencing within single HeLa cells while also maintaining good quantitative precision. For this, we performed another series of single HeLa experiments based on our optimized CellenOne-based protocol. For the SCP LC-MS analysis, we utilized FAIMS in combination with extended gradients (40 SPD), bolstered by the enhanced sensitivity of the Orbitrap Astral Zoom MS. From single HeLa cells and without inclusion of carriers, we were able to identify ~56,000 or ~69,000 peptides, and ~6600 or ~7100 proteins, with DIA-NN or Spectronaut, respectively (Fig. 5a, b). Computational inclusion of carriers was able to boost the number to ~7300 proteins from single HeLa cells. Considering our depth of sequencing, we performed a database search for phosphorylation as a variable modification on serine, threonine and tyrosine residues^21^. Although we used a partially restricted acquisition range of 400–800 m/z, which may not necessarily be optimal for peptide-level analyses such as phosphopeptide characterization, we were nonetheless able to identify a median of 230 localized phosphorylation sites in single HeLa cells (Fig. 5a), which is about twice as high as observed previously^3^. Reassuringly, phosphopeptide identifications displayed C-score and *q* value distributions comparable to, or slightly better than, unmodified peptides and other commonly observed peptide modifications (Supplementary Fig. 9A, B), supporting the overall confidence of phosphosite identification despite the absence of phosphopeptide-specific enrichment.

Two landmark SCP studies were recently published, which used comparable approaches and MS instrumentation applied to human epithelial cancer cell lines like HeLa and A549^3,11^. To benchmark our optimized SCP workflow described here, we performed a direct comparison of our SCP data to theirs, via re-analysis of our MS data together with their publicly available raw MS data. We searched all data using the same settings to minimize computational variance, and we excluded carrier channels, as the numbers and types of carriers were highly irregular between studies. Reassuringly, we found similar numbers of precursors and proteins from our own data even when searched with slightly different settings and together with data from other studies (Fig. 5c, d and Supplementary Fig. 9C–F). From our 40 SPD runs, we identified two- to three-fold more precursors, and 2000–3000 more proteins from single cells compared to the two landmark studies. Notably, we also achieved greater numbers of identifications from our 60 SPD runs. To assess the quantitative capability of our method, we investigated peptides-per-protein as well as data points per peak, and compared this across studies. With both our 40 and 60 SPD methods, we achieved a median of six peptides per protein, whereas other studies only identified two to four peptides per protein (Fig. 5e). In terms of datapoints per peak, we achieved a median of three to four points per peak for 60 and 40 SPD, respectively, with other studies generally getting two to three points per peak (Fig. 5f). Together, these results demonstrate that both qualitatively and quantitatively we achieve considerably greater performance compared to contemporary methodology.

**Fig. 6:**
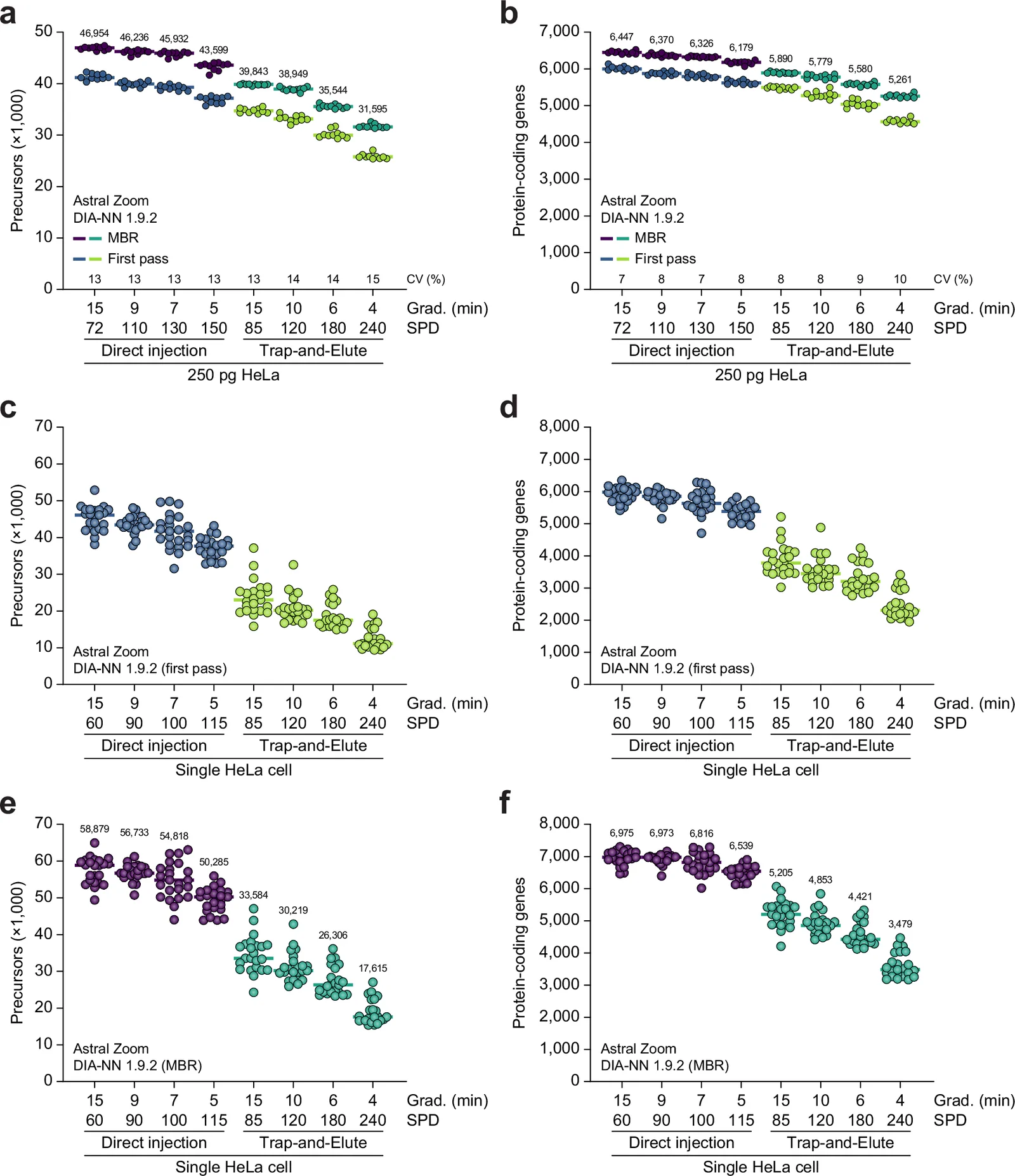
Deep single-cell proteome coverage is maintained across high-throughput workflows. **a** Number of precursors identified from 250 pg HeLa lysate using direct injection or trap-and-elute chromatography configurations across progressively higher-throughput workflows. All analyses were performed using the Orbitrap Astral Zoom MS, and data analyzed using DIA-NN in either first-pass or matching-between-runs (MBR) mode. Bars represent median values, exact medians (with MBR) are displayed above each group of data. Numbers above the *x* axis represent the median coefficient of variation percentage; *n* = 10 technical replicates. “Grad.”; analytical gradient length, “SPD”; samples-per-day. **b** As **a**, but for protein-coding genes. *n* = 10 technical replicates. **c** As **a**, but for single HeLa cells (median diameter ~26.7 μm), with automated single cell isolation and digestion using the CellenOne. Data analyzed using DIA-NN in first-pass mode. *n* = 23 single cells per condition. **d** As **c**, but for protein-coding genes. *n* = 23 single cells per condition. Blank injections (*n* = 2 per method) analyzed alongside the single-cell throughput experiments yielded minimal identifications (0–7 protein-coding genes; see Source Data). **e** As **c**, but data analyzed with DIA-NN in MBR mode and inclusion of a computational 20-cell carrier measured for each method. *n* = 23 single cells per condition. **f** As **e**, but for protein-coding genes. *n* = 23 single cells per condition.

As recent SCP studies have increasingly emphasized higher-throughput acquisition regimes up to 120 SPD^3^, we next investigated how our optimized ultra-sensitive nDIA workflow scaled toward progressively higher-throughput operation (Fig. 6). We considered both direct injection, the HPLC methodology used so far in this work, and trap-and-elute chromatography configuration, which facilitates substantially higher sample throughput by fast sample loading onto a reusable pre-column^4,14,17,22^. For analysis of 250 pg HeLa lysate, direct injection enabled acquisition at up to 150 SPD by shortening the analytical gradient from 15 to 5 min, while still identifying ~44,000 precursors and ~6200 proteins (Fig. 6a, b), or ~93% and ~96% compared to the 72 SPD method, respectively. Using trap-and-elute, throughput could be further increased to 240 SPD using a 4 min analytical gradient (Fig. 6a, b), with ~32,000 precursors and ~5300 proteins, or ~79% of precursors and ~89% of proteins compared to the 85 SPD method. Notably, when using matched 15 min analytical gradients, direct injection generally yielded higher numbers of precursors and proteins compared to trap-and-elute acquisition, and even the 5 min direct injection gradient enabled identification of more precursors and protein genes compared to the 15 min trap-and-elute gradient (Fig. 6a, b). Quantitative precision remained robust across all throughput conditions, with median protein CVs below 10% (Fig. 6b).

We next evaluated the same acquisition strategies in the context of single HeLa cells (Fig. 6c–f). Here, due to the larger injection volumes (~4 µL) required for SCP samples when using our workflow, direct injection throughput was modestly reduced compared to peptide dilution experiments, whereas trap-and-elute throughput stayed constant. Nevertheless, using direct injection, we were able to identify a median of ~7000 proteins from single HeLa cells at 60 SPD, with similar performance maintained at 90 SPD, and still identified ~6800 and ~6500 proteins at 100 and 115 SPD, respectively (Fig. 6c–f). Compared to the 250 pg HeLa lysate experiments, the reduction in protein identifications with increasing throughput was also observed for the trap-and-elute setup in the context of single-cell analysis, with the number of identified proteins decreasing gradually from ~5200 to ~3500 per cell when going from 85 SPD to 240 SPD (Fig. 6c–f). Overall, these experiments demonstrate that our SCP workflow can be configured across a broad range of analytical throughput regimes while maintaining substantial proteome depth and quantitative robustness.

**Fig. 7:**
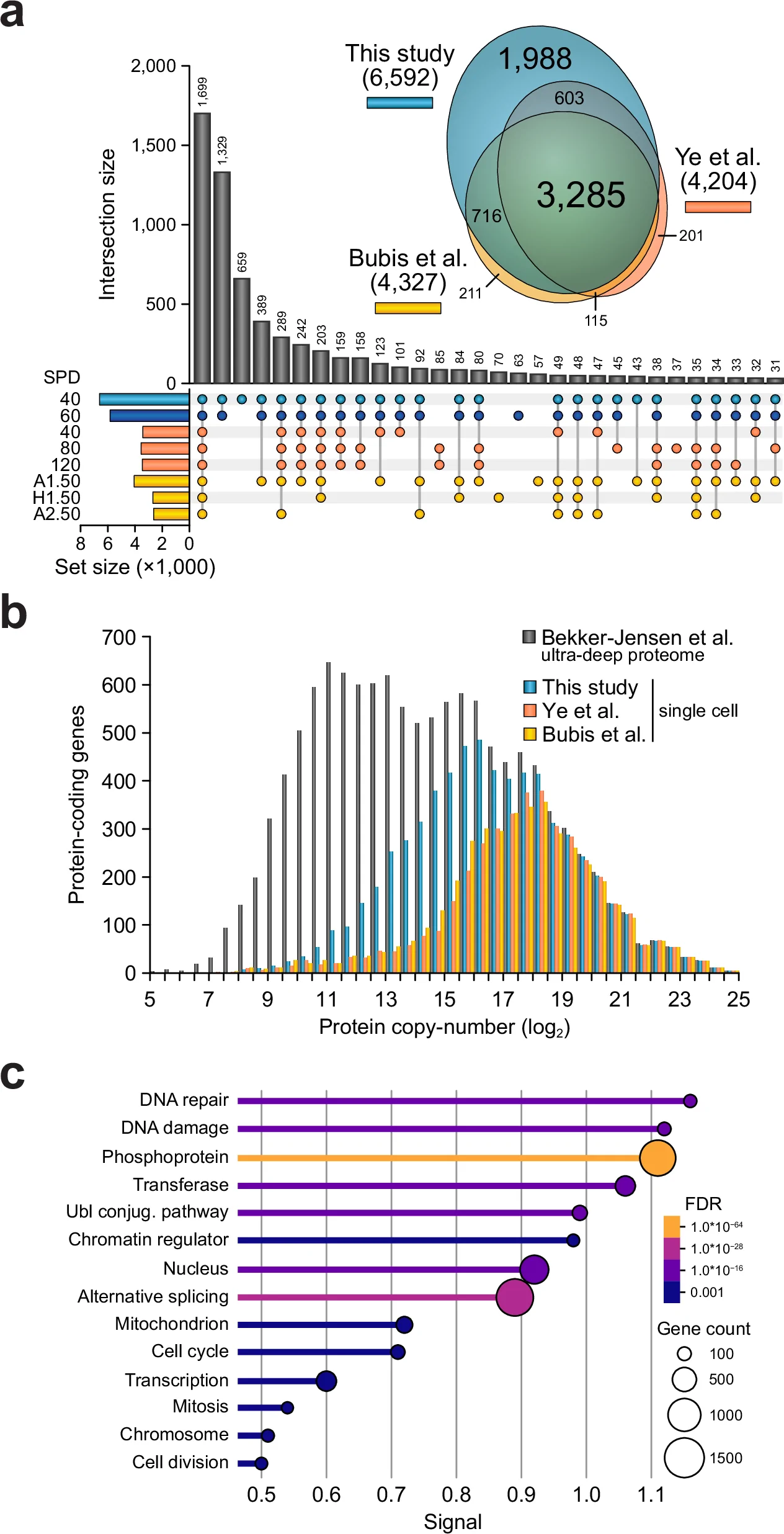
Greatly enhanced single-cell proteome depth compared to contemporary single-cell studies. **a** UpSet plot analysis, visualizing distinct and overlapping sets of proteins identified from single HeLa cells in this study compared to two recent studies using similar single-cell approaches^3,11^, see also Fig. 5c–f. Exact values are displayed above each bar. Analysis based on re-processing of available MS data using Spectronaut in Method Evaluation mode, only proteins identified by 2 or more peptides and detected in at least 50% of replicates were considered. All single cells were HeLa, except for “A1”; A549 batch 1, “H1”; H460 batch 1, “A1”; A549 batch 2. The inset scaled Venn diagram depicts global overlap between the three studies. **b** Depth of sequencing visualization, with known protein copy numbers taken from a published ultra-deep proteome. Proteins identified in all single-cell studies were mapped to the total proteome and binned in 0.5 log_2_ bins. **c** Uniprot keyword enrichment analysis for the 1988 protein-coding genes uniquely identified in this study (see **a**), querying generic Uniprot keywords via the STRING database^50^. “Signal” is an admixture of both term enrichment and FDR, as reported by the STRING database. All top terms and several other representative (non-duplicate) terms are shown; for the full list of enriched terms, refer to Supplementary Data 4. FDR and protein-coding gene count for each term are indicated.

To evaluate the biological implications of our deep profiling of single HeLa cells (Fig. 5), we examined which specific proteins we identified in comparison to the two other studies^3,11^. To this end, we performed a Venn comparison between all three datasets, and also an UpSet analysis to highlight overlapping sets of proteins across all different conditions (Fig. 7a, Supplementary Fig. 10A, and Supplementary Data 2). Expectedly, when considering proteins identified by at least two peptides and in at least half of single cells (Fig. 7a), the largest overlapping set of proteins was shared among all studies (3285 proteins), and even across all conditions (1699 proteins). The second- and third-largest intersections were represented by proteins exclusively identified in this study, followed by a mixture of intersections dependent on study size and technical parameters. Next, we evaluated the depth of sequencing achieved by us and others (Fig. 7b and Supplementary Fig. 10B), through mapping all identified proteins to copy numbers determined in an ultra-deep HeLa proteome study^23^. As anticipated, all SCP studies identified all of the most abundant proteins, regardless of technical conditions and cell type. Strikingly, we were able to identify and quantify proteins with significantly lower copy numbers compared to other studies, in some cases detecting three to five times more low-abundance proteins (Fig. 7b). When considering all proteins, even those identified by a single peptide, largely the same patterns were observed (Supplementary Fig. 10). Overall, we were able to identify 1988 proteins of below-average abundance that were not consistently identified by other contemporary SCP studies. Intriguingly, when inspecting Uniprot keywords associated with these proteins through term enrichment analysis, we found a significant overrepresentation of biologically relevant terms such as DNA repair, phosphoproteins, chromatin regulation, cell cycle, and proteins with nuclear and chromosomal localization (Fig. 7c).

Collectively, this work pushes the frontier of SCP, achieving among the deepest label-free proteome coverage from individual HeLa cells reported to date. By integrating enhanced LC-MS gradients and methodology, FAIMS, and high-sensitivity Orbitrap Astral Zoom MS detection, we resolve >7000 proteins per single HeLa cell, including ~2000 previously undetectable lower copy-number proteins. Our approach not only redefines the analytical depth achievable in single-cell studies but also opens new avenues for probing the regulatory proteome underlying cell identity and function.

### Ultra-sensitive SCP captures the immune landscape of PBMCs

Very small cells ( < ~10 µm in diameter) remain comparatively underrepresented in SCP analyses. This is because their low protein content often results in reduced proteome depth and data completeness, a problem that is exacerbated by the cubic relationship between cell diameter and protein yield, where small decreases in diameter lead to large drops in detectable protein. This has historically made deep and reproducible proteome profiling of very small cells considerably more challenging.

**Fig. 8:**
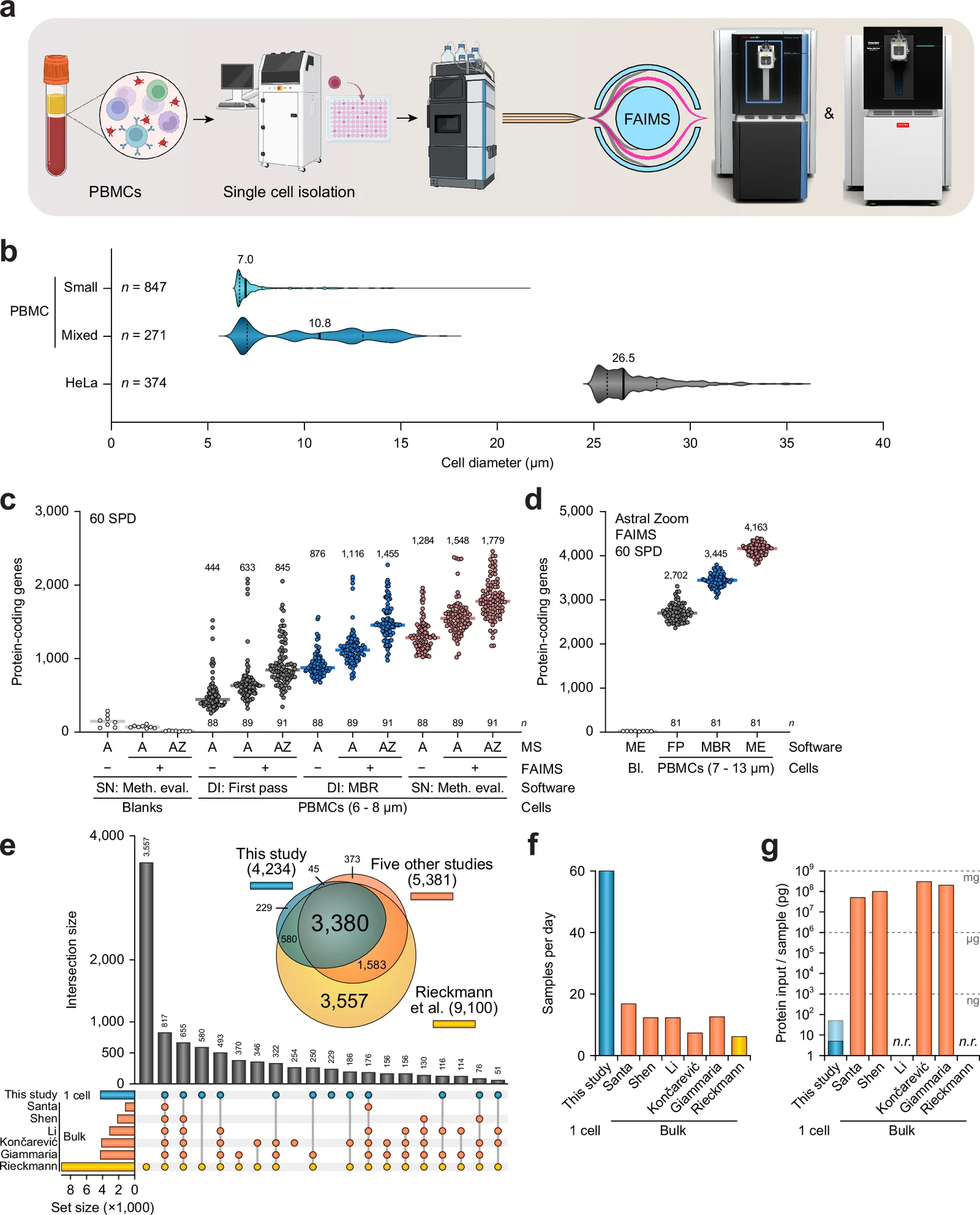
Profiling single peripheral blood mononuclear cells with proteome depth comparable to bulk analysis. **a** Schematic overview of the experimental design. Peripheral blood mononuclear cells (PBMCs) were isolated from buffy coats derived from anonymous donor blood, and single PBMCs were sorted into and digested in 96-well plates using the CellenOne platform, and digests were directly analyzed using a Vanquish HPLC, laser-pulled and in-house packed analytical columns, and an Orbitrap Astral Zoom mass spectrometer prototype. Partially created in BioRender. Olsen, J. (2026) https://BioRender.com/ygoith1. **b** Size distribution profile of all PBMCs isolated in this study. PBMCs were prepared to exclusively yield the smallest cells (“Small”), or minimally processed to gain a mixed population (“Mixed”). HeLa cells are shown for reference (see Figs. 4–5). Thick lines represent median, with median values indicated, and thin dashed lines indicate 1st and 3rd quartiles. *n* = number of sorted cells as indicated. **c** Number of protein-coding genes identified from the smallest PBMCs (median diameter ~7 μm), comparing performance of an Orbitrap Astral (“A”) with and without FAIMS, to an Orbitrap Astral Zoom (“AZ”) with FAIMS (400 − 800 m/z, 20 Th, 40 ms). Bars represent median values, exact medians displayed above each group of data. Data analysis using DIA-NN (“DI”) or Spectronaut (“SN”) as indicated. “MBR”; DIA-NN matching-between-runs, “dDIA”; Spectronaut directDIA. *n* = 88-91 single PBMCs. **d** As **c**, but visualizing analysis of the Mixed PBMC population using the Orbitrap Astral Zoom with FAIMS (400 − 800 m/z, 20 Th, 40 ms). *n* = 81 single PBMCs. ME Spectronaut with Method Evaluation, FP DIA-NN with first pass, MBR DIA-NN with matching-between-runs. **e** UpSet plot analysis, visualizing distinct and overlapping sets of proteins identified in this study from single PBMCs, to proteins identified by published bulk PBMC studies using a wide range of different technical approaches^28–33^. To ensure stringency, proteins identified from single PBMCs in this study were only considered if identified by both DIA-NN and Spectronaut. Proteins from other studies were taken as reported by the respective authors, with data filtering and alignment steps described in the methods section. Exact values are displayed above each bar. The in-set scaled Venn diagram depicts global overlap between proteins identified from single PBMCs in this study, the summation of five intermediate-sized bulk PBMC studies (“Five other studies”), and proteins reported by an in-depth and comprehensive bulk PBMC profiling by Rieckmann et al. **f** As **e**, indicating the sample throughput of the methodology used in each study. **g** As **e**, visualizing the amount of starting material in picograms per sample, for each study. Lower numbers indicate reduced material requirement, and are thus favorable. “n.r”; not reported.

PBMCs are commonly investigated in clinical practice^24,25^, because they represent a minimally invasive and accessible window into systemic immune activity. The PBMC population consists primarily of lymphocytes (T cells, B cells, and natural killer cells)^26^, and the majority are very small cells (6–8 µm in diameter)^27^. Most contemporary proteomics studies of PBMCs still rely on bulk analysis, where billions of cells are co-analyzed, and cell-to-cell heterogeneity is lost^28,29^. To evaluate our optimized SCP workflow in the context of very small primary immune cells, we applied it to PBMCs derived from human blood (Fig. 8a). To visualize the difference in size between PBMCs and human epithelial cancer cells, e.g., HeLa and HEK293T cells, we plotted the size distribution of all single cells isolated in this study (Fig. 8b and Supplementary Fig. 11A). HEK293T and HeLa cells are generally in the range of 20–30 µm, whereas a mixed population of PBMCs contained cells in the range of 6–16 µm, with the smallest 6–8 µm cells being the most numerous, corresponding to ~50 times less protein per cell (Supplementary Fig. 11B).

First, we performed an investigation of the smallest PBMCs, where we processed the PBMCs to exclude larger cells prior to CellenOne isolation and digestion (Fig. 8b). In this experiment, we compared data acquisition using the Orbitrap Astral without FAIMS, the Orbitrap Astral with FAIMS, and the Orbitrap Astral Zoom with FAIMS (Fig. 8c and Supplementary Fig. 11C). Generally, we were able to identify ~4000 to ~6500 precursors per single PBMC, and ~900 to ~1800 proteins per single PBMC. In all cases, FAIMS notably enhanced identifications at both the precursor and protein levels (+21% to +43%), consistent with what we observed in our dilution series experiments at ultra-low input amounts (Fig. 3a, b and Supplementary Fig. 6A, B). Furthermore, using the Orbitrap Astral Zoom MS, we could profile more proteins (+15% to +33%), similarly in line with what we found previously (Fig. 3d, e and Supplementary Fig. 6C, D). Next, we investigated the mixed population of PBMCs that were minimally processed prior to CellenOne isolation and digestion, with all data acquisition performed using the Orbitrap Astral Zoom with FAIMS (Fig. 8d and Supplementary Fig. 11D). From this experiment, we could identify ~19,000 to ~26,000 precursors and ~3400 and ~4200 proteins from single PBMCs, respectively, with DIA-NN and Spectronaut.

To validate our single PBMC data, we performed a comprehensive comparison against bulk PBMC proteomics studies^28–33^, which currently represent the vast majority of MS-based PBMC investigation. To this end, we overlapped proteins identified from single PBMCs in this study with six published bulk PBMC proteomics studies that utilized a wide range of experimental designs and LC-MS instrumentation, and performed both Venn diagram comparison and UpSet plot analysis to highlight overlapping sets of proteins across all studies (Fig. 8e, Supplementary Fig. 11E, and Supplementary Data 3). Notably, despite using single-cell inputs, the depth of proteome coverage achieved here approached or exceeded that reported in several bulk PBMC proteomics studies. An exception was the human hematopoietic cell atlas study by Rieckmann et al., which mapped significantly more proteins through in-depth profiling of 28 primary human hematopoietic cell populations in steady and activated states^30^. Otherwise, the second-largest intersection represented 817 proteins identified across all seven studies, and generally we observed a strong overlap between proteins identified in this work from single PBMCs versus proteins identified from bulk PBMC Fig. 8e and Supplementary Fig. 11E). Importantly, we analyzed our samples at considerably higher throughput (Fig. 8f), and used seven orders of magnitude (~10 million) times less input material compared to bulk PBMC studies (Fig. 8g), demonstrating the sensitivity achievable with our optimized workflow.

**Fig. 9:**
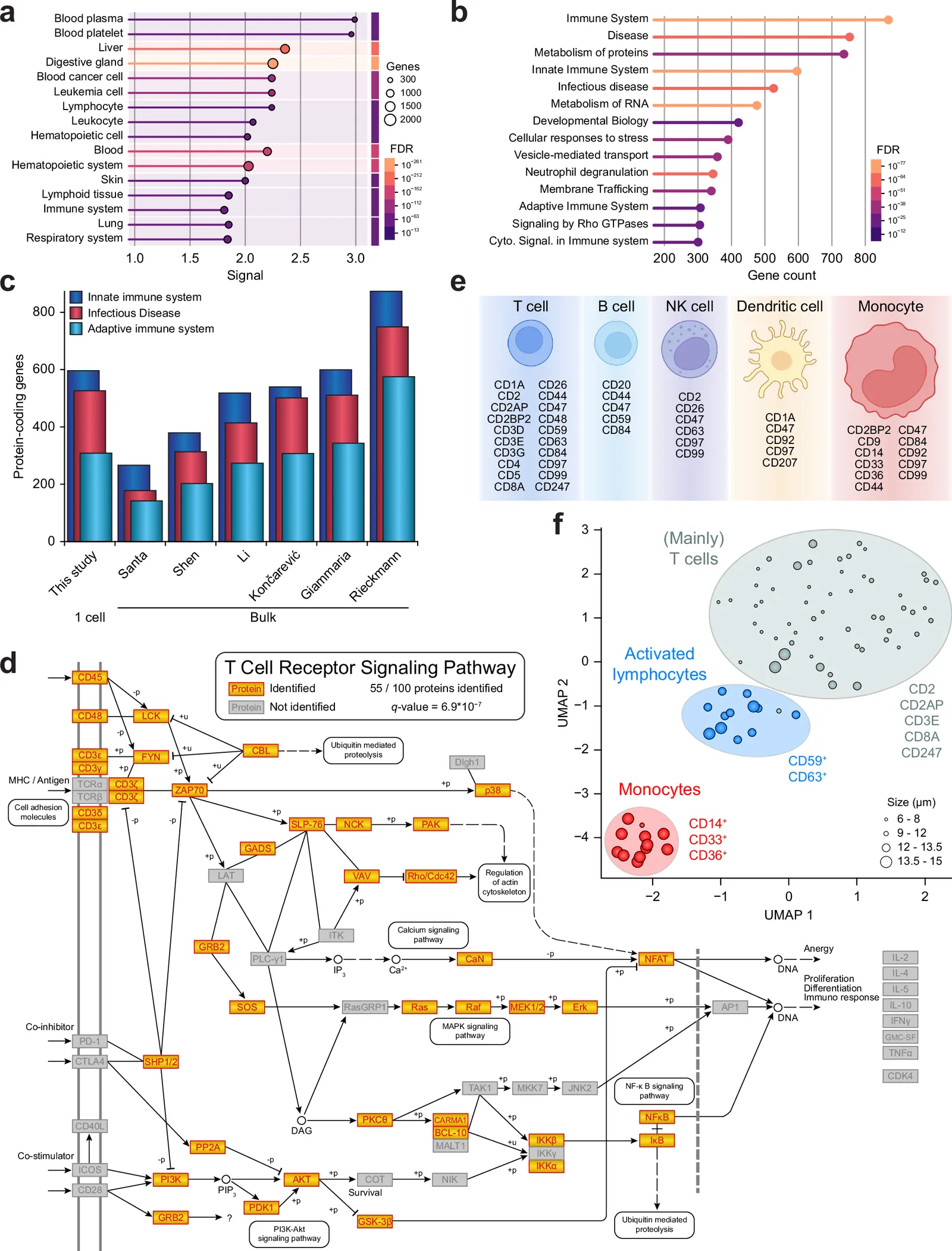
Insights into distinct PBMC cell populations at the single-cell level. **a** TISSUES term enrichment analysis on all protein-coding genes identified from single PBMCs in this study (see Fig. 8), querying for TISSUES annotation via the STRING database^50^. “Signal” is an admixture of both term enrichment and FDR, as reported by the STRING database. The top 10 clusters of overrepresented terms are shown; for the full list of enriched terms, refer to Supplementary Data 4. FDR and protein-coding gene count for each term are indicated. **b** As **a**, but for Reactome Pathway enrichment. The largest enriched categories are visualized, for the full list of enriched terms, refer to Supplementary Data 4. FDR for each term is indicated. **c** As **b**, but visualizing the number of protein-coding genes corresponding to the indicated immune-related Reactome categories, as identified in this study from single PBMCs, or from bulk PBMC in six other studies^28–33^. **d** Kyoto Encyclopedia of Genes and Genomes (KEGG) pathway analysis^48^, mapping all protein-coding genes identified in this study from single PBMCs to the “T Cell Receptor Signaling Pathway” as curated by KEGG. Overall protein identification number and the accompanying FDR value were derived through the STRING database (Supplementary Data 4). **e** Overview of all Cluster of Differentiation (CD) and associated proteins identified from single PBMCs, grouped by the class of PBMC these markers are usually observed in, based on Rieckmann et al.^30^. Partially created in BioRender. Olsen, J. (2026) https://BioRender.com/ygoith1. **f** Euclidian UMAP analysis of the Mixed PBMC population, using all CD proteins identified by DIA-NN and Spectronaut to drive the clustering. Orthogonal unsupervised hierarchical Pearson clustering was used to trace the two most distinct cellular clusters of CD markers (Supplementary Fig. 12C), which are colored red and blue, with the overrepresented CD proteins indicated in the same colors. Remaining cells are colored in gray, with various small clusters of heterologously observed CD markers indicated. Sizes of all individual cells, as recorded by the CellenOne during sorting, are indicated. *n* = 81 single PBMCs.

From a biological perspective, when considering the tissue expression database TISSUES localization annotation^34^, the proteins we identified from single PBMCs were functionally overrepresented for blood-related terms, including blood plasma, lymphocyte, leukocyte, and the immune system (Fig. 9a). When considering Reactome annotation^35^, the largest clusters of proteins we identified from single PBMCs were associated with the immune system, infectious disease, and the cellular response to stress (Fig. 9b). In relation to bulk PBMC studies^28–33^, we found as many or more proteins related to the innate immune system, adaptive immune system, and infectious disease (Fig. 9c), and in similar distributions. Although we did not profile as many immune-related proteins as Rieckmann et al., our data showed a more significant statistical overrepresentation (Supplementary Fig. 12A). Within the adaptive immune response, we noted significant coverage over five out of eight pathways annotated by Reactome (Supplementary Fig. 12B), including T-cell regulation and signaling, B cell signaling, and MHC Class I and II antigen presentation. Among overrepresented KEGG pathways, one notable example also included the T cell receptor signaling pathway, with a significant coverage over ~55% of proteins involved in the pathway (Fig. 9d), in line with ~61% coverage over the same pathway in Reactome (Supplementary Fig. 12B).

As PBMCs represent a heterogeneous mixture of different cell types, we wondered whether we could distinguish between cell types exclusively based on our single PBMC proteomics data, without any pre-sorting or other orthogonal techniques to provide cellular identity. To this end, we investigated all cluster of differentiation (CD) proteins present in our single PBMC data, as these proteins strongly correlate with cellular identity and are routinely used distinguish between cell types^36^. Across our PBMC experiments, we could reliably detect 26 CD proteins (Fig. 9e), which we assigned to the five main classes of PBMC based on the quantitative data from Rieckmann et al. We observed most markers corresponding to T cells, followed by monocytes, although in general CD marker proteins are not always exclusive to one single cell type. Finally, utilizing only the CD proteins identified from our single PBMC analysis, we performed a Euclidean UMAP clustering analysis (Fig. 9f). We additionally performed unsupervised hierarchical Pearson clustering to find overrepresented clusters of CD proteins (Supplementary Fig. 12C), through which the two most notable clusters were overrepresented in either CD14/33/36 or CD59/63. When superimposing these clusters onto the UMAP, along with cellular sizes as determined during single-cell sorting, we found two distinct clusters of cells corresponding to monocytes and activated lymphocytes (Fig. 9f), highlighting the strength of our strategy by demonstrating that we are able to distinguish distinct cell types from a mixed population without prior knowledge.

Using our optimized SCP workflow, we achieved deep label-free proteome profiling of individual PBMCs, including very small lymphocytes with substantially (~50-fold) lower protein content than HeLa cells. Despite the limited cellular material, our workflow enabled broad coverage of immune-associated proteins and pathways, while maintaining relatively high analytical throughput. Furthermore, the proteomic data resolved distinct immune cell populations directly from heterogeneous PBMC samples without prior cellular labeling or sorting. Together, these findings demonstrate the applicability of our optimized workflow to challenging primary immune cell populations and support its utility for high-resolution profiling of immune heterogeneity from patient-derived samples.

## Discussion

In this study, we systematically optimized and integrated each component of the SCP workflow, from peptide recovery and chromatographic design to data acquisition and instrument configuration. While several individual aspects of low-input and SCP methodology have previously been explored independently^3–5,11–13^, our goal here was to benchmark and integrate these advances into a unified ultra-sensitive label-free SCP workflow. Our investigation of chromatography and surfactant-assisted recovery establishes a cohesive low-input workflow that improves both depth and throughput from low-picogram samples. We find that presence of DDM in the lowest amount we tested (~1000-fold weight excess over peptide) was sufficient to increase identification rates from low-input samples, with higher amounts of DDM providing no further benefit. Indeed, DDM is routinely applied in the context of SCP to assist in peptide recovery and cell lysis^15^. Although concentrations of 0.1% DDM are typically used in master mix, we did not observe a difference in protein recovery efficiency when decreasing the concentration to 0.01% DDM. This observation suggests that lower concentrations of DDM may already be sufficient to support efficient sample processing under our experimental conditions. On the chromatography side, we utilized 50 µm ID columns at an effective flow rate of 125 nl/min during peptide elution, essentially aiming to minimize flow rate while maintaining an adequate column backpressure. It has previously been established that lower flow rates can increase identification rates^37^, due to more efficient ionization and reduced background interference^14^.

Balancing depth and throughput is a persistent challenge in SCP, whereas longer gradients can increase the number of proteins identified per cell; such an approach also restricts daily sample throughput. Considering that cell-to-cell variance can be extensive, especially in heterogeneous cell populations, typically dozens to hundreds of single cells need to be acquired in order to be able to draw statistically significant conclusions. In this work, we optimized an analytical gradient using the Vanquish Neo UHPLC, which conditions, loads, and initiates the gradient at high pressure (>1000 bar), before gradually reducing flow rate and pressure to a low level for peptide elution. This concomitantly increases effective peptide elution time across the gradient to ~90%, while still gaining full benefit from efficient ionization. A caveat of using a setup with sufficiently high column backpressure during low flow rates is that loading of the sample onto the analytical column is relatively time-consuming, which makes it impractical to inject volumes larger than a few microliters. In the HPLC context, using a pre-column setup^14^, or potentially a dual-column configuration, could minimize overhead. Another approach to increase sample throughput is to label samples and perform multiplexed analysis^13,38^, however, multiplexing strategies are typically expensive, laborious, and subject to ratio compression depending on how conditions and channels are combined within the same multiplex^39^. Overall, we acquired the majority of our SCP data at 40 or 60 SPD, which is on par with contemporary methodology^3,11^, and sufficient for most standard SCP experiments.

We directly compared the Orbitrap Astral MS with the Orbitrap Astral Zoom MS for analysis of low-input samples, and found that the Orbitrap Astral Zoom’s LIM, pre-accumulation features, and enhanced spectral processing conferred measurable increases in precursor and protein identifications (Fig. 2b). These empirical gains align with observations from recent Orbitrap Astral Zoom studies performed in higher-input and high-throughput proteomics settings^17,40^, while extending evaluation of the platform into ultra-low-input and single-cell-relevant acquisition regimes.

In the context of SCP or low-input proteomics, some studies rely on FAIMS^11,38^, whereas others acquire data without FAIMS^3,41^. In this study, we designed SCP methodology that works both without and with FAIMS, and achieved similar results from 100-250 pg HeLa lysate, as well as from single HeLa cells. To achieve this without FAIMS, we utilized ultra-sensitive nDIA wherein the precursor scan range is restricted to a width of 200 m/z, allowing the mass spectrometer to spend more time accumulating for each DIA window while keeping the cycle time constant at ~1 sec. It should be noted that this comes at a cost in terms of peptides identified and overall sequence coverage, which may impact peptide-centric applications such as characterization of post-translational modifications. Nevertheless, we find that the number of proteins identified increased, and moreover the quantitative precision was improved for ultra-sensitive nDIA. Further improvements could potentially be made through exclusion of known polysiloxane peaks from the DIA windows, or through overlapping DIA windows to further increase the number of datapoints per peak.

Application of FAIMS provided a clear advantage at ultra-low loads ( < 50 pg) by removing singly charged background and enriching for multiply charged peptide ions, yielding up to twofold increases in protein identifications from HeLa lysate in the low-picogram range, and up to 40% more proteins from single PBMCs. FAIMS has previously been shown to increase protein identifications at high input amounts, albeit at a cost of peptide identifications^42^. In the context of low input proteomics, others have also observed that FAIMS increases identifications^38^. Although FAIMS efficiently removes singly charged contamination, it comes at a modest loss of overall transmission, and we speculate that the cross-over point where acquisition with FAIMS becomes superior likely correlates with both the level of atmospheric contamination within the lab as well as the amount and purity of the samples analyzed. In this context, ongoing efforts towards increasing the transmission of FAIMS could likely expand the range of samples and applications where it would be beneficial to utilize FAIMS^43^. Based on these observations, we summarize practical recommendations for selecting the appropriate acquisition strategy depending on sample amount and analytical objective (Table 1).

**Table 1:** Recommended LC-MS acquisition strategies for different sample input levels

Intended application	Approximate input range	Single cells	Recommended workflow	Primary objective	Notes
Conventional DIA proteomics	>1 ng	–	Broad scan range (e.g., 300–1000 or 380–980 Th), 2–4 Th, 2.5–5 ms	Maximum peptide coverage	Highest peptide coverage; preferred for peptide-centric applications (e.g., PTM analysis) when sample amount is not limiting.
Low-input DIA proteomics	50 pg–1 ng	Larger cells (> 15 µm), e.g. HeLa	425–625 Th, 4 Th, 17.5 ms	Maximum protein depth	Best balance between proteome depth and quantitative precision for low-input samples.
Ultra-low-input DIA proteomics	<50 pg	Smaller cells (< 15 µm), e.g. PBMCs	400–800 Th + FAIMS, 20 Th, 40 ms	Maximum analytical sensitivity	Highest sensitivity below ~50 pg by suppressing singly charged background. Reduced peptide coverage could make this workflow less suitable for peptide-centric applications.

Recommendations are based on the workflows evaluated in this study and are intended as general guidance. Optimal acquisition parameters may vary depending on LC configuration, chromatographic performance, sample complexity, and the mass spectrometer used.

Recent SCP studies investigating HeLa and related epithelial cancer cell models have increasingly emphasized the balance between proteome depth and analytical throughput^3,11^. In this study, our optimized workflow achieved the deepest label-free SCP coverage reported to date while maintaining comparatively high throughput across multiple LC-MS regimes (Fig. 5). Notably, we found that substantial proteome depth could still be retained at throughput exceeding 100 SPD, both for low-input HeLa lysate and single-cell analysis. In the context of single HeLa cells, direct injection workflows maintained proteome coverage approaching ~7000 proteins even at throughput exceeding 100 SPD (Fig. 6).

We further evaluated trap-and-elute chromatography configurations, which are increasingly incorporated into both high-throughput and low-input proteomics workflows^4,14,17,22^. While trap-and-elute enabled throughput up to 240 SPD, we consistently observed reduced proteome depth compared to direct injection under matched gradient conditions, particularly in the context of single-cell analysis. Similar reductions in identifications upon transition from direct injection to trap-and-elute workflows have also been reported previously in low-input proteomics^14^, although other recent studies have demonstrated strong overall robustness and flexibility of trap-column configurations for low-input LC-MS workflows^22^. Importantly, even under the highest-throughput conditions evaluated here (240 SPD), we were still able to identify ~3500 proteins from single HeLa cells. Together, these findings suggest that the optimal balance between throughput, loading strategy, and proteome depth remains highly context-dependent and likely varies according to sample amount, chromatographic configuration, and analytical priorities.

Applying our SCP pipeline to PBMCs, we were able to quantify up to 4000 proteins per single PBMC (7–13 µm), observed strong enrichment for immune system and infectious disease pathways, and detected a panel of CD markers sufficient to segregate monocytes and activated lymphocytes by unsupervised clustering. While previous studies have demonstrated SCP analysis of hematopoietic and immune-related cell populations^5,12,13^, these approaches frequently relied on booster-proteome carrier-based multiplexing strategies, longer acquisition times, or achieved lower proteome depth from very small cells. Importantly, our measurements were obtained using a fully label-free workflow without reliance on experimental or computational carrier channels to boost identifications. Strikingly, our single PBMC proteomic results rivaled large-scale bulk PBMC studies^28–33^, while requiring seven orders of magnitude less input material. Of note, the bulk studies had a range of different biomedical aims and relied on a range of different hardware, making it difficult to directly compare. Further, the comprehensive study by Rieckmann et al.^30^ identified considerably more proteins overall, but used ~3 weeks of MS acquisition time. Importantly, as we analyzed single PBMCs, our capability to resolve immune cell identity and pathway coverage provides a direct, label-free molecular alternative to antibody-based cytometry and imaging approaches, and agrees with the notion that proteomics can now directly tackle clinically relevant primary samples.

Taken together, iterative refinement of LC-MS parameters, incorporation of FAIMS, and implementation of the Orbitrap Astral Zoom mass spectrometer enabled ultra-deep profiling of HeLa cells, surpassing 7000 proteins per cell. Furthermore, we achieved deep proteome coverage of PBMCs, identifying ~4000 proteins per single PBMC and revealing cell type-specific signatures directly from mixed populations. These results demonstrate that a carefully optimized SCP workflow can achieve both great depth and throughput without the need for carrier proteomes or labeling strategies. Collectively, this work establishes a robust, accessible platform for SCP that bridges the gap between discovery-scale analysis and clinically relevant applications, opening new avenues for exploring cellular heterogeneity and molecular mechanisms in health and disease.

## Methods

### Sample collection

All research involving human-derived samples was conducted in accordance with relevant ethical regulations and the principles of the Declaration of Helsinki. Peripheral blood samples were obtained as surplus material from anonymous healthy adult blood donors at the Capital Region Blood Bank, Copenhagen University Hospital–Rigshospitalet, following written informed consent. The anonymized sample collection was approved by the local ethics committee (Reference: F-24061507).

### Cell lines and cell culture

HeLa cells (CCL-2, female) and HEK 293 T cells (CRL-3216, female) were acquired via the American Type Culture Collection. All cells were cultured at 37 °C and 5% CO2 in Dulbecco’s Modified Eagle’s Medium (Invitrogen) supplemented with 10% fetal bovine serum and a penicillin/streptomycin mixture (100 U/mL; Gibco). All cells were routinely tested for mycoplasma. Cells were not routinely authenticated.

### PBMCs preparation

Fresh buffy coats were derived from human peripheral blood from anonymous donors, and were acquired from Rigshospitalet, Copenhagen. While these buffy coats were partially enriched for leukocytes, they still contained some red cells and other blood components. PBMCs were isolated from the buffy coats using Lymphoprep™ (STEMCELL Technologies) density gradient medium, essentially according to the manufacturer’s instructions. Briefly, 15 mL Lymphoprep was added to Leucosep 50 mL tubes (Greiner), and centrifuged for 1 min at 1000 *g* at room temperature. Buffy coats were diluted 1.25× using phosphate-buffered saline, and ~25 mL of diluted buffy coat was poured into the Leucosep tubes (up to the 40 mL line). The tubes were centrifuged at 1000 × *g* for 10 min at room temperature, in a swing-out centrifuge with delayed acceleration and deceleration. Mononuclear cells were harvested by pouring the liquid standing on top of the filter into clean 50 mL polypropylene tubes, after which PBS was added to fill up the tubes. Cells were pelleted via centrifugation at 500 × *g* for 5 min at room temperature, and the pellet was washed once more in the same manner. Purified PBMCs were resuspended in a mixture of 90% fetal bovine serum and 10% DMSO, prior to being slowly frozen to −80 °C and stored for up to several months until further handling. To recover PBMCs, they were slowly thawed on ice, after which they were washed once with PBS and pelleted at 1000 × *g* to recover a mixed population of PBMCs. We note that the above procedure results in PBMCs we refer to as “minimally processed” or “Mixed” population. Alternatively, PBMCs were washed several times with PBS and centrifuged at 500 × *g*, removing the larger cells that had pelleted, and keeping the supernatant containing only the smallest PBMCs; referred to as the “Small” population.

### Bulk cell lysis and protein digestion

Cells were washed twice with ice-cold PBS, and gently scraped at 4 °C in a minimal volume of PBS. Cells were pelleted by centrifugation at 500 × *g*, and vigorously lysed in of SDS Lysis Buffer (1% SDS, 100 mM Tris-HCl, pH 8.5). Lysates were heated to 99 °C for 10 min while shaking vigorously (1400 RPM) in a ThermoMixer, after which lysates were cooled to 30 °C and centrifuged for 2 min at 14,000 × *g*. Reduction and alkylation of protein disulfide bonds was performed via concomitant addition of tris(2-carboxyethyl)phosphine (TCEP) and 2-chloroacetamide (CAA) to final concentrations of 10 mM, and incubation at 30 °C for 30 min. On-bead proteolytic digestion was performed using the protein aggregation capture (PAC) protocol^44^. Briefly, proteins were aggregated from the SDS lysate via addition of 10 volumes of acetonitrile (ACN). Magnetic beads (MagReSyn® Hydroxyl) were added to the mixture in a 1:2 protein-to-bead ratio, and samples were mixed three times by gentle inversion, waiting for 1 min in between each mixing. Beads were allowed to settle by gravity for 2 min, prior to being immobilized via a magnetic rack. Immobilized beads were washed twice with 100% ACN, and once with 70% ethanol, after which all liquid was removed, and beads were allowed to air-dry for 10 min. Beads were resuspended in 50 mM HEPES pH 8.5, and proteins were digested on-beads via addition of Lys-C (Wako) at a 1:200 enzyme-to-protein (w/w) ratio for 1 h at 37 °C, and subsequently via addition of modified sequencing-grade trypsin (Promega) at a 1:100 (w/w) ratio, overnight at 37 °C. Following digestion, beads were immobilized on the magnet, and digests were clarified through 0.45 µm spin filters prior to peptide clean-up using C18 StageTip.

### StageTip purification of peptides

Bulk HeLa digest peptides were purified using C18 StageTips at low pH. To this end, C18 StageTips were prepared in-house, by layering four plugs of C18 material (Sigma-Aldrich, Empore SPE Disks, C18, 47 mm) per StageTip. Activation of StageTips was performed with 100 μL 100% methanol, followed by equilibration using 100 μL 80% ACN in 0.1% formic acid (FA), and two washes with 100 μL 0.1% FA. Samples were acidified via addition of TFA to a final concentration of 0.5%. Samples were centrifuged for 5 min at 14,000 × *g*, after which supernatants were loaded onto StageTips. Subsequently, StageTips were washed twice using 100 μL 0.1% FA, after which peptides were eluted using 80 µL 30% ACN in 0.1% formic acid. All samples were dried to completion using a SpeedVac at 60 °C, dissolved to a final concentration of 25 ng/µL in 0.1% FA, and stored at −20 °C. For LC-MS analysis, peptides were diluted 200-fold in 0.1% FA to 125 pg/µL, optionally containing DDM. For the majority of experiments, DDM was added to a final concentration of 0.005% (50 ng/µL). HeLa experiments were performed using 125 pg (1 µL) or 250 pg (2 µL), and dilution experiments were done via serial dilution, ensuring that either 0.5, 1, or 2 µL were injected, in all cases resulting in injection of 25–100 ng of DDM per run.

### Single-cell isolation and digestion

Cultured cells were washed twice with PBS, and detached from the dish using Trypsin-EDTA (Gibco), which was quenched with a small volume of cell culture medium. Cells were pelleted by centrifugation in a swing-out centrifuge at 500 × *g* for 2 min, and washed twice with PBS. Cells were diluted to ~200 cells/μL using PBS, and kept on ice until single-cell sorting. PBMCs were prepared as outlined above, and also diluted to ~200 cells/µL using PBS and kept on ice until sorting. A CellenOne (Cellenion) was used to dispense ~500 nL of ice-cold master mix into each well of LoBind 96-well plates (Eppendorf), using a PDC M Piezo Dispensing Capillary (Cellenion) which has an average drop volume of ~375 pL. Mastermix contained 30 mM triethylammonium bicarbonate (TEAB) pH 8.5, 0.05% DDM, 10 ng/µL Trypsin Gold (Promega; Mass Spectrometry Grade), 5 ng/µL Lys-C (Wako), and 250 µM DTT (Proteomics Grade). This master mix was used for the majority of the experiments; any deviations are indicated in figure legends. Mastermix was always kept on ice, mixed well and centrifuged for 2 min at 14,000 × *g* just prior to drawing and dispensing. After dispensing of mastermix, the PDC was flushed twice, and used to draw cells. The CellenOne was used to characterize properties of the cell populations, and used to dispense single HeLa (25–35 µm), single HEK (20–35 µm), or single PBMC (6–15 µm) cells directly into the 96-well plate containing mastermix. For most experiments, several wells were configured to receive 20 cells to serve as a ‘carrier’. For specific experiments, either 2, 5, 40, or 60 cells, were pooled into some wells, which is indicated in the figure legends. After sorting, another 250 nL of mastermix was dispensed into all wells. Subsequently, digestion was performed using the CellenOne, at 50 °C for 2 hours, during which the PDC continuously dispensed small volumes of distilled water into each of the wells to both cause a mixing effect within the wells and offset the evaporation occurring at 50 °C. After digestion, the CellenOne was used to cool down the plate to 20 °C for 20 min, during which dispensing of distilled water continued. At all stages of CellenOne sample preparation and depending on atmospheric conditions, the system automatically controlled relative air humidity to prevent either drying out of the wells or condensation of liquid within the system. This ensured a final volume of ~4 µL in each well, and following the digestion procedure, the plates were briefly centrifuged at 250 × *g* to ensure all samples were similarly located at the bottom of the wells. Reactions were quenched and stabilized via addition of 1 µL of 1.5% formic acid, after which plates were stored at 4 °C for up to 2 weeks until MS analysis. Just prior to transferring the plates to the HPLC, plates were centrifuged for 2 min at 250 × *g*. In the HPLC, plates were kept at 7 °C.

### LC setup

The vast majority of samples were analyzed on 20-cm long analytical LC columns with an ID of 50 µm, laser-pulled and packed in-house using ReproSil-Pur 120 C18-AQ 1.9 µm beads (Dr. Maisch), with all column setups defined in Supplementary Data 1. As an alternative to our in-house manufactured columns (see Supplementary Note 1), Reprosil-Pur 120 C18-AQ 1.9 µm 200 ×0.050 mm NanoFusion columns (Part Number: R119.AQ.PT200.050.04) were provided by Dr. Maisch. Online reversed-phase LC to separate peptides was performed using a Vanquish™ Neo UHPLC System (Thermo Scientific) in direct injection mode unless otherwise specified. For trap-and-elute mode, a PepMap Neo C18 5 µm 300 µm × 5 mm pre-column (Thermo Scientific) was used. The analytical LC column was heated to 40 °C using a column oven (Sonation). Peptides were eluted from the column using a gradient of Buffer A (0.1% formic acid) and Buffer B (80% ACN in 0.1% FA). As optimization of the alytical gradient is a main focus in this study, a large number of gradients were used and are described in Supplementary Data 1. Expected sample throughput per day (SPD) is indicated in figure legends. Pre- and post-run column equilibration was set to 0.5 column volumes, additional volume of buffer following sample load was set to 1 µL, and all loading and equilibration were performed at 1200 bar. For the majority of the 60 SPD experiments, the gradient was started at 500 nL/min (~1000 bar) with 10% B. Linearly over 1 min, flow rate was reduced to 125 nL/min while increasing to 23% B, resulting in a stabilized pressure of ~250 bar after 2 min and synchronized with the start of peptide elution. Subsequently, over 4.75 min to 31% B, over 2.25 min to 39% B, and over 1 min to 47% B, which signified the end of the recorded gradient due to a column delay of ~6 min at 125 nL/min flow. Next, over 4.75 min to 95% B, flow rate was linearly increased to 500 nL/min over 0.25 min, and kept there for 1 min. The last ~6 min of the LC gradient volume eventually passed through and washed the column, but was not recorded by the MS.

### Mass spectrometric analysis

All samples were analyzed using an Orbitrap Astral mass spectrometer, an Orbitrap Astral Zoom mass spectrometer prototype, or an Orbitrap Astral Zoom mass spectrometer. Electrospray ionization (ESI) was achieved using a Thermo Scientific^TM^ Nanospray Flex^TM^ Ion Source. Spray voltage was set to 2 kV, capillary temperature to 275 °C, and RF level to 50%, unless otherwise indicated. As optimization of MS settings is a main focus in this study, a large number of different configurations were used across all experiments, and all the settings are detailed in Supplementary Data 1. Here, we describe the settings used for the majority of SCP experiments, which were acquired either without (“no-FAIMS”) or with (“FAIMS”) the use of a Thermo Scientific^TM^ FAIMS Pro Duo Interface. We note that “no-FAIMS” always refers to the physical absence of the FAIMS unit, rather than just disabling the device. All full precursor (MS1) scans were acquired using the Orbitrap™ mass analyzer, while all tandem fragment (MS2 / DIA) scans were acquired in parallel using the Astral™ mass analyzer. Orbitrap lock mass correction was performed at run start using EASY-IC™, for no-FAIMS runs only. For FAIMS, the compensation voltage (CV) was set to −46 V for all scans. Expected peak width was set to 5 s. Full scan range was set to 420–630 m/z (no-FAIMS) or 400–800 m/z (FAIMS), MS1 resolution to 240,000, MS1 AGC target to “500” (5,000,000 charges), and MS1 maximum injection time to 25 ms. DIA scans were executed across a scan range of 425–625 m/z (no-FAIMS) or 400–800 m/z (FAIMS), using 4 m/z (no-FAIMS) or 20 m/z (FAIMS) isolation windows, DIA maximum injection time of 17.5 ms (no-FAIMS) or 40 ms (FAIMS), and fragmentation was performed using HCD with normalized collision energy of 25. DIA fragment scan range was set to 150–1500 m/z, DIA AGC target to “200” (20,000 charges). Loop control was set to “All”, resulting in a full MS1 & DIA cycle time of ~1 sec. For acquisition using the Orbitrap Astral Zoom mass spectrometer prototype, pre-accumulation was enabled via the method editor, and HAR, enhanced spectral processing (ESP), and LIM were manually enabled via the Service Diagnostic Tool using a custom license. Low-input mode was emulated on the prototype instrument by increasing the PMT voltage by 75 V ( ~ 13%), unless otherwise indicated.

### MS data analysis, general

All MS data files were analyzed with DIA-NN v1.9.2^45^ or Spectronaut v19 (Biognosys). Considering the vast amount of MS data and different experiments, multiple separate computational searches were performed, depending on experimental design. Search engines and search modes are indicated in the figures, figure legends, or otherwise described in Supplementary Data 1. Here, we describe the global configuration used for the large majority of all data searches. For generation of theoretical spectral libraries, a complete HUMAN.fasta reference proteome database including isoforms was downloaded from UniProt on the 13th of November, 2024, containing 105,529 entries. Notably, for both DIA-NN and Spectronaut, inference to protein-coding genes was done via gene names, to avoid inflation of identification rates via isoforms.

### MS data analysis, DIA-NN

For analysis with DIA-NN v1.9.2, RAW files were always converted to DIA files in order to speed up subsequent analysis. For library generation for library-free searching, minimum and maximum peptide length were set to 6 and 60, respectively, and minimum and maximum charge to 2 and 4, respectively. Precursor m/z range and fragment m/z range were set to match the MS acquisition strategy; in case of multiple different ranges in the same search, the library range was defined broad enough to encompass all experiments. Missed tryptic cleavages were set to 1 for HeLa lysate searches, or 2 for single-cell searches. N-terminal methionine excision was allowed for all searches, and cysteine carbamidomethylation was only used for HeLa lysate searches. No variable modifications were considered. For MBR library generation, smart profiling was used for HeLa lysate searches and full profiling was used for single-cell searches. The quantification strategy was set to “Legacy”. Default settings were used otherwise, including protein-group inference via unique gene name and FDR control set at 1%.

### MS data analysis, spectronaut

For analysis with Spectronaut v19, default settings were used, with exceptions and notable settings outlined below. For the initial Pulsar search, maximum variable modifications per peptide were set to 3, allowing protein N-terminal acetylation, deamidation of glutamine and asparagine, and methionine oxidation. N-terminal methionine excision was allowed (default). Cysteine carbamidomethylation was not used as a fixed modification, as we did not alkylate single-cell samples. For phosphorylation searches, serine/threonine/tyrosine phosphorylation was defined as a variable modification with up to 3 modifications per peptide. Missed tryptic cleavages were set at 2 (default), and minimum and maximum peptide length were set to 6 and 60, respectively. Fragment ion m/z range was set to match the MS acquisition strategy. Relative intensity threshold for fragment ions was disabled for searching, including non-FAIMS data. Searches were either performed in “Method evaluation” or standard directDIA mode, as indicated in the figure legends. In general, method evaluation was used when combining vastly different datasets in the same computational search. Protein quantification mode was set to “MaxLFQ”, with quantification performed at the MS2 level unless otherwise specified. Major (protein) grouping was performed by gene name (Gene Id), and minor (peptide) grouping was performed by stripped sequence. Phosphorylation localization was required to be >0.75. An FDR of 1% was applied at the PSM, peptide, and protein levels.

### Comparison to other MS studies

For comparison of proteins identified from single HeLa, HEK293T, and H460 cells, publicly available MS data were downloaded from two recent single-cell proteomics studies^3,11^. Specifically, HeLa single-cell data were acquired from Ye et al., and HEK293T and H460 data were acquired from Bubis et al. For comparison using Spectronaut, each dataset from each study (i.e., each separate data column in the figure) was set as a separate condition for Method Evaluation analysis using otherwise the same settings as described above. For comparison using DIA-NN, datasets from each study were searched separately using MBR mode, and afterwards combined into one graph. Peptides per protein and data points per peak were derived from Spectronaut analysis. For depth of sequencing analysis, protein copy numbers (via iBAQ) were derived from a previously published ultra-deep proteome^23^, and proteins identified from single cells were aligned to the deep proteome primarily through Uniprot ID, and otherwise through gene name. For comparison of proteins identified from single PBMCs in this study to published bulk PBMC studies, reported data were taken from six other studies^28–33^. In order to align all identified and reported proteins, a scaffold was created based on all unique protein-coding genes as downloaded from UniProt on the 13th of November, 2024, i.e. the same database as used to search all MS data in this work. Alignment was primarily performed using Uniprot identifier, and otherwise, protein-coding gene. Duplicate proteins, or proteins no longer existing on Uniprot, were omitted. For Končarević et al., all reported Uniprot identifiers were aligned. For Rieckmann et al., the vast majority of gene names were aligned, omitting only entries not quantifiable in any of the 43 different conditions, as well as duplicate entries. For Shen et al., all quantified proteins were aligned. For Li et al., since only unfiltered MaxQuant output was available, we performed basic filtering by removing contaminants and reverse database hits, filtering for at least two peptides per protein, proteins identified in <60% of replicates were discarded, as also performed by Li et al. For Santa et al., all reported Uniprot identifiers were aligned. For Giammaria et al., since we could not find a list of proteins, we downloaded the publicly available MS raw data associated with their work, and re-analyzed the data using DIA-NN v1.9.2 with the same settings as used for data generated in this study. From this, we found essentially the same number of proteins as the authors reported, and those proteins were aligned to the scaffold for comparison between studies. Our own single PBMC data was searched with both DIA-NN and Spectronaut, and for the study comparison we only used protein-coding genes identified by both software. For determination of which markers occur in which types of PBMC, we used the in-depth quantitative data from Rieckmann et al.

### Quantification and statistical analysis

Details regarding the statistical analysis can be found in the respective figure legends. All quantitative experiments were performed using at least four replicates to ensure sufficient statistical power. For single-cell analysis, we aimed to analyze at least 10 single-cell replicates for each condition to account for cell-to-cell biological variance in asynchronous cell populations. For some conditions, due to technical issues, we could not measure at least 10 single cells, in which case we drew no statistical conclusions from those datasets. Statistical handling of the data was primarily performed using the freely available Perseus v1.5.5.3 software^46^, and includes calculation of Pearson correlation and linear regression, hierarchical clustering, and dimensionality reduction (UMAP) analysis. Venn diagram overlaps were performed using Venny v2.1 (https://bioinfogp.cnb.csic.es/tools/venny/), and scaled Venn diagrams were rendered using eulerAPE v2 (https://www.eulerdiagrams.com/eulerAPE/v2/). UpSet plots were generated using ChiPlot (https://www.chiplot.online/upset_plot.html). Boxplots were drawn using BoxPlotR (http://shiny.chemgrid.org/boxplotr/). Violin plots were drawn using GraphPad Prism v11.0.1. Term enrichment analyses (Uniprot keywords, Reactome, TISSUES) were performed using the STRING database^47^, v12.0. The “T Cell Receptor Signaling Pathway” was adapted from Kyoto Encyclopedia of Genes and Genomes (KEGG)^48^, v116.0. Reactome Pathway enrichment analysis was performed using and curated by Reactome^35^, v94. Overall, plotting of graphs was done using either Perseus, GraphPad Prism, or Microsoft Excel (Office Professional Plus 2016). The majority of art was drawn by the authors, and all visualizations were polished using Adobe Illustrator (Creative Suite 6). Tube art in Figs. 1a and 2a was adapted from Servier Medical Art (https://smart.servier.com), and the Reactome pathway art in Supplementary Fig. 12B was adapted from Reactome (https://reactome.org/), both licensed under CC BY 4.0 (https://creativecommons.org/licenses/by/4.0/). Certain figure elements were derived from BioRender by Ivo Hendriks and Sara Buch-Larsen via the Olsen lab’s Academic license (2026), detailed in: https://BioRender.com/ygoith1.

### Reporting summary

Further information on research design is available in the Nature Portfolio Reporting Summary linked to this article.

## Supplementary information

Supplementary Information

Description of Additional Supplementary Files

Supplementary Data 1

Supplementary Data 2

Supplementary Data 3

Supplementary Data 4

Reporting Summary

Transparent Peer Review file

## Source data

Source Data

## Data Availability

The mass spectrometry proteomics data generated in this study have been deposited in the ProteomeXchange Consortium via the PRIDE^49^ partner repository, with the dataset identifiers PXD070599 (primary data), PXD078497 (revision 1 data), and PXD080728 (revision 2 data). PXD070599 (primary data). PXD078497 (revision 1 data). PXD080728 (revision 2 data). All other data generated in this study are provided in the Supplementary Information and Source Data files. A comprehensive overview of all LC-MS acquisition parameters for all experiments in this work, as well as which datasets correspond to which figures, is provided in Supplementary Data 1. Source data are provided with this paper.
