# Supplementary material for: Extending single-cell proteomics from HeLa to small primary human immune cells
Source: Nat Commun. 2026 Sep 19;17:10985. doi: 10.1038/s41467-026-77675-x (PMC13683416; doi:10.1038/s41467-026-77675-x)
Supplement: Supplementary file 1 [file 41467_2026_77675_MOESM1_ESM.pdf]

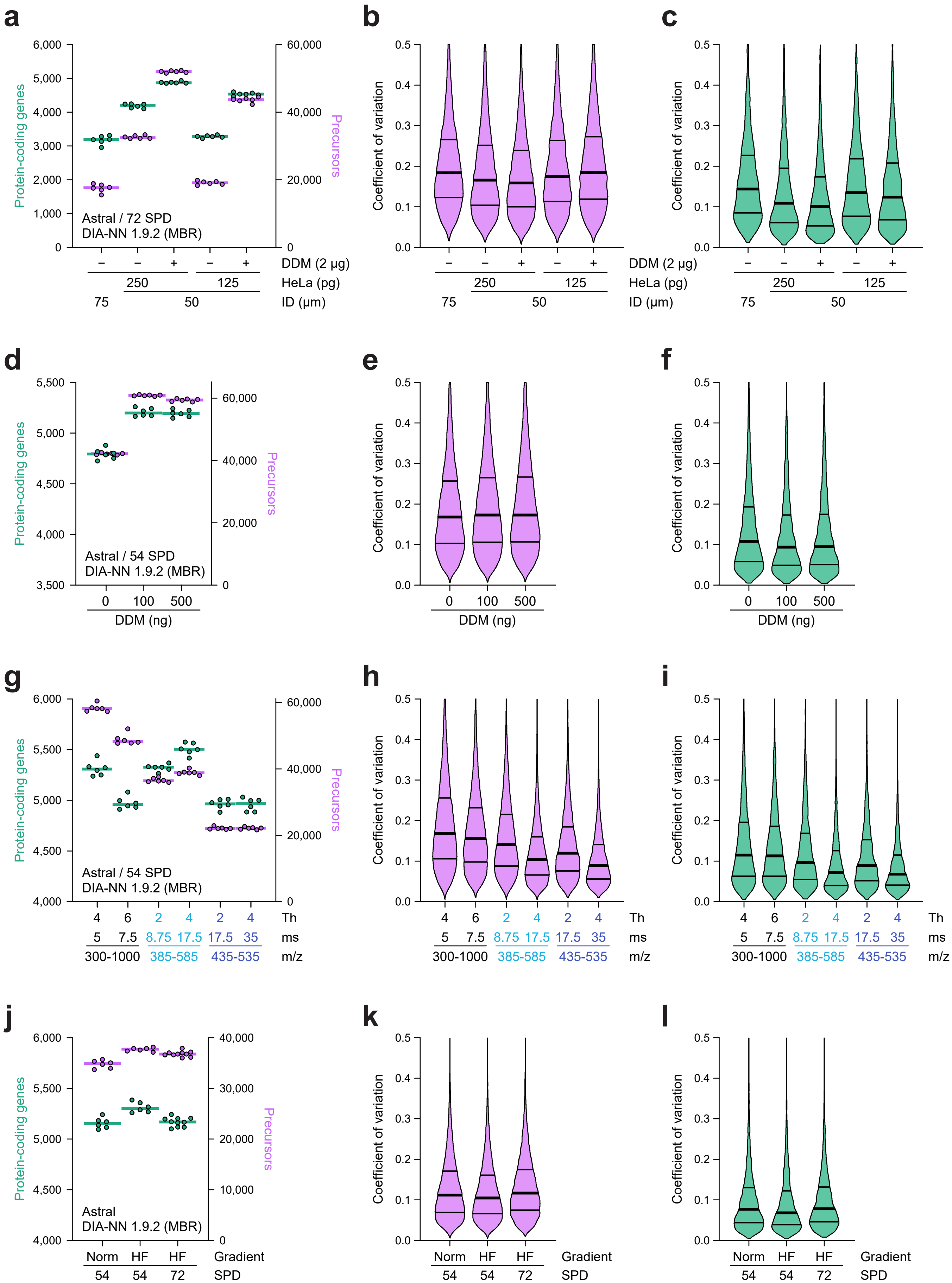

SUPPLEMENTARY FIGURE 1

Supplementary Figure 1. **Optimization of low-input LC-MS methodology for enhanced protein identification rates.** Corresponds to Figure 1. (A) Number of protein-coding genes (green, left axis) and precursors (purple, right axis) identified, when comparing different column inner diameter, HeLa load, and the addition of n-dodecyl β-D-maltoside (DDM). Bars represent median, the numbers above the x-axis represent median coefficient of variation percentage, *n* = 6 technical replicates. "ID"; internal column diameter. Data analysis using DIA-NN v1.9.2 and matching-between-runs enabled. (B) Violin plot distribution of coefficient of variation for all precursors from Figure 1B. MS2-based quantification via DIA-NN. Thick line represents median, thin lines 1st and 3rd quartiles. (C) As **B**, but for protein-coding genes. (D) As **A**, but comparing different amounts of DDM injected. (E) Violin plot distribution of coefficient of variation for all precursors from Figure 1C. MS2-based quantification via DIA-NN. Thick line represents median, thin lines 1st and 3rd quartiles. (F) As **E**, but for protein-coding genes. (G) As **A**, but comparing different ultra-sensitive nDIA methods. "ms"; maximum DIA ion injection time in milliseconds. (H) Violin plot distribution of coefficient of variation for all precursors from Figure 1F. MS2-based quantification via DIA-NN. Thick line represents median, thin lines 1st and 3rd quartiles. (I) As **H**, but for protein-coding genes. (J) As **A**, but evaluating different gradient lengths and throughput speeds. *n* = 6-10 technical replicates. "Norm"; standard steady-flow chromatography, "HF"; method employing Higher Flow at the start and end of the gradient to maximize effective peptide elution time, "SPD"; samples per day. (K) Violin plot distribution of coefficient of variation for all precursors from Figure 1G. MS2-based quantification via DIA-NN. Thick line represents median, thin lines 1st and 3rd quartiles. (L) As **K**, but for protein-coding genes.

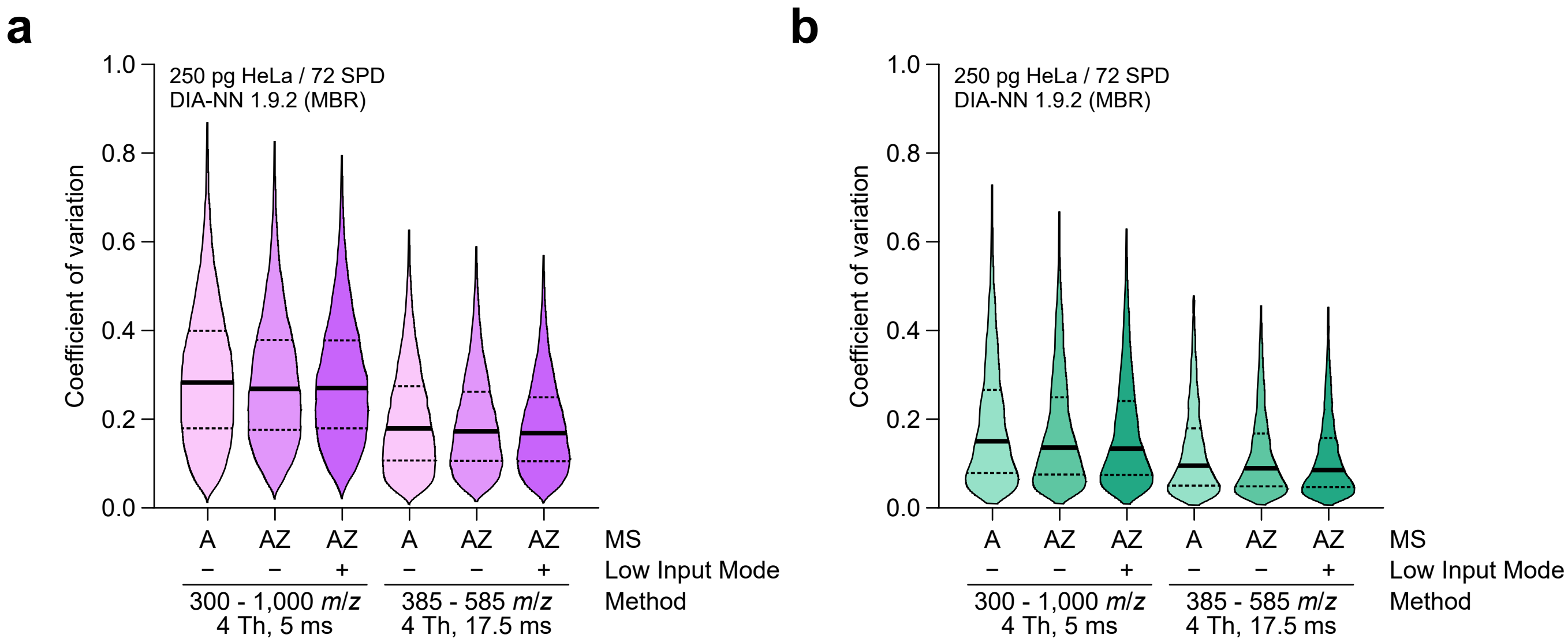

SUPPLEMENTARY FIGURE 2

Supplementary Figure 2. Corresponds to Figure 2B. (A) Violin plot distribution of precursor coefficient of variation observed when comparing Astral ("A") mode with Astral Zoom ("AZ") mode, with the latter either in regular or Low Input mode. Data analysis using DIA-NN v1.9.2 without matching-between-runs, with quantification at the MS/MS level. (B) As **A**, but for protein-coding genes.

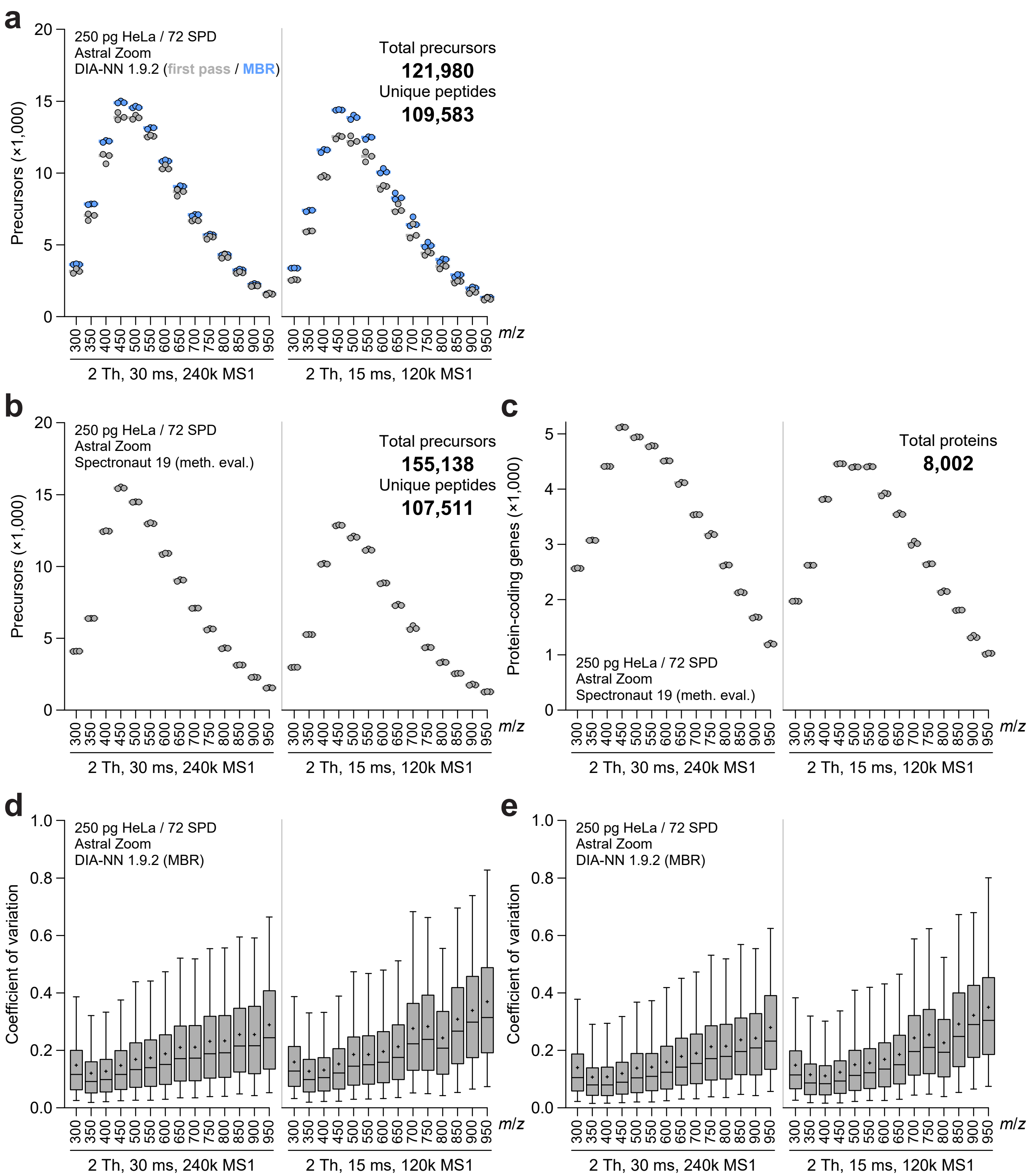

SUPPLEMENTARY FIGURE 3

Supplementary Figure 3. Corresponds to Figure 2C. (A) Gas-phase fractionation experiment, wherein precursors are isolated from a 50 *m/z* range starting at the *m/z* indicated on the x-axis. Gray and blue dots correspond to the number of precursors with data analysis using DIA-NN in first pass mode and MBR mode, respectively. *n* = 3 technical replicates, total number of precursors and unique peptides identified cumulatively across all runs are indicated. (B) As **A**, but data analysis using Spectronaut 19. (C) As **B**, but for protein-coding genes. Total number of protein-coding genes identified cumulatively across all runs is indicated. (D) As **A**, but visualizing coefficient of variation for all identified precursors as a box plot. Central line; median, plus symbol; average, box limits; 1st and 3rd quantile, whisker limits; 5th and 95th percentile. Data analysis using DIA-NN v1.9.2 with matching-between-runs, with quantification at the MS/MS level. (E) As **D**, but for protein-coding gene coefficient of variation.

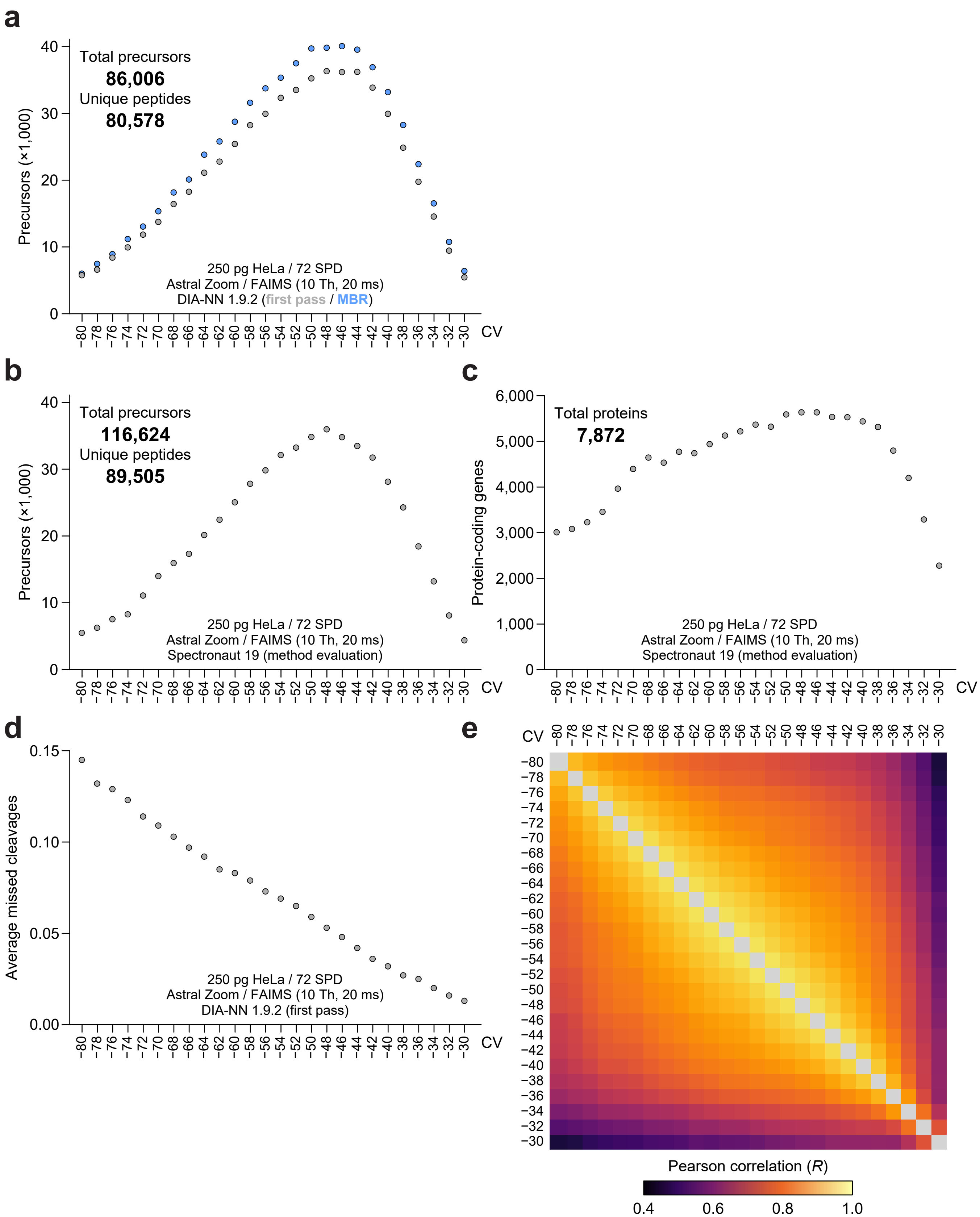

SUPPLEMENTARY FIGURE 4

Supplementary Figure 4. Corresponds to Figure 2D. (A) FAIMS fractionation experiment, wherein precursors were analyzed using a range of FAIMS compensation voltages (CV), using a single constant CV across the entire gradient. Gray and blue dots correspond to the number of identified precursors with data analysis using DIA-NN v1.9.2 in first pass mode and MBR mode, respectively. Scan range was 400 – 800 *m*/*z*. *n* = 1 technical replicate, total number of precursors and unique peptides identified cumulatively across all runs are indicated. (B) As **A**, but data analysis using Spectronaut 19. (C) As **B**, but for protein-coding genes. Total number of protein-coding genes identified cumulatively across all runs is indicated. (D) As **A**, but visualizing the average number of missed tryptic cleavages in all identified peptide sequences. (E) As **A**, showing the Pearson correlation between the abundance of all protein-coding genes identified at each FAIMS CV setting.

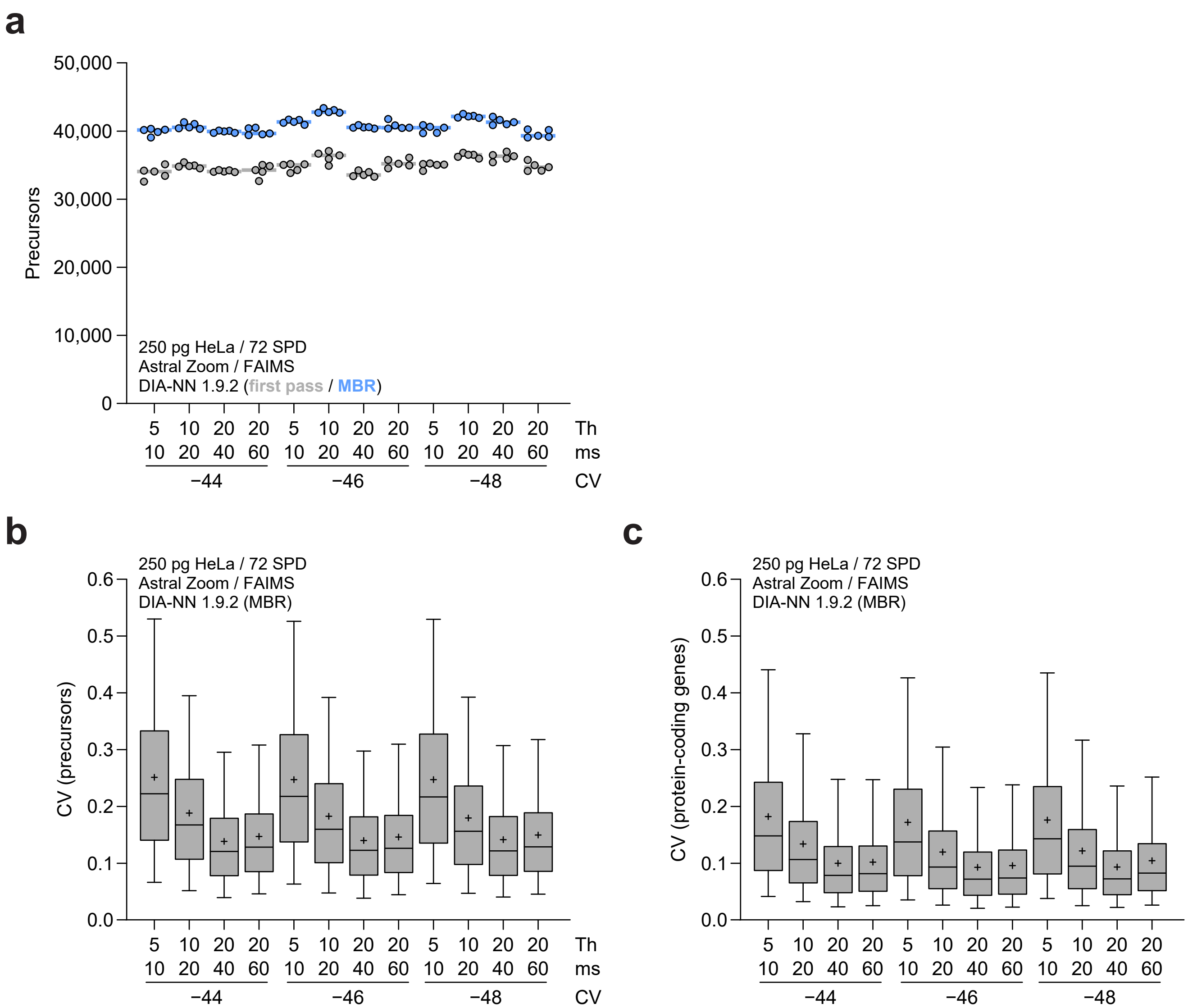

SUPPLEMENTARY FIGURE 5

Supplementary Figure 5. Corresponds to Figure 2E. (A) Number of precursors identified using different combinations of DIA window size in Thomson (“Th”) and maximum allowed DIA ion injection time in milliseconds (“ms”) in combination with three different FAIMS CVs. *n* = 5 technical replicates. Gray and blue dots correspond to data analysis using DIA-NN v1.9.2 in first pass mode and MBR mode, respectively. (B) As **A**, but visualizing coefficient of variation for all identified precursors as a box plot. Central line; median, plus symbol; average, box limits; 1st and 3rd quantile, whisker limits; 5th and 95th percentile. Data analysis using DIA-NN v1.9.2 with matching-between-runs, with quantification at the MS/MS level. (C) As **B**, but for protein-coding gene coefficient of variation.

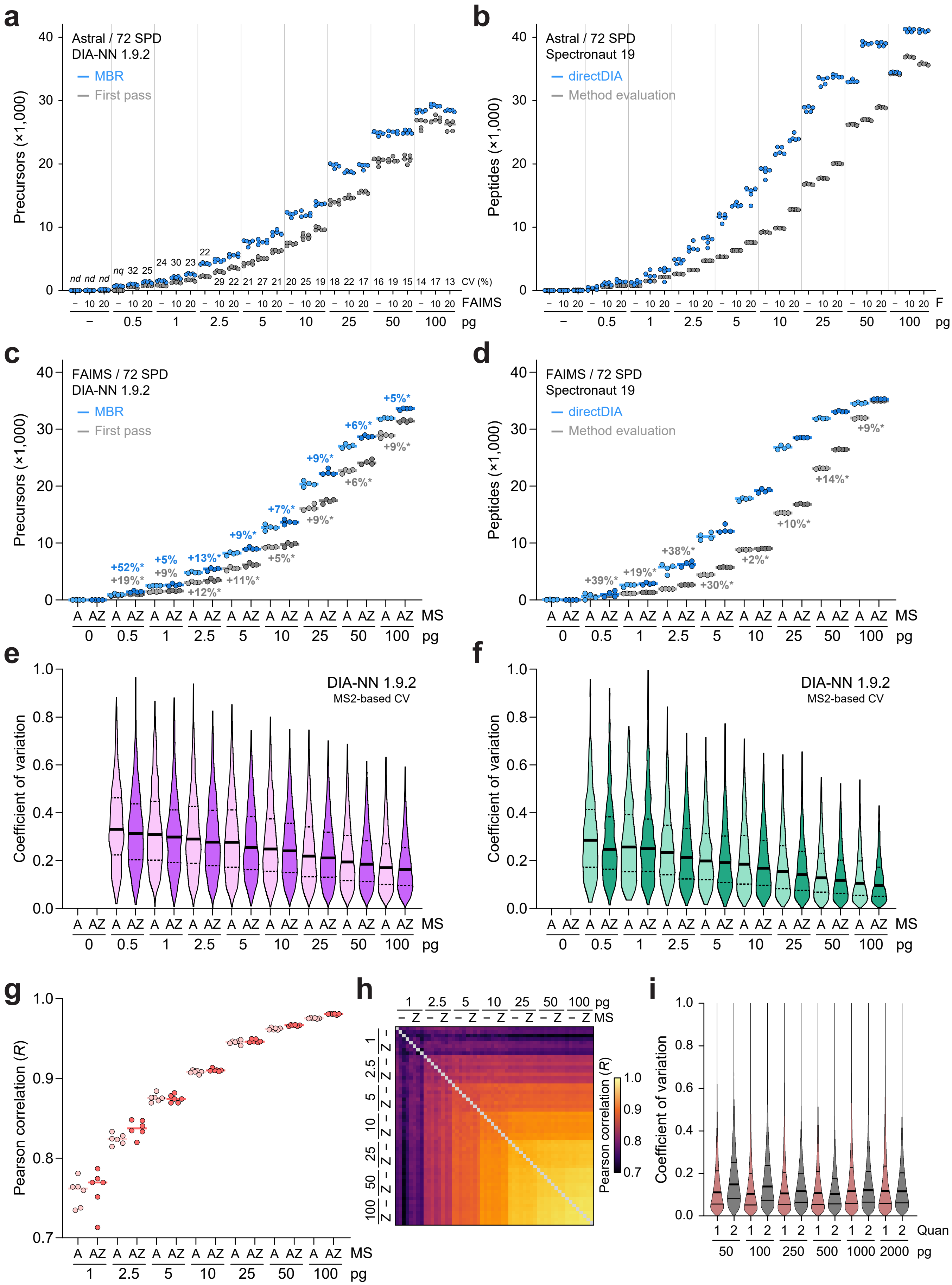

SUPPLEMENTARY FIGURE 6

Supplementary Figure 6. Orbitrap Astral Zoom MS and FAIMS are superior for ultra-low load analysis. Corresponds to Figure 3. (A) Dilution series experiment, ranging from 0.5 pg to 100 pg of injected HeLa lysate, comparing standard analysis to FAIMS analysis. All injections were performed in ascending amounts, starting with a blank to ensure negligible sample carry-over. Number of identified precursors are indicated, bars represent median, with gray and blue corresponding to data analysis using DIA-NN v1.9.2 in first pass mode and MBR mode, respectively. Numbers above the x-axis represent median coefficient of variation percentage, n = 5 technical replicates. "F"; indicator for analysis strategy with "−"; no FAIMS, "10"; with FAIMS using 10 Th window width and 20 ms DIA injection time, "20"; with FAIMS using 20 Th window width and 40 ms DIA injection time. (B) As **A**, but data analysis using Spectronaut 19 and showing number of stripped peptide sequences, with gray and blue corresponding to data analysis using Method Evaluation mode and directDIA mode, respectively. (C) Dilution series experiment as described in **A**, but using FAIMS in all cases and comparing the difference between the Orbitrap Astral ("A") and Orbitrap Astral Zoom ("AZ") using a prototype mass spectrometer. Percentage increases of median values are indicated, * = $p < 0.05$ via two-tailed Student's t-testing, n = 4 technical replicates. (D) As **C**, but data analysis using Spectronaut 19 and showing number of stripped peptide sequences, with gray and blue corresponding to data analysis using Method Evaluation mode and directDIA mode, respectively. Differences of medians were not assessed in directDIA mode due to the non-linear behavior of the directDIA identification strategy. (E) Coefficient of variation for all identified precursors in **C**, as a box plot. Central line; median, plus symbol; average, box limits; 1st and 3rd quantile, whisker limits; 5th and 95th percentile. Data analysis using DIA-NN v1.9.2 without matching-between-runs, with quantification at the MS/MS level. (F) As **E**, but for protein-coding genes. (G) Showing the Pearson correlation between the abundance of all protein-coding genes identified in **F** (and Figure 3D), when considering same-replicate correlation only. n = 4 technical replicates, leading to 6 combinations (data points), bars indicate the median. (H) As **G**, but comparing the abundance of all protein-coding genes between all replicates. (I) Violin plot distribution of coefficient of variation for all precursors from Figure 3G. MS1-based quantification via Spectronaut, MS2-based quantification via DIA-NN. Thick line represents median, thin lines 1st and 3rd quartiles.

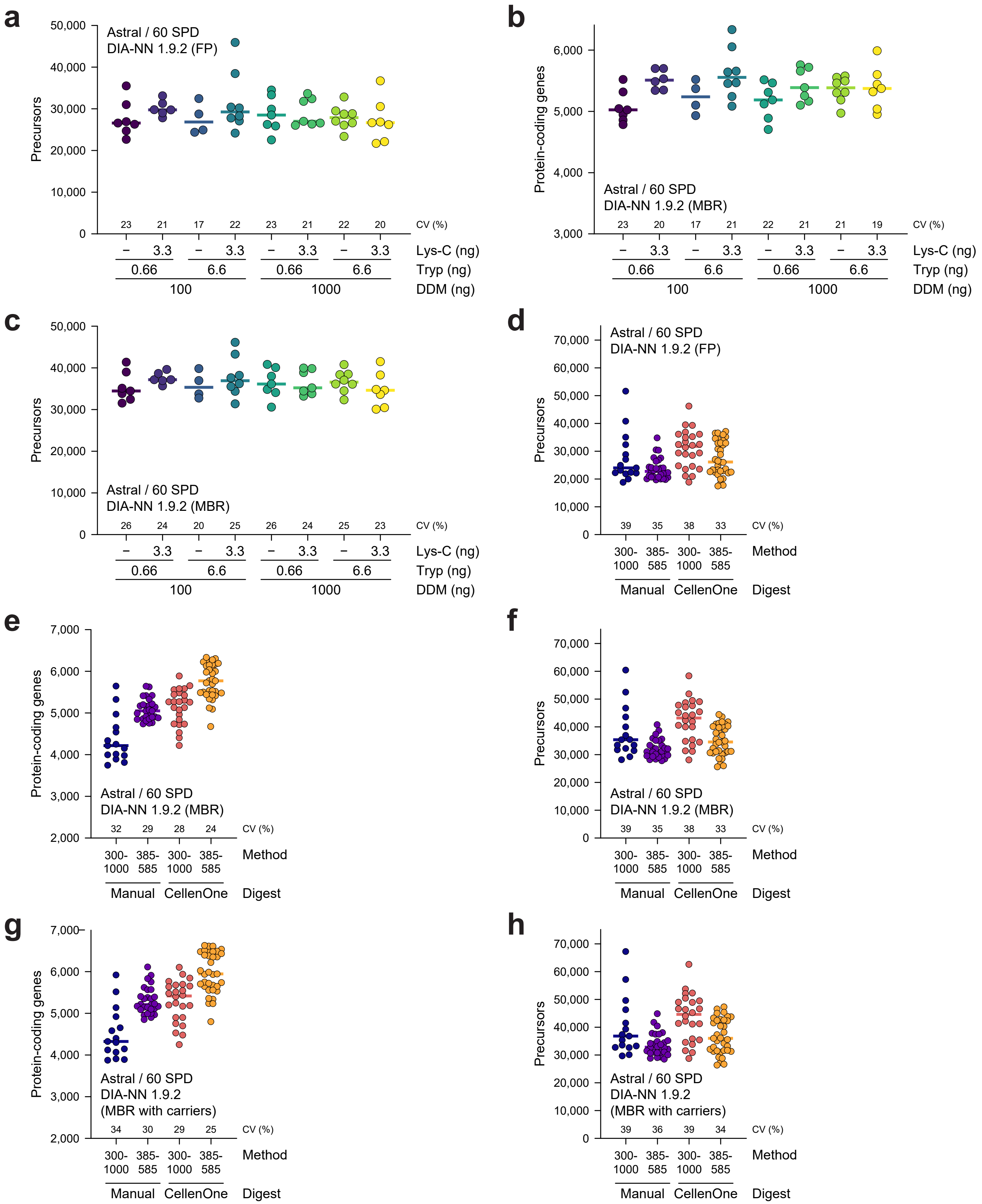

SUPPLEMENTARY FIGURE 7

Supplementary Figure 7. Corresponds to Figure 4B-E. (A) Number of precursors identified from single HeLa cells when using various different digestion mixes. Cells were sorted by the CellenOne platform, but otherwise manually digested. Bars represent median. Numbers above the x-axis are median coefficient of variation. *n* = 4-8 single cells. (B) As **A**, but for protein-coding genes and using matching-between-runs in DIA-NN. (C) As **A**, but using matching-between-runs in DIA-NN. (D) As **A**, but comparing manual digestions (with the CellenOne only used to dispense single cells) to fully automated handling and digestion using the CellenOne, as well as comparing the broad-range nDIA method with ultra-sensitive nDIA. *n* = 15-35 single cells. (E) As **D**, but for protein-coding genes and using matching-between-runs in DIA-NN. (F) As **D**, but using matching-between-runs in DIA-NN. (G) As **E**, but also computationally including 20-cell carriers. (H) As **F**, but also computationally including 20-cell carriers.

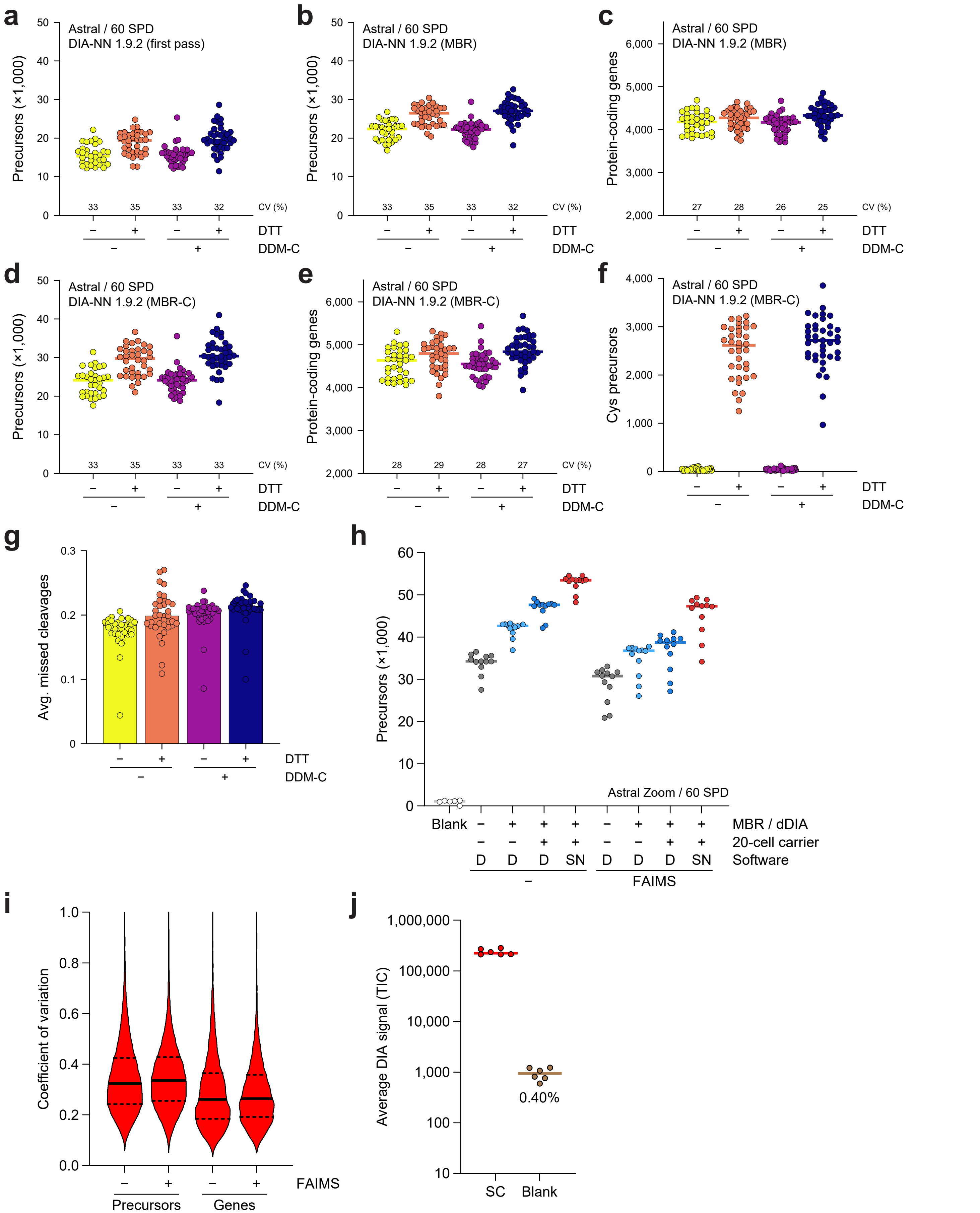

SUPPLEMENTARY FIGURE 8

Supplementary Figure 8. Corresponds to Figure 4F-H. (A) Number of precursors identified from single HeLa cells when comparing the effect of dithiothreitol (DTT) in the digestion mix, as well as pre-coating the 96-well plate with n-dodecyl-ß-D-maltoside (DDM). Cells were sorted by the CellenOne platform, but otherwise manually extracted and digested. Bars represent median. Numbers above the x-axis are median coefficient of variation. *n* = 33-40 single cells. (B) As **A**, but using matching-between-runs in DIA-NN. (C) As **B**, but for protein-coding genes. (D) As **B**, but also computationally including 20-cell carriers ("MBR-C"). (E) As **C**, but also computationally including 20-cell carriers. (F) As **D**, but only showing cysteine-containing precursors. (G) As **A**, but showing the average number of missed tryptic cleavages observed per peptide. (H) Number of precursors (with DIA-NN) or stripped peptide sequences (with Spectronaut) identified from single HeLa cells (median diameter ~26.5 µm) when using optimized sample preparation workflows, computational inclusion of 20-cell carriers, and using either standard- or FAIMS-optimized methodology. Data analysis using either DIA-NN ("D") or Spectronaut ("SN") as indicated. *n* = 12 single cells. "MBR"; DIA-NN matching-between-runs, "dDIA"; Spectronaut directDIA. (I) Violin plot distribution of coefficient of variation for all precursors and protein-coding genes from **H** and Figure 4H. MS2-based quantification via DIA-NN. Thick line represents median, dashed lines 1st and 3rd quartiles. (J) Approximation of system carry-over effect via average DIA ion current signal observed across the entire chromatographic gradient. "SC"; representative single HeLa cell runs. "Blank"; blank injections directly following SC runs with same volume, buffers, and DDM concentration. *n* = 6 single cells and blanks.

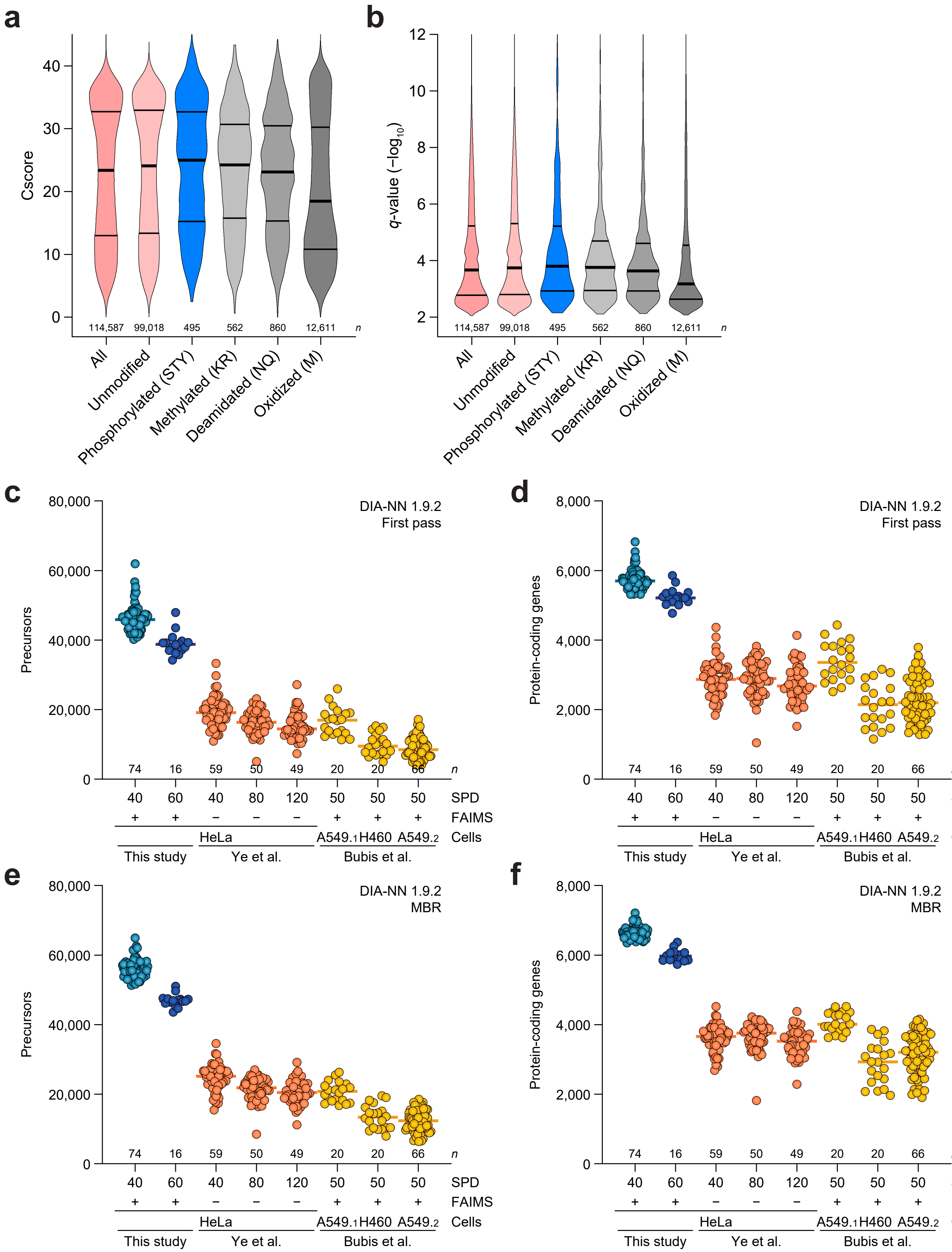

SUPPLEMENTARY FIGURE 9

Supplementary Figure 9. Profiling over 7,000 proteins from single HeLa cells. Corresponds to Figure 5. (A) Violin plot distribution of Spectronaut Cscore values for the indicated classes of unmodified or modified peptides, as identified from *n* = 74 single HeLa cells (median diameter ~26.5 μm) analyzed at 40 SPD, see Figure 5A. Thick line; median, thin lines; 1st and 3rd quartile. Peptide *n* per distribution are indicated above the x-axis. (B) As **A**, but visualizing peptide $-\log_{10}$ *q*-value. (C) Comparison between the number of precursors identified from single cells in this study, to two recent papers using similar single-cell approaches[1,2]. Publicly available MS raw data was downloaded and re-processed using DIA-NN in first pass mode. Bars represent median values. *n* = 16-74 single cells depending on study and batch (see Figure 5C). Appendix numbers following cell type indicate two separate batches of A549 cells from Bubis et al. (D) As **C**, but for protein-coding genes. (E) As **C**, but using matching-between-runs in DIA-NN. To control search space and FDR, each batch of cells was searched separately. (F) As **E**, but for protein-coding genes.

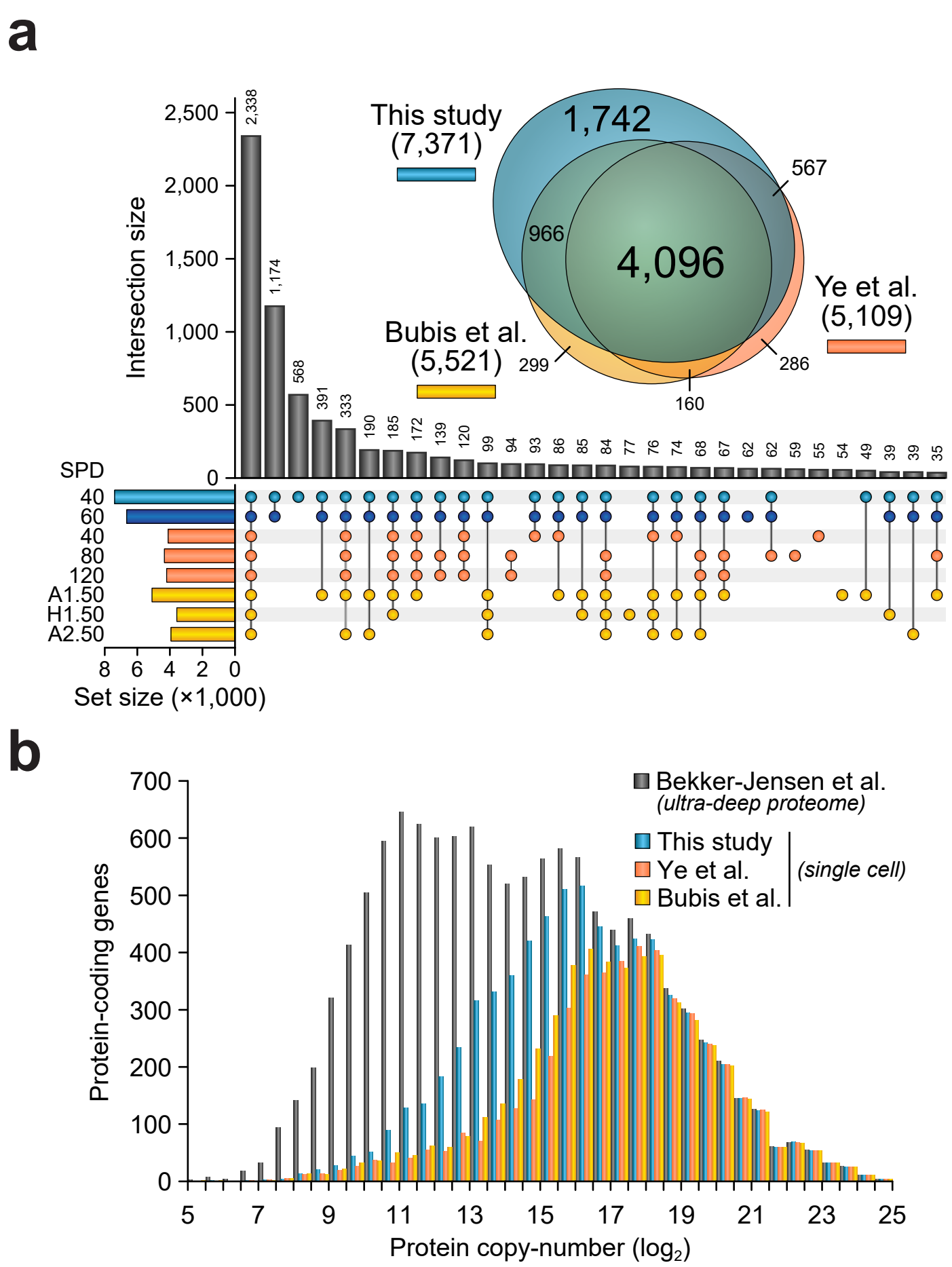

SUPPLEMENTARY FIGURE 10

Supplementary Figure 10. Greatly enhanced single cell proteome depth compared to contemporary single cell studies. Corresponds to Figure 7. (A) UpSet plot analysis, visualizing distinct and overlapping sets of proteins identified from single HeLa cells in this study compared to two recent studies using similar single-cell approaches[1,2], see also Figure 5C-F. Exact values are displayed above each bar. Analysis based on re-processing of available MS data using Spectronaut in Method Evaluation mode, considering all proteins (even those identified by just one peptide) detected in at least 50% of replicates. All single cells were HeLa, except for "A1"; A549 batch 1, "H1"; H460 batch 1, "A1"; A549 batch 2. The in-set scaled Venn diagram depicts global overlap between the three studies. (B) Depth of sequencing visualization, with known protein copy-numbers taken from a published ultra-deep proteome. Proteins identified in all single-cell studies were mapped to the total proteome and binned in 0.5 $\log_2$ bins.

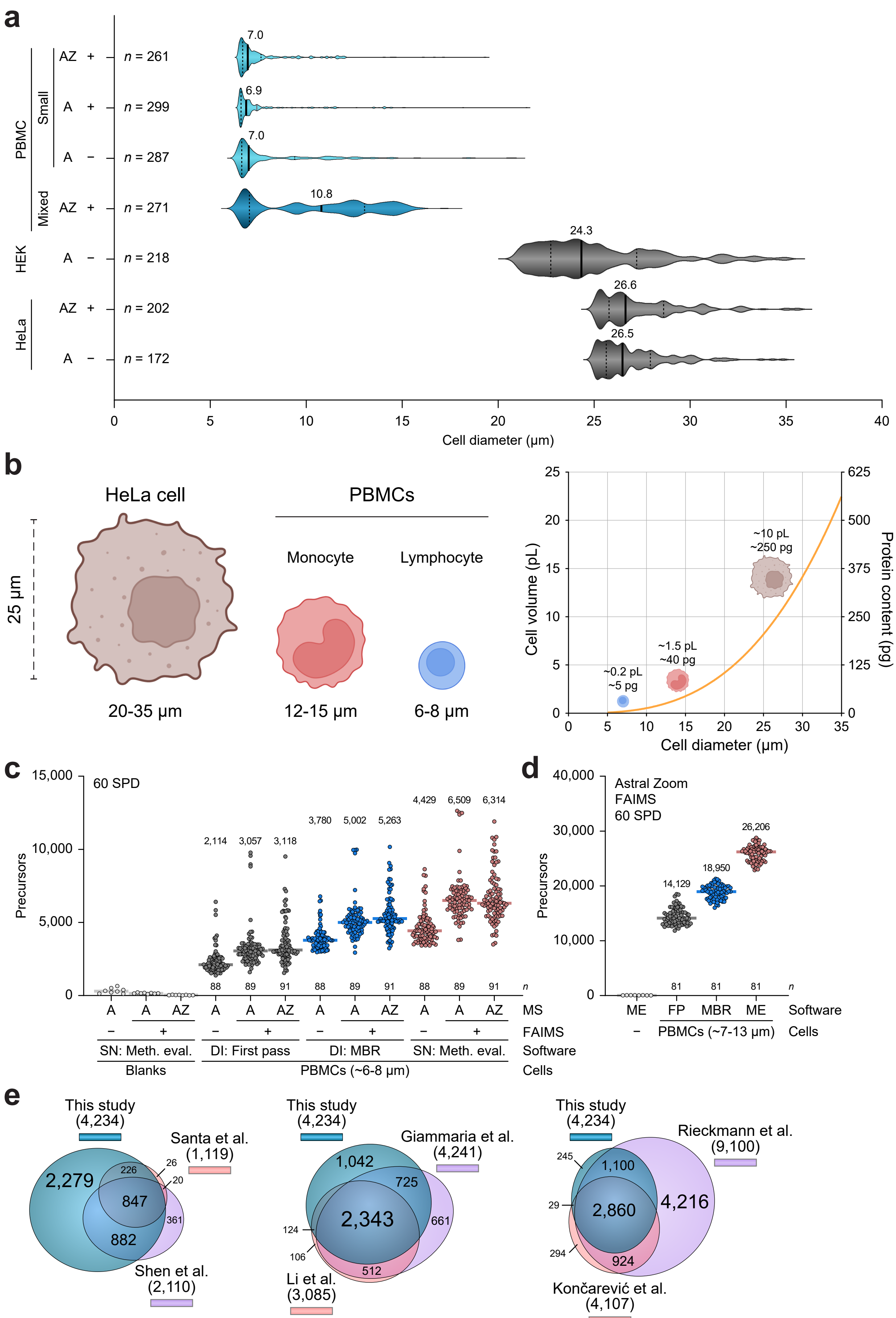
a
PBMC
Small
Mixed
HEK
HeLa
AZ + n = 261
A + n = 299
A − n = 287
AZ + n = 271
A − n = 218
AZ + n = 202
A − n = 172
7.0
6.9
7.0
10.8
24.3
26.6
26.5
Cell diameter (µm)
b
HeLa cell
PBMCs
Monocyte
Lymphocyte
25 µm
20-35 µm
12-15 µm
6-8 µm
~10 pL
~250 pg
~1.5 pL
~40 pg
~0.2 pL
~5 pg
Cell volume (pL)
Protein content (pg)
Cell diameter (µm)
c
60 SPD
Precursors
2,114
3,057
3,118
3,780
5,002
5,263
4,429
6,509
6,314
88 89 91 88 89 91 88 89 91 n
MS
FAIMS
Software
Cells
SN: Meth. eval.
DI: First pass
DI: MBR
SN: Meth. eval.
Blanks
PBMCs (~6-8 µm)
d
Astral Zoom
FAIMS
60 SPD
Precursors
14,129
18,950
26,206
81 81 81 n
ME FP MBR ME
Software
PBMCs (~7-13 µm)
Cells
e
This study (4,234)
Santa et al. (1,119)
Shen et al. (2,110)
2,279
226
26
20
847
361
882
This study (4,234)
Giammaria et al. (4,241)
Li et al. (3,085)
1,042
725
2,343
661
124
106
512
This study (4,234)
Rieckmann et al. (9,100)
Končarević et al. (4,107)
245
1,100
29
2,860
4,216
294
924

Supplementary Figure 11. Profiling single peripheral blood mononuclear cells to a bulk analysis depth. Corresponds to Figure 8. (A) Size distribution profile of all PBMCs isolated in this study, stratified by batch used for each set of MS experiments. PBMCs were prepared to exclusively yield the smallest cells (“Small”), or minimally processed to gain a mixed population (“Mixed”). Size distribution profiles of HeLa cells (Figures 4 and 5) and HEK cells (Supplementary Note 9) are shown for reference. Thick lines represent median, with median values indicated, and thin dashed lines indicate 1st and 3rd quartiles. *n* = number of sorted cells as indicated. “A”; analyzed by Orbitrap Astral, “AZ”, analyzed by Orbitrap Astral Zoom. A plus symbol denotes analysis using FAIMS. (B) Schematic visualization of cell diameter versus cellular volume and protein content. Partially created in BioRender. Olsen, J. (2026) https://BioRender.com/ygoith1. (C) Number of precursors (DIA-NN) or stripped peptide sequences (Spectronaut) identified from the smallest PBMCs (median diameter ~7 µm), comparing performance of an Orbitrap Astral (“A”) with and without FAIMS, to an Orbitrap Astral Zoom (“AZ”) with FAIMS (400 − 800 m/z, 20 Th, 40 ms). Bars represent median values, exact medians displayed above each group of data. Data analysis using DIA-NN (“DI”) or Spectronaut (“SN”) as indicated. “MBR”; DIA-NN matching-between-runs, “dDIA”; Spectronaut directDIA. *n* = 88-91 single PBMCs. (D) As **C**, but visualizing analysis of the Mixed PBMC population using the Orbitrap Astral Zoom with FAIMS (400 − 800 m/z, 20 Th, 40 ms). *n* = 81 single PBMCs. “ME”; Spectronaut with Method Evaluation, “FP”; DIA-NN with first pass, “MBR”; DIA-NN with matching-between-runs. (E) Scaled Venn diagrams depicting overlap between proteins identified from single PBMCs in this study, to six bulk PBMC studies. To ensure stringency, proteins identified from single PBMCs in this study were only considered if identified by both DIA-NN and Spectronaut. Proteins from other studies were taken as reported by the respective authors, with data filtering and alignment steps described in the methods section.

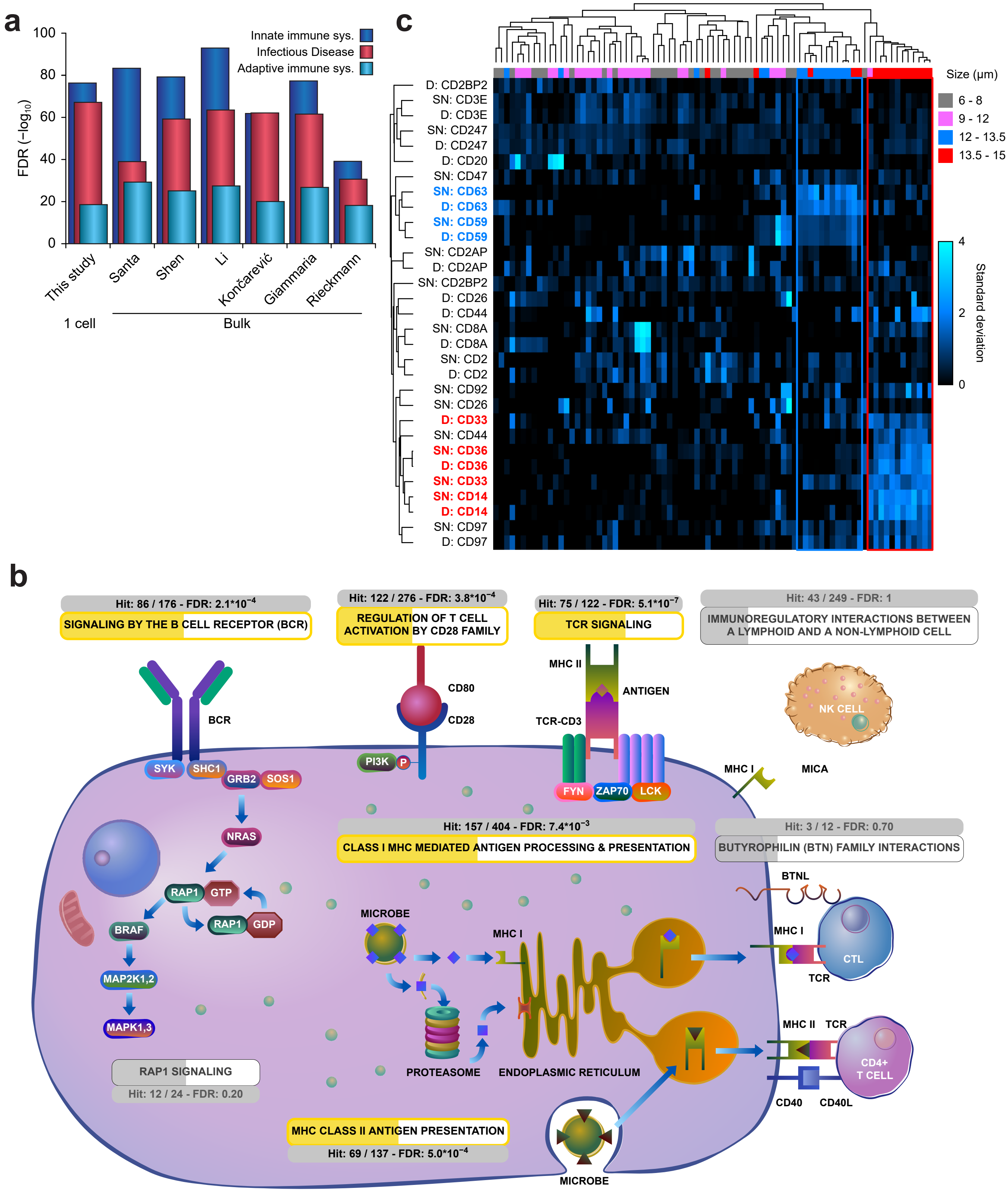

SUPPLEMENTARY FIGURE 12

Supplementary Figure 12. Profiling single peripheral blood mononuclear cells with proteome depth comparable to bulk analysis. Corresponds to Figure 9. (A) Reactome term enrichment analysis for the indicated immune-related Reactome Pathway categories via the STRING database[3], using all protein-coding genes identified from single PBMCs in this study (see Figure **8**), or proteins as reported in six bulk PBMC studies[4,5,6,7,8,9]. The inverse $\log_{10}$ false discovery rate (FDR) corresponding to each category is plotted, with higher values indicating a higher degree of statistical significance. (B) Pathway enrichment analysis performed using and curated by Reactome[10], using all protein-coding genes identified from single PBMCs as input, and visualizing the Adaptive Immune System pathway. The numbers of hits within each sub-pathway are indicated, along with FDR as calculated by Reactome. Significantly over-represented pathways within our single PBMC analysis are highlighted in gold. Art adapted from Reactome (https://reactome.org/), licensed under CC BY 4.0 (https://creativecommons.org/licenses/by/4.0/). (C) Unsupervised hierarchical Pearson clustering of all CD marker proteins identified in the Mixed PBMC population (Figure **8D**). The two most distinct cellular clusters of CD markers are highlighted, boxed and colored in red and blue. Sizes of all individual cells, as recorded by the CellenOne during sorting, are indicated. Cell size was not used to drive clustering. *n* = 81 single PBMCs. "D"; identification and quantification via DIA-NN, "SN"; identification and quantification via Spectronaut.

SUPPLEMENTARY NOTE 1

The analytical columns used throughout this study were laser-pulled fused-silica capillaries packed in-house with ReproSil-Pur 120 C18-AQ 1.9 µm beads. Although this format provides excellent chromatographic performance for ultra-low-input proteomics, preparation of laser-pulled columns is not routinely available in many laboratories. To assess whether comparable performance can be achieved using a commercially manufactured alternative, we directly compared our in-house packed columns with analytical columns from Dr. Maisch (part number R119.AQ.PT200.050.01, alternatively available as R119.AQ.PT200.050.04 in a four-pack). Both columns shared identical dimensions (20 cm × 50 µm ID), incorporated an integrated pulled emitter, and were packed with the same stationary phase (Supplementary Figure 13).

Performance was evaluated using 250 pg HeLa lysate and two complementary DIA acquisition strategies: a conventional broad-range method (350–850 Th, 2 Th isolation windows, 2 ms maximum injection time) and our optimized ultra-sensitive nDIA method (425–625 Th, 4 Th isolation windows, 17.5 ms maximum injection time). Across both acquisition strategies, the commercial and in-house columns exhibited highly comparable performance (Supplementary Figure 13 A-B). In first pass DIA-NN analysis, the numbers of identified precursors and protein-coding genes differed by ~1% between the two column formats, while MBR analysis produced similarly indistinguishable results. Likewise, quantitative precision remained essentially identical, with comparable coefficients of variation observed across all experiments.

To further compare chromatographic performance, we evaluated the median number of MS2 data points acquired across peptide peaks, chromatographic peak widths (FWHM), and the total quantified MS2 ion current (Supplementary Figure 13 C-E). All three metrics showed excellent agreement between the two column formats, indicating equivalent sampling density, chromatographic performance, and ionization efficiency. Collectively, these data demonstrate that the analytical performance described throughout this study is not dependent on in-house column fabrication and can be reproduced using commercially available columns with matching specifications.

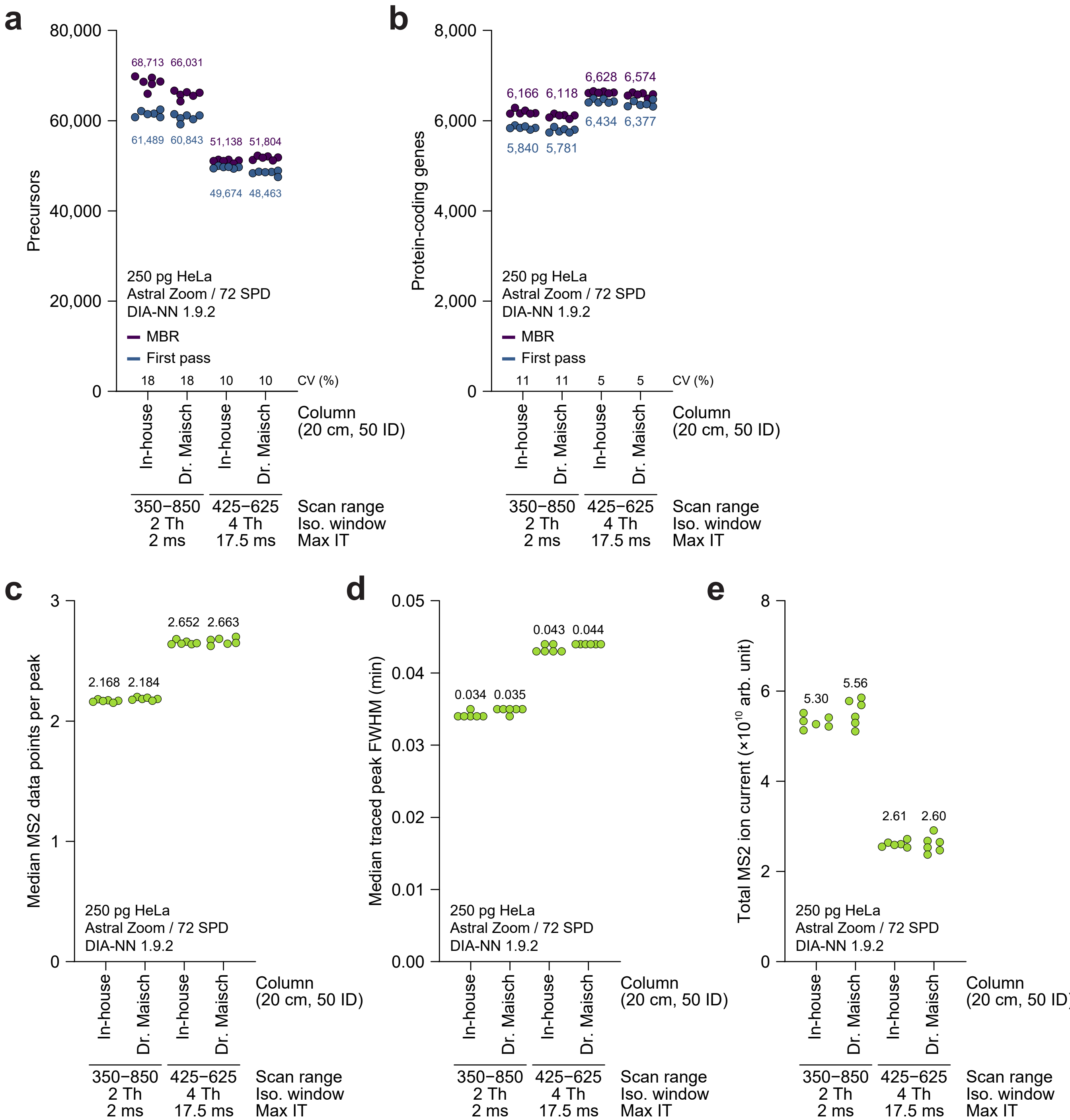

SUPPLEMENTARY FIGURE 13

Supplementary Figure 13. Comparison of an in-house produced column with a commercial column from Dr. Maisch. (A) Number of precursors identified from 250 pg HeLa lysate. All analyses performed using the Orbitrap Astral Zoom MS, and data analyzed using DIA-NN in either first pass or matching-between-runs (MBR) mode. Exact median values are displayed above each group of data for MBR, and below each group of data for first pass. Numbers above the x-axis represent median coefficient of variation percentage, *n* = 6 technical replicates. "Max IT"; maximum injection time, "Iso. window"; isolation window. (B) As **A**, but for protein-coding genes. (C) As **A**, but for median number of MS2 data points per quantified peak. (D) As **A**, but for median traced peak Full Width Half Maximum (FWHM). (E) As **A**, but for the total amount of quantified MS2 ion current per run.

SUPPLEMENTARY NOTE 2

The non-ionic detergent n-dodecyl β-D-maltoside (DDM) is widely used in single-cell proteomics to reduce peptide adsorption to plasticware and chromatographic surfaces, thereby improving peptide recovery from extremely small sample amounts. Throughout this study, we optimized both the concentration and the absolute amount of DDM introduced onto the analytical column, ultimately using approximately 100–500 ng DDM per injection in all optimized workflows (Figure 1C). During method development, however, we also evaluated substantially higher DDM loads to determine whether excessive detergent addition could adversely affect chromatographic performance (Supplementary Figure 14).

When 250 pg HeLa lysate was analyzed together with 2 µg DDM per injection, consecutive LC-MS analyses resulted in progressive accumulation of a broad, late-eluting hydrophobic signal that was absent in control injections without DDM (Supplementary Figure 14A). This observation indicates incomplete removal of DDM and/or DDM-associated hydrophobic material between injections, which may gradually impair chromatographic performance during extended acquisition sequences.

In contrast, injections containing 500 ng DDM together with 250 pg HeLa digest showed no detectable accumulation across six consecutive LC-MS analyses (Supplementary Figure 14B). Although this represents the upper end of the DDM amounts used throughout this study, chromatographic profiles remained highly reproducible, indicating that these detergent loads can be effectively cleared from the analytical system between injections. Collectively, these data demonstrate that relatively small absolute amounts of DDM are sufficient to improve peptide recovery for ultra-low-input proteomics, whereas unnecessarily high detergent loads should be avoided because they may lead to gradual accumulation of hydrophobic material within the LC system.

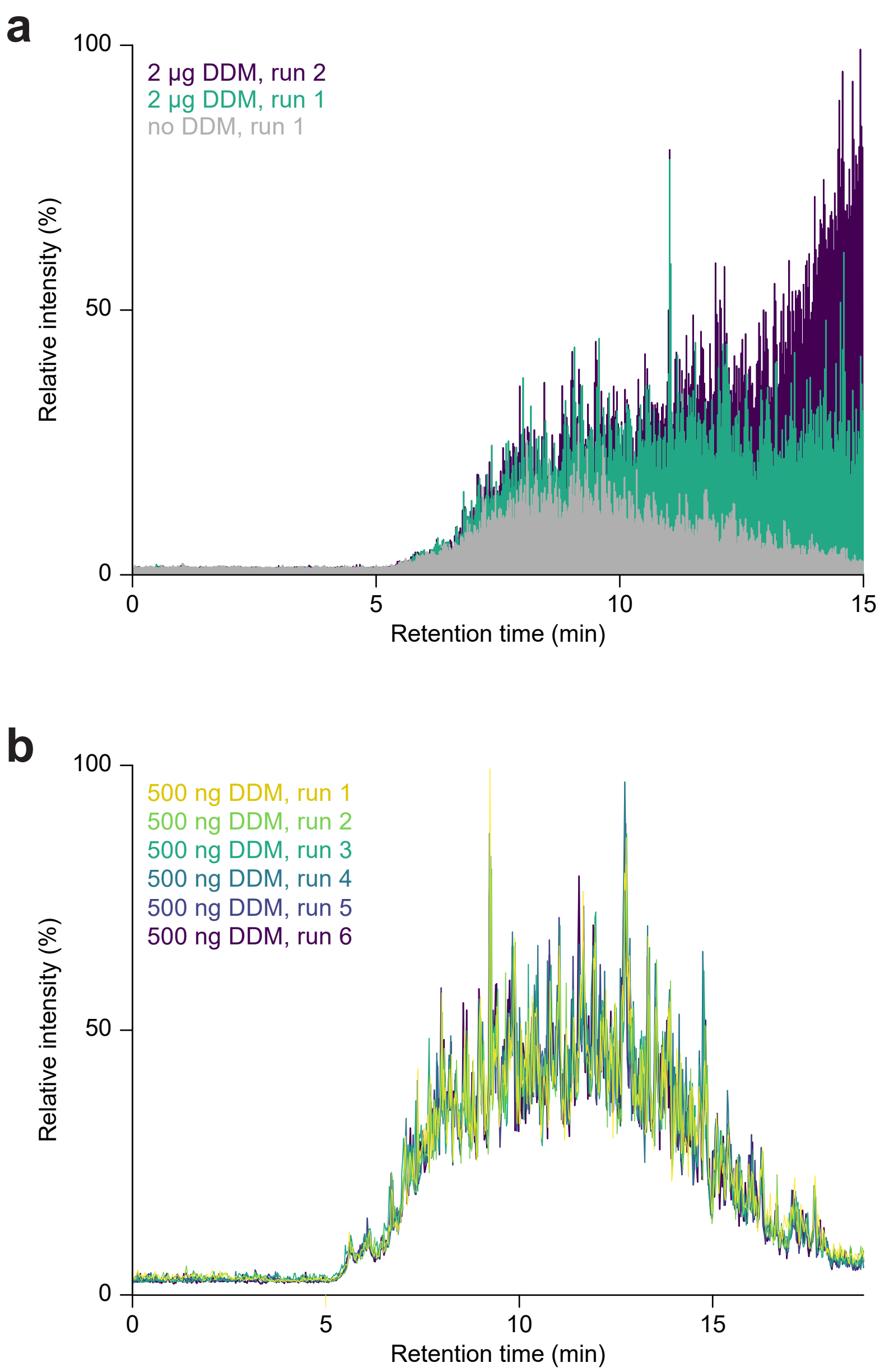

SUPPLEMENTARY FIGURE 14

Supplementary Figure 14. Risk of DDM accumulation when using excessive amounts. (A) Representative total ion current (TIC) chromatograms (data from figure 1B) illustrating accumulation of hydrophobic signal following repeated injections of high amounts of DDM along with 250 pg of HeLa lysate. Shown are consecutive injections containing 2 µg DDM ("DDM, run 1" and "DDM, run 2") compared to a control injection without DDM. (B) Representative TIC chromatograms (data from figure 1C) demonstrating no build-up of hydrophobic signal when injecting 500 ng DDM along with 250 pg of HeLa lysate, indicating that DDM is sufficiently cleared from the analytical column between runs.

SUPPLEMENTARY NOTE 3

To investigate whether the higher-flow methodology employing rapid pressure reduction from ~1000 to ~250 bar impacted chromatographic peak quality, we performed a comparative analysis of peak symmetry and Full Width Half Maximum (FWHM) profiles between constant-flow and higher-flow methods (Supplementary Figure 15). Peak symmetry distributions approximated a normal distribution centered near zero (Supplementary Figure 15A), and we did not observe notable increases in either peak fronting or peak tailing for the higher-flow methods compared to standard constant-flow chromatography. Likewise, precursor FWHM distributions were highly similar between methods (Supplementary Figure 15B), with median FWHM values of ~2.4 sec observed across all conditions.

When visualizing FWHM as a function of retention time (Supplementary Figure 15C), the higher-flow methods displayed a reproducible oscillatory fluctuation in FWHM throughout the active peptide elution window, with median FWHM periodically varying between approximately ~2.2 and ~2.6 sec. We speculate that this behavior reflects gradual depressurization of the pre-compressed gradient prior to establishment of lower-flow analytical conditions. Importantly, however, these oscillations did not result in substantial peak broadening overall, nor did they negatively impact identification confidence, as protein q-values remained stable throughout the gradient (Supplementary Figure 15C).

Finally, the oscillatory FWHM pattern observed for the higher-flow 72 SPD method was highly reproducible across technical replicates (Supplementary Figure 15D), likely facilitating robust chromatographic alignment and quantitative analysis despite the dynamic pressure transition. Collectively, these analyses demonstrate that the higher-flow methodology does not substantially compromise chromatographic peak shape or width relative to standard constant-flow operation, while enabling reduced column delay and increased effective peptide elution time.

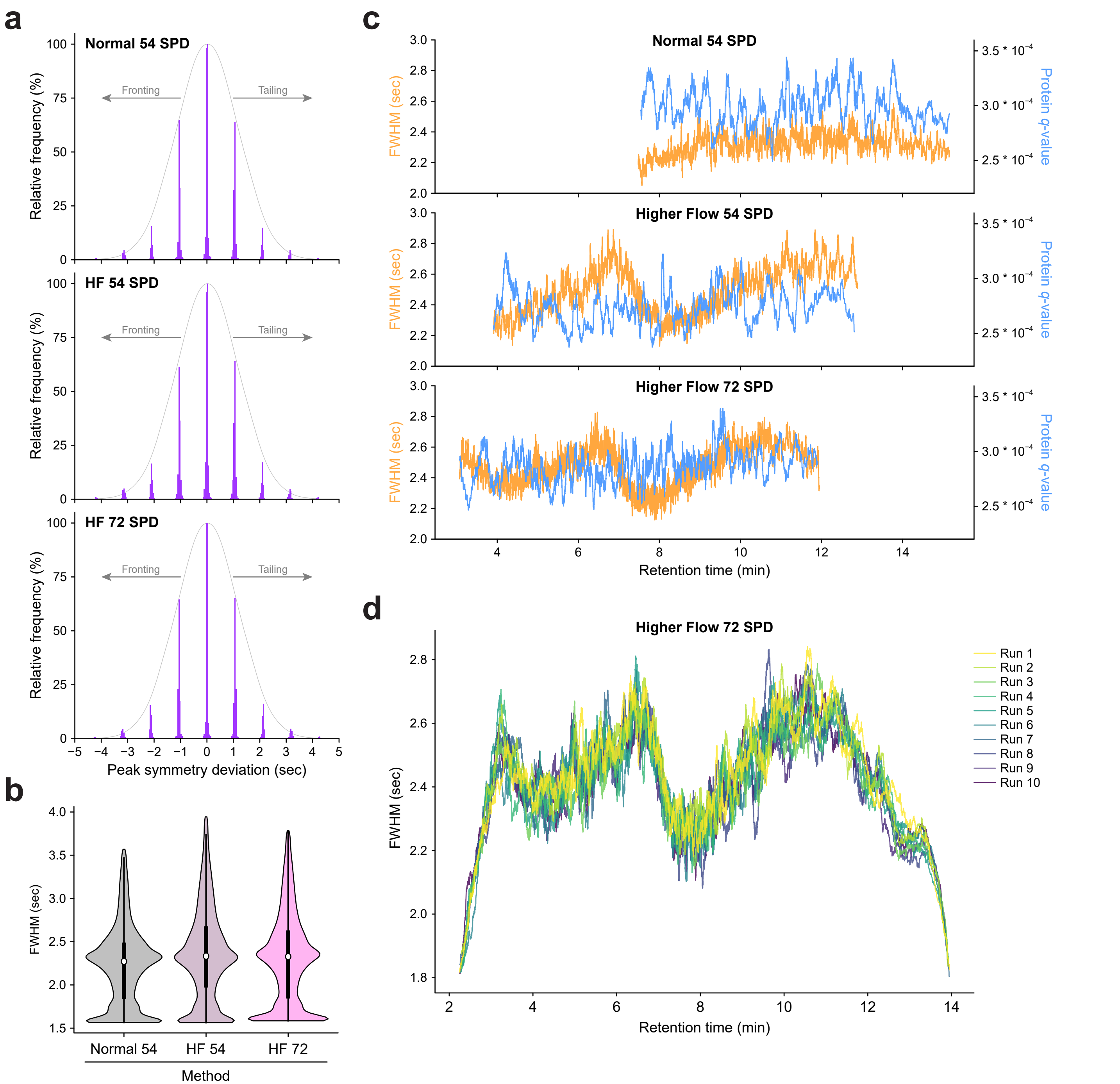

SUPPLEMENTARY FIGURE 15

Supplementary Figure 15. Analysis of peak shape and width in the context of higher-flow methods with variable pressure. (A) Representation of peak symmetry deviation, based on peak initiation, termination, and determined apex retention time. A normal distribution (in grey) was superimposed on the data. Underlying LC-MS data are detailed in **Figure 1G** and **Supplementary Figure 1J-L**. Peak analysis was performed on first pass DIA-NN output. *n* = 6-10 technical replicates. "Normal"; standard steady-flow chromatography, "HF"; method employing Higher Flow (i.e. 500 nL/min) at the start of the gradient, "SPD"; samples per day. (B) As **A**, but visualization of the distribution of Full Width Half Maximum (FWHM) retention time for all precursors identified within the 5th and 95th percentile of identifications across the gradient. Circle; median, thick line limits; 1st and 3rd quartile, thin line limits; 5th and 95th percentile. (C) As **B**, but displaying a 300-precursor running average FWHM (in orange) and corresponding 2000-point running average of protein *q*-values (in blue) as a function of retention time, limited within the 5th and 95th percentile of identifications across the gradient. (D) As **C**, but visualizing FWHM for all precursors across the entire gradient on a per-run basis, *n* = 10 technical replicates.

SUPPLEMENTARY NOTE 4

We evaluated several additional MS-technical parameters to optimize LC-MS settings for single-cell proteomics (Supplementary Figure 16). Firstly, we performed a brief investigation of different compensation voltages when utilizing the FAIMS interface, ranging from −55 V to −35 V in steps of 5 V. From this, we observed the best performance at a CV of −45 V at both protein (Supplementary Figure 16A) and precursor (Supplementary Figure 16B) levels. A CV of −50 V was very close in performance, suggesting the optimum CV may be somewhere between −50 V and −45 V. We performed a more granular investigation of optimum CV further on in the study (Fig. 2D-E).

Next, we compared the “All” loop control setting, wherein all DIA scans in the experiment are performed prior to the MS1 full scan, to “Time” control at 0.6 seconds, wherein an MS1 full scan is inserted every 0.6 seconds independent of the DIA scans. In our case, “All” loop control resulted in an MS1 scan every ~1 second, thus resulting in fewer full scans and more injection time allocated to sampling ions for DIA scans. Overall, we observed higher numbers of proteins (Supplementary Figure 16C) and precursors (Supplementary Figure 16D) identified using “All” control, as well as a slightly improved MS2-based quantitative precision, for both the standard and ultra-sensitive nDIA methods. Notably, this comes at the cost of fewer MS1 scans and thus reduced ability to trace and quantify precursors at the MS1 level.

Finally, we investigated the effect of different fragment ion scan ranges, from 100-2,000 m/z to 150-1,500 m/z. While wider ranges in theory capture more diverse fragments and would thus contain more precursor information, very wide ranges (over ~15-fold between min. and max.) can have reduced transmission. We found that the narrowest range tested, 150-1,500 m/z, resulted in the highest number of identified proteins (Supplementary Figure 16E) and precursors (Supplementary Figure 16F).

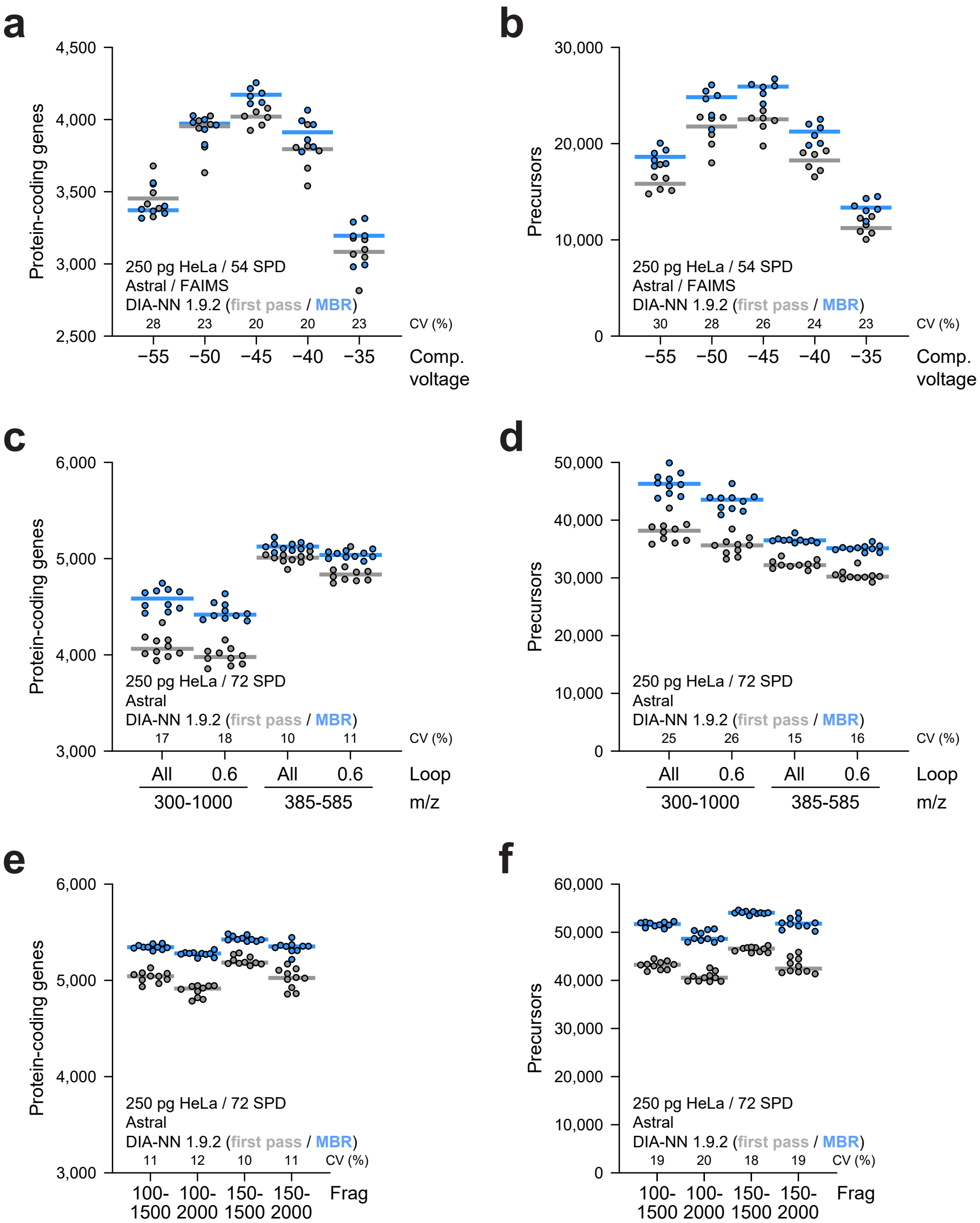

SUPPLEMENTARY FIGURE 16

Supplementary Figure 16. Further optimization of MS-technical method parameters. (A) Number of protein-coding genes identified when analyzing samples with a high-field asymmetric-waveform ion-mobility spectrometry (FAIMS) device, at the indicated compensation voltages (CV; on x-axis), for analysis of 250 pg HeLa. All instrument settings are detailed in Supplementary Data 1. Bars represent median, with gray and blue corresponding to data analysis using DIA-NN in first pass mode and MBR mode, respectively. The numbers above the x-axis represent median coefficient of variation percentage, *n* = 6 technical replicates. (B) As **A**, but showing number of precursors. (C) As **A**, but comparing the “All” and 0.6 seconds loop control settings, for both the 300-1,000 *m*/*z* and 385-585 *m*/*z* scanning methods, for analysis of 250 pg HeLa without FAIMS installed. *n* = 10 technical replicates. (D) As **C**, but showing number of precursors. (E) As **A**, but comparing different fragment recording ranges (“Frag”), for analysis of 250 pg HeLa without FAIMS installed. *n* = 10 technical replicates. (F) As **E**, but showing number of precursors.

SUPPLEMENTARY NOTE 5

The Orbitrap Astral Zoom MS features a low input mode (LIM), which increases sensitivity for low input samples. For this study, we had access to a prototype Orbitrap Astral Zoom MS that allowed us to manually configure the LIM by adjusting the photomultiplier tube (PMT) detector voltage, enabling us to perform a more in-depth evaluation (Supplementary Figure 17). To this end, we increased the PMT voltage from its automatically calibrated base value of 563 V in steps of +25 V (+4.4%), up to +125 V (+22.2%). At both the precursor (Supplementary Figure 17A-C) and protein (Supplementary Figure 17B-D) levels, we observed an optimum around +50 V (+8.9% from base PMT) and +75 V (+13.3% from base PMT), with the LIM overall yielding 11.2% more precursors and 6.5% more proteins from 250 pg of HeLa lysate. When quantifying at the MS2 level, coefficient of variation was also reduced (Supplementary Figure 17E-F), demonstrating that this enhanced sensitivity does not come at a loss of quantitative accuracy. Notably, while we manually configured the PMT voltage on our prototype mass spectrometer, the commercially released Orbitrap Astral Zoom MS automatically calibrates the LIM to achieve optimal performance and robustness.

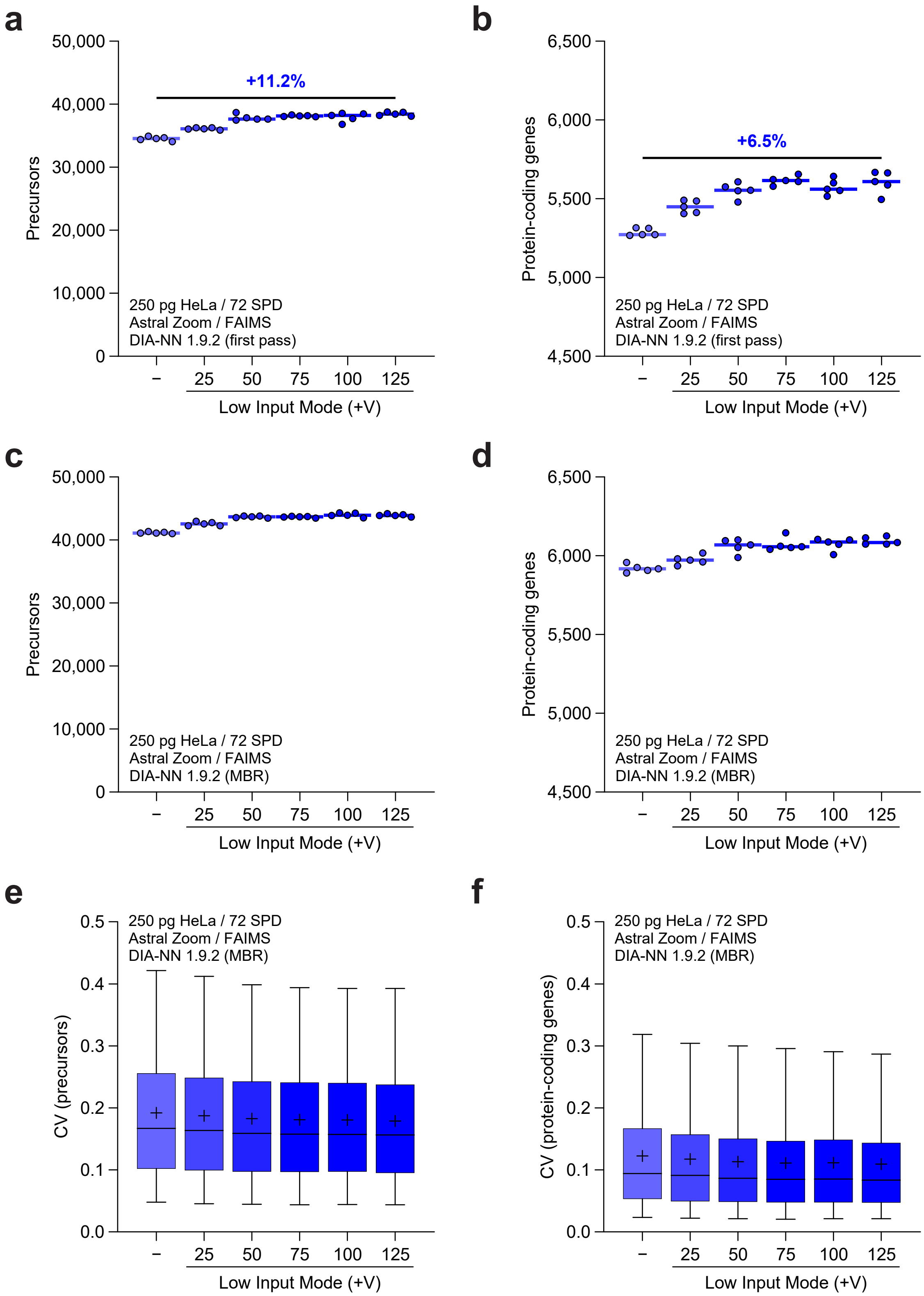

SUPPLEMENTARY FIGURE 17

Supplementary Figure 17. Evaluation of Low Input mode on the Orbitrap Astral Zoom MS. (A) Number of precursors identified from 250 pg HeLa lysate when utilizing standard analysis mode or a manually adjusted PMT voltage to emulate Low Input mode. Bars represent median, percentage increase of median value is indicated. Data analysis using DIA-NN v1.9.2 in first pass mode, *n* = 5 technical replicates. (B) As **A**, but for protein-coding genes. (C) As **A**, but using matching-between-runs (MBR) with DIA-NN. (D) As **B**, but using MBR. (E) Coefficient of variation for all identified precursors as a box plot. Central line; median, plus symbol; average, box limits; 1st and 3rd quantile, whisker limits; 5th and 95th percentile. Data analysis using DIA-NN v1.9.2 with matching-between-runs, with quantification at the MS/MS level. (F) As **E**, but for protein-coding genes.

SUPPLEMENTARY NOTE 6

The initial narrow scan range of 385–585 m/z was selected based on the *in silico* precursor distribution analysis presented in Figure 1D-F. This analysis indicated that a 200 Th window centered around the precursor region contributing the greatest protein coverage would maximize ion accumulation time while maintaining broad proteome coverage. Accordingly, this acquisition strategy was used throughout the initial stages of method development and evaluation.

Subsequently, experimental gas-phase fractionation of the 300–1000 Th precursor range (Figure 2C) enabled direct assessment of protein identification rates across consecutive 50 Th mass intervals. These experiments indicated that the optimal precursor distribution was shifted modestly toward higher m/z values compared to the original *in silico* prediction. Based on these observations, we refined the scan range to 425–625 Th for subsequent method development.

To directly compare both acquisition strategies, we analyzed 250 pg HeLa lysate using otherwise identical DIA acquisition parameters (Supplementary Figure 18). The refined 425–625 Th method consistently outperformed the original 385–585 Th window, increasing precursor identifications by approximately 7% and protein-coding gene identifications by approximately 4%, while maintaining essentially identical quantitative precision (Supplementary Figure 18A-B). These results demonstrate that the experimentally refined scan range provides a modest but reproducible improvement over the original *in silico*-derived scan range. Importantly, however, both scan ranges produced highly comparable results and support the principal conclusions of this study, indicating that the refinement represents optimization of the same underlying acquisition concept rather than a fundamentally different analytical strategy.

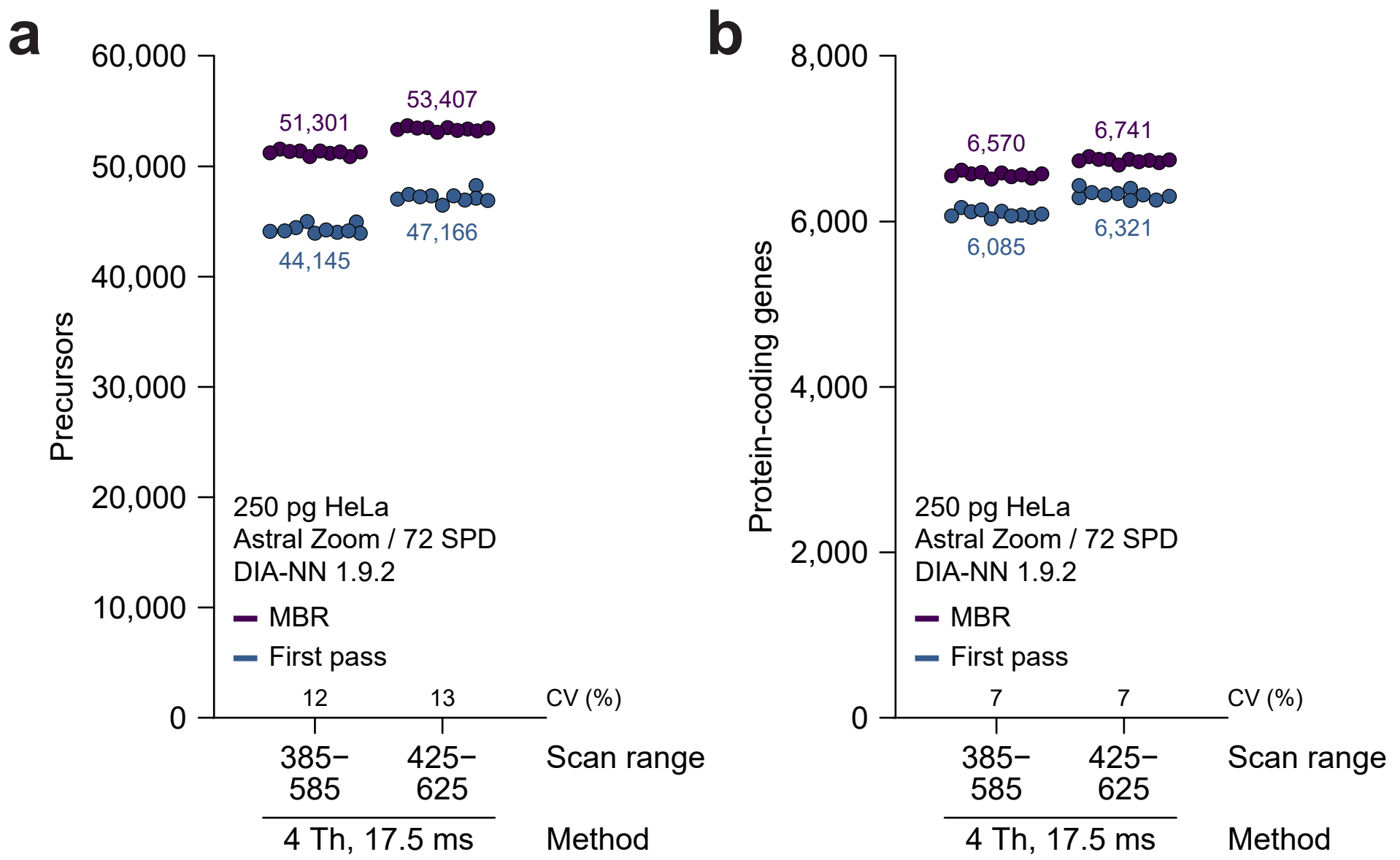

SUPPLEMENTARY FIGURE 18

Supplementary Figure 18. Comparison of 385-585 and 425-625 methods. (A) Number of precursors identified from 250 pg HeLa lysate. All analyses performed using the Orbitrap Astral Zoom MS, and data analyzed using DIA-NN in either first pass or matching-between-runs (MBR) mode. Exact median values are displayed above each group of data for MBR, and below each group of data for first pass. Numbers above the x-axis represent median coefficient of variation percentage, *n* = 10 technical replicates. (B) As **A**, but for protein-coding genes.

SUPPLEMENTARY NOTE 7

To investigate how using a narrow scan range of 425−625 *m/z* would impact quantitative accuracy, we performed a dilution series experiment wherein we evaluated both the narrow-range method and a wide-range 300−1000 *m/z* method, with 5 pg of HeLa lysate as the lowest load and 2500 pg as the highest load (Supplementary Figure 19A-B). We observed that at loads of 50 pg and above, the 300−1000 method identified more precursors, whereas at 2500 pg loads the 300−1000 also identified more protein-coding genes. Notably, when factoring in MBR across all loads, the break-even points were at 25 pg and 500 pg, for precursors and protein-coding genes, respectively.

When evaluating agreement in protein abundance measurements between the narrow- and wide-range methods, we found very strong agreement between both methods at loads ranging from 100 to 1000 pg (Supplementary Figure 19C). At 2500 pg, where the broader scan-range method identifies substantially more peptides, we observed a very modest ratio compression, and similarly at low loads of 5-25 pg we observed a modest reduction in linearity. Interestingly, in both low- and high-load scenarios, the narrow-range method underestimated protein abundance relative to the wide-range method.

Reassuringly, when looking at median absolute deviation on a replicate-to-replicate basis, we found that the narrow-range 425−625 method was overall more robust compared to the wide-range method, with median deviation remaining below ~15% even at 5 pg loads (Supplementary Figure 19D). Notably, the deviation observed at 5 pg was largely consistent across replicate-to-replicate, cross-load, and cross-method comparisons, indicating that the increased variance primarily reflects the stochastic nature of ultra-low-input measurements rather than additional distortion introduced by the narrow scan-range method. Importantly, the intended operating range of 25-250 pg showed the strongest proportionality between methods, with slopes approaching unity and low inter-replicate deviation.

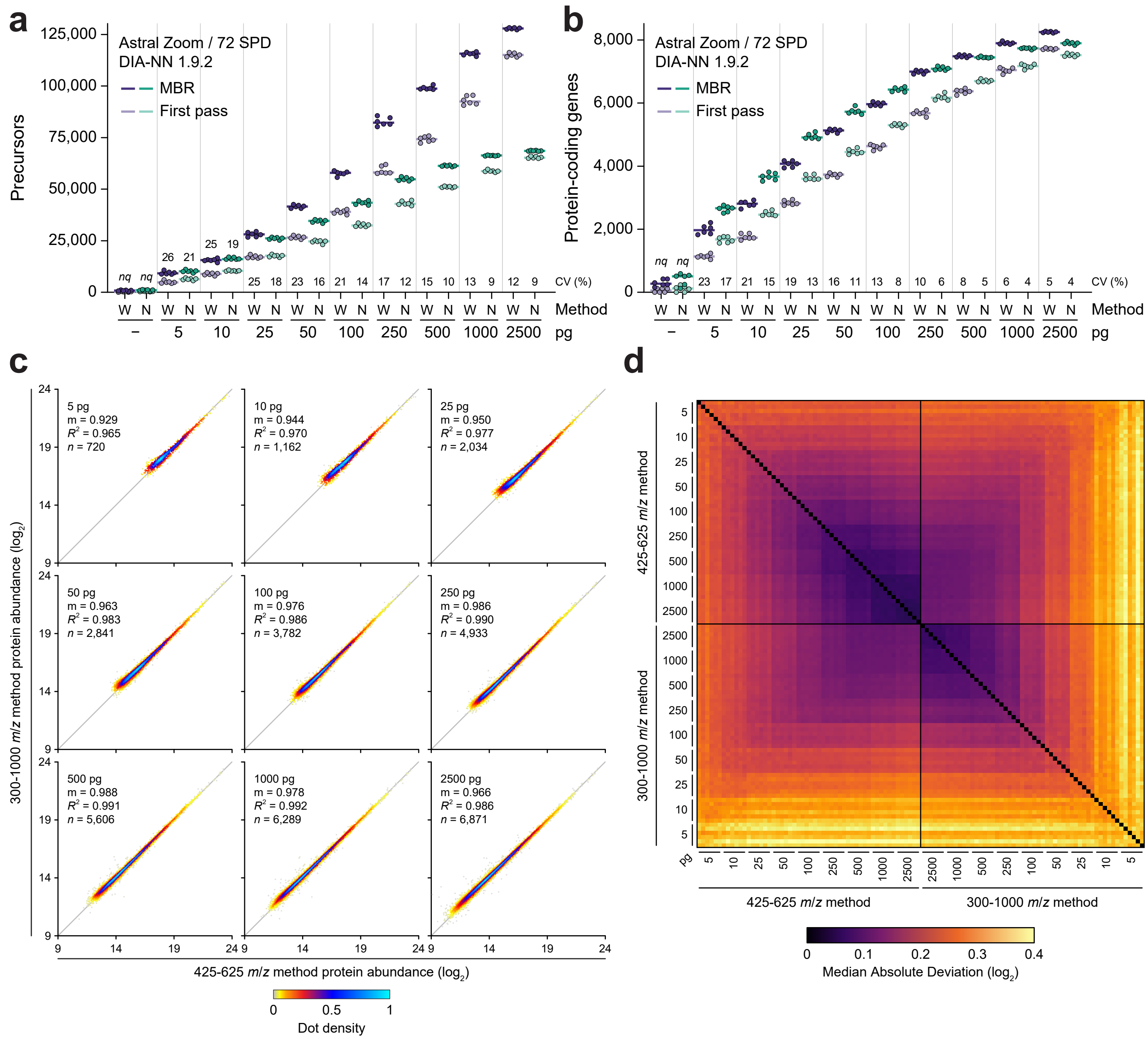

SUPPLEMENTARY FIGURE 19

Supplementary Figure 19. Assessment of quantitative accuracy between 300−1000 and 425−625 m/z scan range methods. (A) Dilution series experiment, ranging from 5 pg to 2500 pg of injected HeLa lysate, comparing a wide scan range of 300−1000 (“W”) scan range to a narrow scan range of 425−625 (“N”). All injections were performed in ascending amounts, starting with a blank to ensure negligible sample carry-over. Number of identified precursors are indicated, bars represent median, with data analysis using DIA-NN v1.9.2 in first pass mode and MBR mode, as indicated. Numbers above the x-axis represent median coefficient of variation percentage, $n = 6$ technical replicates. (B) As **A**, but showing protein-coding genes. (C) Scatter plot analysis of median $\log_2$ abundance values, based on first pass (no MBR) data, comparing the wide and narrow methods at each different load. Each dot represents one protein-coding gene quantifiable in both methodologies (with n indicated). The grey line is drawn at x=y (i.e. perfect accuracy). The m value indicates slope of linear regression, R^2 indicates the squared Pearson correlation. (D) Heatmap analysis, visualizing the median absolute deviation (MAD) between $\log_2$ abundance values on a per-replicate basis, and calculated using first pass data.

SUPPLEMENTARY NOTE 8

The majority of comparisons presented throughout this study evaluate optimized narrow-range acquisition strategies (e.g. 425–625 m/z) against a broad 300–1000 m/z scan range. Although this comparison illustrates the benefit of restricting the precursor space for ultra-low-input proteomics, intermediate scan ranges around 400–800 m/z represent another established acquisition strategy for low-input proteomics, particularly in combination with FAIMS. To provide a more comprehensive comparison, we therefore directly evaluated three acquisition strategies using 25 pg HeLa lysate: a broad 300–1000 m/z method, an intermediate 400–800 m/z method, and our optimized 425–625 m/z ultra-sensitive nDIA method (Supplementary Figure 20). In all cases, analysis was performed without FAIMS, and isolation window width was fixed at 4 Th with maximum injection times adjusted to maintain identical cycle times.

The intermediate 400–800 m/z method identified the greatest number of precursors, exceeding both the broad 300–1000 m/z and narrow 425–625 m/z acquisition strategies (Supplementary Figure 20A). However, despite this increase in precursor identifications, the 400–800 m/z method identified fewer protein-coding genes than the 425–625 m/z method (Supplementary Figure 20B), and exhibited lower quantitative precision, reflected by higher median coefficients of variation. In contrast, the 425–625 m/z acquisition strategy produced the highest number of protein-coding genes together with the lowest quantitative variability across replicate analyses.

These observations are consistent with the central principle underlying the ultra-sensitive nDIA workflow presented in this study. Progressively restricting the scan range reduces precursor complexity and increases ion accumulation time for each isolation window. Although wider scan ranges sample a larger peptide population, the more focused 425–625 m/z range preferentially targets the precursor region yielding the highest number of protein identifications per acquired precursor, thereby maximizing proteome depth while maintaining excellent quantitative reproducibility. Collectively, these data further support the selection of the 425–625 m/z range as the preferred acquisition strategy for label-free low-input proteomics in the absence of FAIMS.

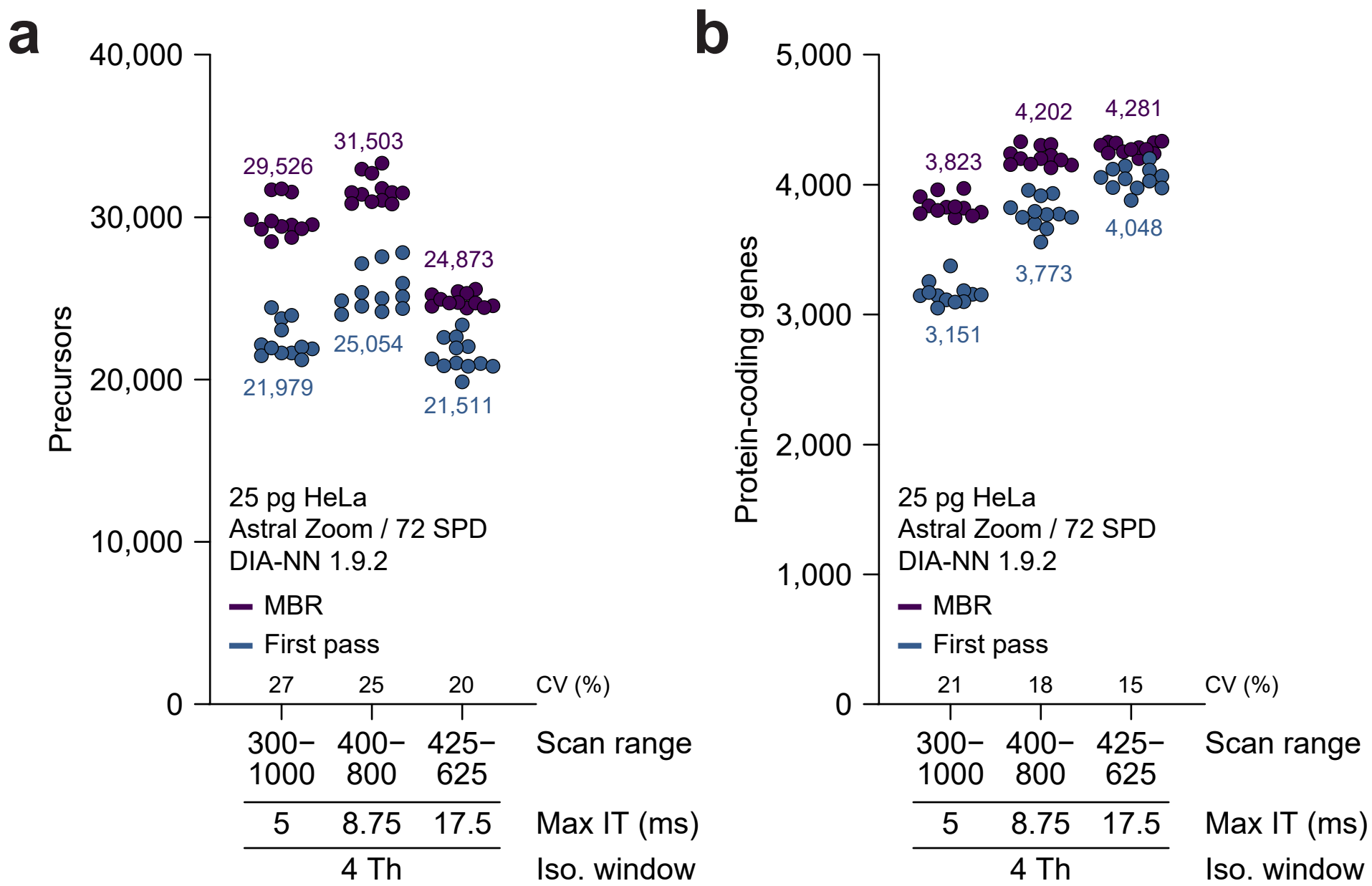

SUPPLEMENTARY FIGURE 20

Supplementary Figure 20. Comparison of 300-1000, 400-800, and 425-625 methods. (A) Number of precursors identified from 25 pg HeLa lysate. All analyses performed using the Orbitrap Astral Zoom MS, and data analyzed using DIA-NN in either first pass or matching-between-runs (MBR) mode. Exact median values are displayed above each group of data for MBR, and below each group of data for first pass. Numbers above the x-axis represent median coefficient of variation percentage, *n* = 12 technical replicates. "Max IT"; maximum injection time, "Iso. window"; isolation window. (B) As **A**, but for protein-coding genes.

SUPPLEMENTARY NOTE 9

To investigate the computational proteome carrier effect, we used the CellenOne to sort either one, two, five, or twenty HEK293T cells, into single wells of a 96-well plate, prior to protein extraction and proteolytic digestion (Supplementary Figure 21). Following MS data acquisition, we either searched the cells in computational isolation (no MBR), or performed match-between runs (MBR) together with other raw LC-MS files containing either the same or larger numbers of cells.

When considering just single HEK cells (median diameter 24.3 μm) without MBR, we could identify a median of ~3,700 proteins per cell (Supplementary Figure 21A). When matching all single cells to each other, this number increased to ~4,600 proteins per cell, and further increased to ~5,100 proteins per cell when also including 2-, 5-, and 20-cell runs in the MBR. Interestingly, this depth of sequencing was similar to what we identified from 2-cell runs without MBR (Supplementary Figure 21A), with application of MBR with 2-cell runs further increasing numbers from ~5,800 to ~6,100 depending on cell multiplicity in the MBR. The computational carrier effect appeared more potent for 2-cell runs, with their MBR runs exceeding the number of identifications from 5-cell runs without MBR, and 5-cell runs with MBR matching 20-cell runs without MBR. Similar trends were observed at the precursor level (Supplementary Figure 21B).

Finally, we observed that co-digestion of multiple cells resulted in a reduction of median CV, whereas MBR to increasing cell multiplicity did not favorably affect CV. The reduction of median CV with increasing cell count could have various technical reasons, such as a higher input amount of protein, but at the same time biological cell-to-cell variance would also be ameliorated by co-digestion of multiple cells.

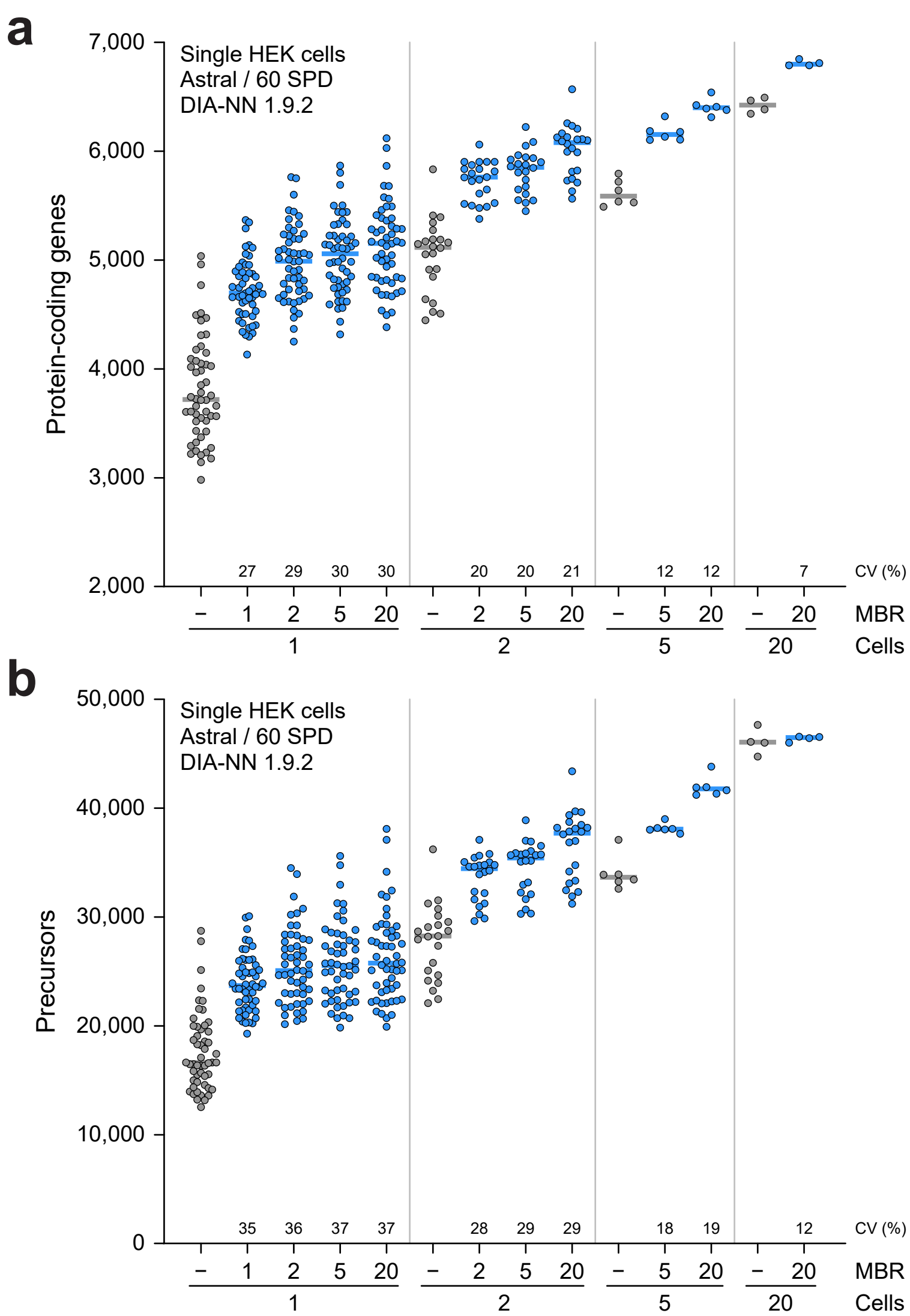

SUPPLEMENTARY FIGURE 21

Supplementary Figure 21. Evaluation of the carrier effect when analyzing single or multiple HEK cells. (A) Overview of the number of protein-coding genes identified from single or multiple HEK cells, following fully automated handling and digestion using the CellenOne (see also **Figure 4A**). Cells were sorted as 1 cell, 2 cells, 5 cells, or 20 cells, and analyzed using an Orbitrap Astral MS. Data analysis was performed using DIA-NN, either in first pass mode (in gray) or using matching-between runs (in blue) to all other 1 cell, 2 cell, 5 cell, or 20 cell runs, as indicated. Bars represent median. Numbers above the x-axis are median coefficient of variation. *n* = 52 (×1 cell), 22 (×2 cells), 6 (×5 cells), 4 (×20 cells). (B) As **A**, but for precursors.

SUPPLEMENTARY REFERENCES

1. Ye Z, *et al.* Enhanced sensitivity and scalability with a Chip-Tip workflow enables deep single-cell proteomics. *Nat Methods* **22**, 499-509 (2025).

2. Bubis JA, *et al.* Challenging the Astral mass analyzer to quantify up to 5,300 proteins per single cell at unseen accuracy to uncover cellular heterogeneity. *Nat Methods* **22**, 510-519 (2025).

3. Szklarczyk D, *et al.* The STRING database in 2023: protein-protein association networks and functional enrichment analyses for any sequenced genome of interest. *Nucleic Acids Res* **51**, D638-D646 (2023).

4. Koncarevic S, Lossner C, Kuhn K, Prinz T, Pike I, Zucht HD. In-depth profiling of the peripheral blood mononuclear cells proteome for clinical blood proteomics. *Int J Proteomics* **2014**, 129259 (2014).

5. Rieckmann JC, *et al.* Social network architecture of human immune cells unveiled by quantitative proteomics. *Nat Immunol* **18**, 583-593 (2017).

6. Shen L, *et al.* Proteomics Study of Peripheral Blood Mononuclear Cells (PBMCs) in Autistic Children. *Front Cell Neurosci* **13**, 105 (2019).

7. Li Y, *et al.* Combined proteomics and single cell RNA-sequencing analysis to identify biomarkers of disease diagnosis and disease exacerbation for systemic lupus erythematosus. *Front Immunol* **13**, 969509 (2022).

8. Santa C, *et al.* Proteomic analysis of peripheral blood mononuclear cells in first episode psychosis - Protein and peptide-centered approaches to elucidate potential diagnostic biomarkers. *J Proteomics* **309**, 105296 (2024).

9. Giammaria S, *et al.* Profiling of the Peripheral Blood Mononuclear Cells Proteome by Shotgun Proteomics Identifies Alterations of Immune System Components, Proteolytic Balance, Autophagy, and Mitochondrial Metabolism in Glaucoma Subjects. *ACS Omega* **10**, 14866-14883 (2025).

10. Milacic M, *et al.* The Reactome Pathway Knowledgebase 2024. *Nucleic Acids Res* **52**, D672-D678 (2024).
