# Supplementary material for: Extending single-cell proteomics from HeLa to small primary human immune cells
Source: Nat Commun. 2026 Sep 19;17:10985. doi: 10.1038/s41467-026-77675-x (PMC13683416; doi:10.1038/s41467-026-77675-x)
Supplement: Supplementary file 2 [file 41467_2026_77675_MOESM2_ESM.pdf]

Description of Additional Supplementary Files

File Name: Supplementary Data 1

Description: A comprehensive overview of all LC-MS acquisition parameters for all experiments in this work, as well as which datasets correspond to which figures.

File Name: Supplementary Data 2

Description: Overview of proteins identified from single HeLa cells in this study compared to two recent studies using similar single-cell approaches (Ye et al., 2025 & Bubis et al., 2025). See also Figure 7 and Supplementary Figure 10.

File Name: Supplementary Data 3

Description: Overview of proteins identified from single PBMCs in this study compared to six other studies using bulk PBMC proteomics approaches (Končarević et al., 2014, Rieckmann et al., 2017, Shen et al., 2019, Li et al., 2022, Santa et al., 2024 & Giammaria et al., 2025). See also Figure 8 and Supplementary Figure 11.

File Name: Supplementary Data 4

Description: Uniprot keywords, term enrichment analysis via the STRING database. Related to Figure 7C.
