# Supplementary material for: Extending single-cell proteomics from HeLa to small primary human immune cells
Source: Nat Commun. 2026 Sep 19;17:10985. doi: 10.1038/s41467-026-77675-x (PMC13683416; doi:10.1038/s41467-026-77675-x)
Supplement: Supplementary file 3 [file 41467_2026_77675_MOESM7_ESM.pdf]

nature portfolio

Corresponding author(s): Jesper V. Olsen

Last updated by author(s): Aug 14, 2026

Reporting Summary

Nature Portfolio wishes to improve the reproducibility of the work that we publish. This form provides structure for consistency and transparency in reporting. For further information on Nature Portfolio policies, see our Editorial Policies and the Editorial Policy Checklist.

Statistics

For all statistical analyses, confirm that the following items are present in the figure legend, table legend, main text, or Methods section.

n/a	Confirmed	
☐	☒	The exact sample size (*n*) for each experimental group/condition, given as a discrete number and unit of measurement
☐	☒	A statement on whether measurements were taken from distinct samples or whether the same sample was measured repeatedly
☐	☒	The statistical test(s) used AND whether they are one- or two-sided<br>*Only common tests should be described solely by name; describe more complex techniques in the Methods section.*
☒	☐	A description of all covariates tested
☐	☒	A description of any assumptions or corrections, such as tests of normality and adjustment for multiple comparisons
☐	☒	A full description of the statistical parameters including central tendency (e.g. means) or other basic estimates (e.g. regression coefficient) AND variation (e.g. standard deviation) or associated estimates of uncertainty (e.g. confidence intervals)
☐	☒	For null hypothesis testing, the test statistic (e.g. *F*, *t*, *r*) with confidence intervals, effect sizes, degrees of freedom and *P* value noted<br>*Give P values as exact values whenever suitable.*
☒	☐	For Bayesian analysis, information on the choice of priors and Markov chain Monte Carlo settings
☒	☐	For hierarchical and complex designs, identification of the appropriate level for tests and full reporting of outcomes
☐	☒	Estimates of effect sizes (e.g. Cohen's *d*, Pearson's *r*), indicating how they were calculated

Our web collection on statistics for biologists contains articles on many of the points above.

Software and code

Policy information about availability of computer code

Data collection: All MS data files were analyzed with DIA-NN v1.9.2 or Spectronaut 19 (Biognosys). Statistical handling of the data was primarily performed using the freely available Perseus v1.5.5.3 software, and includes calculation of Pearson correlation and linear regression, hierarchical clustering, and dimensionality reduction (UMAP) analysis. Venn diagram overlaps were performed using Venny v2.1 (https://bioinfogp.cnb.csic.es/tools/venny/), and scaled Venn diagrams were rendered using eulerAPE v2 (https://www.eulerdiagrams.com/eulerAPE/v2/). UpSet plots were generated using ChiPlot (https://www.chiplot.online/upset_plot.html). Boxplots were drawn using BoxPlotR (http://shiny.chemgrid.org/boxplotr/). Violin plots were drawn using GraphPad Prism v11.0.1. Term enrichment analyses (Uniprot keywords, Reactome, TISSUES) were performed using the STRING database, v12.0. The "T Cell Receptor Signaling Pathway" was adapted from Kyoto Encyclopedia of Genes and Genomes (KEGG), v116.0. Reactome Pathway enrichment analysis was performed using and curated by Reactome, v94. Overall, plotting of graphs was done using either Perseus, GraphPad Prism, or Microsoft Excel (Office Professional Plus 2016).

Data analysis: All MS data files were analyzed with DIA-NN v1.9.2 or Spectronaut 19 (Biognosys). Statistical handling of the data was primarily performed using the freely available Perseus v1.5.5.3 software, and includes calculation of Pearson correlation and linear regression, hierarchical clustering, and dimensionality reduction (UMAP) analysis. Venn diagram overlaps were performed using Venny v2.1 (https://bioinfogp.cnb.csic.es/tools/venny/), and scaled Venn diagrams were rendered using eulerAPE v2 (https://www.eulerdiagrams.com/eulerAPE/v2/). UpSet plots were generated using ChiPlot (https://www.chiplot.online/upset_plot.html). Boxplots were drawn using BoxPlotR (http://shiny.chemgrid.org/boxplotr/). Violin plots were drawn using GraphPad Prism v11.0.1. Term enrichment analyses (Uniprot keywords, Reactome, TISSUES) were performed using the STRING database, v12.0. The "T Cell Receptor Signaling Pathway" was adapted from Kyoto Encyclopedia of Genes and Genomes (KEGG), v116.0. Reactome Pathway enrichment analysis was performed using and curated by Reactome, v94. Overall, plotting of graphs was done using either Perseus, GraphPad Prism, or Microsoft Excel (Office Professional Plus 2016).

For manuscripts utilizing custom algorithms or software that are central to the research but not yet described in published literature, software must be made available to editors and reviewers. We strongly encourage code deposition in a community repository (e.g. GitHub). See the Nature Portfolio guidelines for submitting code & software for further information.

Data

Policy information about availability of data

All manuscripts must include a data availability statement. This statement should provide the following information, where applicable:

- Accession codes, unique identifiers, or web links for publicly available datasets
- A description of any restrictions on data availability
- For clinical datasets or third party data, please ensure that the statement adheres to our policy

The mass spectrometry proteomics data generated in this study have been deposited in the ProteomeXchange Consortium via the PRIDE partner repository, with the dataset identifiers PXD070599 (primary data), PXD078497 (revision 1 data), and PXD080728 (revision 2 data).

PXD070599 [http://proteomecentral.proteomexchange.org/cgi/GetDataset?ID=PXD070599] (primary data)
PXD078497 [http://proteomecentral.proteomexchange.org/cgi/GetDataset?ID=PXD078497] (revision 1 data)
PXD080728 [http://proteomecentral.proteomexchange.org/cgi/GetDataset?ID=PXD080728] (revision 2 data)

All other data generated in this study are provided in the Supplementary Information and Source Data files.

A comprehensive overview of all LC-MS acquisition parameters for all experiments in this work, as well as which datasets correspond to which figures, is provided in Supplementary Data 1.

Research involving human participants, their data, or biological material

Policy information about studies with human participants or human data. See also policy information about sex, gender (identity/presentation), and sexual orientation and race, ethnicity and racism.

Reporting on sex and gender	N/A; donor-level sex/gender information was not available to the study team.
Reporting on race, ethnicity, or other socially relevant groupings	N/A; donor-level race, ethnicity, or other socially relevant grouping information was not available to the study team.
Population characteristics	Anonymous healthy adult blood donors; no individual-level demographic or other covariate information was available to the study team.
Recruitment	No participants were directly recruited for this study. Peripheral blood was obtained as anonymized surplus material from anonymous healthy adult blood donors at the Capital Region Blood Bank, Copenhagen University Hospital–Rigshospitalet.
Ethics oversight	The anonymized sample collection was approved by the local ethics committee (Reference: F-24061507).

Note that full information on the approval of the study protocol must also be provided in the manuscript.

Field-specific reporting

Please select the one below that is the best fit for your research. If you are not sure, read the appropriate sections before making your selection.

[x] Life sciences [ ] Behavioural & social sciences [ ] Ecological, evolutionary & environmental sciences

For a reference copy of the document with all sections, see nature.com/documents/nr-reporting-summary-flat.pdf

Life sciences study design

All studies must disclose on these points even when the disclosure is negative.

Sample size	Sample sizes were selected according to the purpose of each experiment. Quantitative LC-MS comparisons were generally performed using 4–10 technical replicates per condition to assess analytical reproducibility and quantitative robustness, with larger replicate numbers used for key benchmarking experiments. For single-cell proteomics experiments, we generally aimed to analyze at least 10 individual cells per condition to capture cell-to-cell biological heterogeneity in asynchronous populations, while substantially larger cohorts (>50 cells per condition) were used for key benchmarking and biological analyses. Smaller numbers of replicates were used for exploratory experiments focused on method development or parameter screening, where the objective was to characterize performance rather than draw statistical conclusions. No formal statistical power calculation was performed to predetermine sample sizes; sample sizes were chosen based on the intended analytical purpose, expected technical or biological variability, instrument availability, and established practice in low-input and single-cell proteomics. Exact replicate numbers for each experiment are provided in the Supplementary Data 1.
Data exclusions	No data were excluded based on experimental outcome or analytical results. A small number of LC-MS runs were lost due to sporadic instrument hardware or software failures resulting in corrupted raw data files that could not be processed or interpreted. These technical failures were unrelated to sample identity or experimental condition. For single-cell experiments, sufficient replicate numbers were planned to ensure that occasional technical run loss did not compromise downstream analyses or interpretation. No exclusion criteria based on data quality metrics were pre-established beyond removal of irrecoverably corrupted raw data files.

Replication	All experiments were performed with multiple technical and/or biological replicates, as detailed in the figure legends and Methods. Reproducibility was assessed through repeated LC-MS analyses, evaluation of identification overlap and quantitative variation across replicates, and benchmarking across independent experimental conditions and acquisition strategies. Key findings, including improvements in proteome depth, quantitative robustness, and performance across throughput conditions, were consistently reproduced across replicate analyses and independent experiments.
Randomization	Samples were not randomized into experimental groups in the conventional sense, as the majority of experiments were technical benchmarking comparisons in which the same sample batch was analyzed under each condition. Instrument performance was monitored throughout acquisition, and comparative conditions were analyzed using matched sample material to minimize effects of analytical drift. For single-cell experiments, individual cells were isolated without pre-selection beyond the experimental gating or cell-size criteria described in the Methods. To minimize potential positional and dispensing-order effects, LC-MS acquisition order was arranged orthogonally to the CellenOne sorting direction across 96-well plates. For PBMC experiments, cells from each donor were analyzed as obtained, without assignment of individual cells to experimental groups beyond the predefined cell-type/size selection criteria.
Blinding	Blinding was not relevant to this study. The work primarily involved technical and methodological optimization of LC-MS workflows using predefined acquisition parameters and standardized sample inputs. For single-cell experiments, cells were isolated without selection beyond the experimental criteria described in the Methods. Data acquisition and analysis were performed using automated computational workflows and predefined processing parameters. LC-MS acquisition settings necessarily required direct operator control and therefore could not be implemented in a blinded manner.

Reporting for specific materials, systems and methods

We require information from authors about some types of materials, experimental systems and methods used in many studies. Here, indicate whether each material, system or method listed is relevant to your study. If you are not sure if a list item applies to your research, read the appropriate section before selecting a response.

Materials & experimental systems

n/a	Involved in the study	
☒	☐	Antibodies
☐	☒	Eukaryotic cell lines
☒	☐	Palaeontology and archaeology
☒	☐	Animals and other organisms
☒	☐	Clinical data
☒	☐	Dual use research of concern
☒	☐	Plants

Methods

n/a	Involved in the study	
☒	☐	ChIP-seq
☒	☐	Flow cytometry
☒	☐	MRI-based neuroimaging

Eukaryotic cell lines

Policy information about cell lines and Sex and Gender in Research

Cell line source(s)	HeLa cells (CCL-2, female) and HEK293T cells (CRL-3216, female) were obtained from the American Type Culture Collection (ATCC).
Authentication	Cell lines were not additionally authenticated following acquisition from ATCC.
Mycoplasma contamination	All cell lines were routinely tested for mycoplasma contamination and consistently tested negative.
Commonly misidentified lines (See ICLAC register)	HeLa cells are recognized as a commonly cross-contaminating cell line by ICLAC. Both HeLa and HEK293T cells used in this study were obtained directly from ATCC and handled according to standard cell culture procedures.

Plants

Seed stocks	N/A
Novel plant genotypes	N/A
Authentication	N/A
