# Supplementary material for: Extending single-cell proteomics from HeLa to small primary human immune cells
Source: Nat Commun. 2026 Sep 19;17:10985. doi: 10.1038/s41467-026-77675-x (PMC13683416; doi:10.1038/s41467-026-77675-x)
Supplement: Supplementary file 4 [file 41467_2026_77675_MOESM8_ESM.pdf]

nature portfolio

Peer Review File

Extending single-cell proteomics from HeLa to small primary human immune cells

Corresponding Author: Professor Jesper Olsen

This file contains all reviewer reports in order by version, followed by all author rebuttals in order by version.

Version 0:

Reviewer comments:

Reviewer #1

(Remarks to the Author)
This study is a joint effort of the Olsen team and Thermo Scientific, introducing Thermo's new instrument Orbitrap Astral Zoom, and specifically, application to very low protein loads in small single cells. The study meticulously examines many of the parameters of the MS and some of the sample preparation and LC methods and provides a valuable roadmap to the utilization of the instrument for single cell proteomics, including analysis of very small cells of 6-8 um. Among the MS examinations, they compare the Astral-Zoom to the previous version of the orbitrap Astral, they examine the use of FAIMS and thoroughly calibrate the mass ranges and window sizes. Overall, the research is very nicely presented and explained, and it will be very useful to the single-cell proteomics experts among the readers. For the broader audience, the manuscript shows the cutting-edge capabilities of single cell proteomics to date, however many of the details will not be understood by readers outside the proteomics field. Overall, I support publication of this study after answering the following comments:
1. The entire study focuses on proteomic depth, but it mostly analyzes 60 samples per day. Going forward in the field will necessitate increasing the throughput of the single cell measurements. Otherwise, typical experiments of few thousands of cells will take months of measurement. Having such a thorough examination of all the LC-MS parameters is not complete without showing the impact of increased throughput to more than 100 SPD (and even higher).
2. The use of narrow mass ranges has been previously shown to increase identification and reduce CVs. Nevertheless, this narrowing also has limitations as we are sampling fewer peptides. While the authors claim that they retain proteotypicity, they don't actually show that we don't lose protein level quantitative accuracy, compared to broad MZ ranges. Their analyses examine the CV within a condition, this way showing the precision within each method. However, it is also important to examine the correlation of the protein intensities with the ones obtained in the wider ranges and also higher loads. One has to ensure that the peptides within this range are actually representative of the true protein amounts and that we are not skewing results due to this restriction.
3. Related to the previous comment, despite the increase in the protein IDs, the authors show an expected reduction in the number of precursors. It should be emphasized that this could be a major drawback if people are interested in peptide-level analyses, such as modified peptides, etc. This should be mentioned in the discussion and also referred to in the section of the phosphopeptide analysis.
4. Since the authors include multiple analytical parameters, it could be valuable to add small illustrations to present them. For example, in Figure 1, they can add a small scheme of the MZ window size, mass range, number of windows, MS scans in the different conditions, etc. Similarly, a small cartoon showing the estimated protein amounts from cells of different sizes, instead of showing only diameter and volume. It is not always intuitive to switch between the discussion of protein amounts (in the diluted HeLa) to the single cell and PBMC discussion.
5. What is the difference between Figure 1 plots and Figure S1?
6. Figure 2C and related figures in the supplementary material should be separated into two graphs. The X-axis should not be doubled in a single graph.

Reviewer #2

(Remarks to the Author)
In this manuscript, Hendriks and Buch-Larsen et al. describe a stepwise optimization based on previously published Astral and Astral Zoom methods for single-cell–equivalent inputs and small single-cells. The authors systematically revisit method

parameters and demonstrate sensitive detection of phosphorylated peptides at single-cell inputs. While the manuscript is generally well structured and the experiments appear carefully executed, the level of methodological novelty currently does not, in my view, meet the threshold for publication in Nature Communications. My concerns are detailed below.

1. Novelty and relation to prior work. The manuscript would benefit from a clearer delineation of what methodological advances are truly novel beyond previous studies, some of them are indicated below:
A substantial fraction of the optimization presented in Fig. 2 has already been described in previous publications, including Petrosius, Valdemaras et al., Molecular & Cellular Proteomics (2024), and Hsu et al., Journal of Proteome Research (2025) and their own work Guzman et al., Nature Biotechnology (2024).
While the analysis depth of ~3,000 protein-coding genes from small cells is impressive exceeds previous reports, several prior studies have demonstrated deep coverage of small cells (e.g., THP-1 cells ~10 µm; bone marrow cells in work by Specht et al., Genome Biology (2021), Ctortecka et al., Nature Communications (2024) and Furtwängler et al., Science (2025)). Please adapt the tone regarding the novelty of 'small cell analysis' throughout the manuscript.

2. Sample preparation
In Fig. 1b, the impact of DDM on adsorptive losses is evaluated using two different column IDs. While the conclusion that the 50 µm I.D. column performs better is reasonable, the use of 5 µg DDM appears excessive. Most low-input or single-cell proteomics studies use ~0.1% DDM during lysis at very low volumes, corresponding to ~500 ng DDM. Additionally, the subsequent comparison of DDM concentrations in Fig. 1c does not include the originally used 5 µg condition but lower, more widely used concentrations. It is unclear why this concentration was excluded, or alternatively why the experiments in Fig. 1b were performed with such a high amount of DDM. Moreover, the lack of difference between 200 ng and 1 µg DDM is unsurprising.
It is encouraging that reduction and alkylation can be performed without introducing substantial chemical noise to single cell input samples. Please clarify why this optimized sample preparation protocol was subsequently not applied to the PBMC samples.

3. Peak shapes
For the faster column loading and equilibration where pressure is rapidly reduced from 1000 to 250 bar, does this result in any observable peak broadening in comparison to methods at constant flow?
Even at 'high-flow', the reported 4 µL sample volume for single cell samples requires at least ~2 minutes to load (with 500 µL) onto the column, but the active peptide elution time is <15 minutes (Fig. 3c). This appears relatively long and may compromise peak sharpness, please elaborate on FWHM and peak shape for the different chromatographic setups.

4. Identification variability, and quantitative performance
Was the data shown in Fig. 1b analyzed separately from the other experiments? It is unclear how there is only a marginal decrease in identifications when reducing input from 250 pg to 125 pg.
MS1 vs. MS2 quantification is only shown down to 50 pg input, which is relatively high compared to other experiments detailed in the manuscript. Please justify this choice and elaborate on the high CVs of their 'standard' MS2 level quantification at 50pg input.
Based on the presented data, one could conclude that MS1-based quantification on the Astral may be beneficial for single-cell or low-input analyses. Why was this strategy not evaluated on the true single-cell datasets?

5. Acquisition method design
The authors demonstrate that the new ultra-sensitive nDIA methods yield more identifications than the standard nDIA approach. However, especially in combination with FAIMS smaller isolation windows appear to increase CVs without providing a clear benefit in identifications (Fig. 2e). This seems consistent with prior observations (e.g., Bubis et al., Nature Methods, 2025) suggesting that larger isolation windows combined with longer injection times may be more appropriate for ultra-low input samples. Please elaborate on the benefits of nDIA in comparison to wider isolation window methods especially in the context of single-cell.
In Fig. S8i, given that this is MS2-based quantification, limited benefit from FAIMS is expected. Have the authors evaluated whether MS1-level quantification would yield different results?

6. Single-cell and PBMC applications
For the reported 230 phosphosites in unenriched single-cell samples, please provide XICs for selected phosphopeptides and show ID score distributions relative to unmodified peptides.
Are the cell sizes shown in Fig. 7b derived from the cellenONE? Given the large cell size variability of the sample, how do these measurements correlate with cell elongation and do the authors manually validate single-cell isolation?
Given the substantial differences in cell size among PBMC cell types, the observed unsupervised clustering may reflect variation in both cell type–specific markers and cell size. It would be informative to perform a similar analysis on the homogeneous HeLa dataset and overlay clustering with measured cell sizes to assess the extent to which cell size contributes to the observed separation.

Minor points
In Fig. 1c, the 250 pg condition without DDM yields nearly 10,000 more precursors than the corresponding 250 pg condition in Fig. 1b. Please clarify the source of this discrepancy.
Throughout the manuscript the authors nicely pair IDs with CV plots, please also amend the CVs for Fig. 1b–c, f and g.
Please explicitly state the flow rate corresponding to the term "high-flow."
In lines 486 onward, the authors suggest that the benefit of DDM primarily arises from improved peptide recovery rather than cell lysis. However, the detergent serves a dual function in cell lysis and peptide recovery, as no alternative lysis reagent

was evaluated this statement needs to be adapted.
Please clarify which acquisition method was used for the FAIMS CV evaluation (Fig. 2d) and the PBMC data (Fig. 7-8).
Please clarify if the reported CV in Fig. 3h is derived from data processed with or without MBR?
Please clarify whether the no-FAIMS methods involved physical removal of the FAIMS unit or disabling FAIMS filtering.
In line 415, the authors refer to “minimally processed” cells. Please clarify which processing steps are included or omitted under this definition.

Version 1:

Reviewer comments:

Reviewer #1

(Remarks to the Author)
I carefully reviewed the revised version of the manuscript and the authors' response to the reviewers. Overall, the authors have addressed all of my major comments. However, a few concerns arose from the revised version.
Primarily, the main claim of the manuscript is the establishment of a narrow mass range of 385–585 Th. However, several results presented in the revised version raise doubts regarding the importance and general applicability of this analytical approach. First, throughout the manuscript, the authors refer to the mass range of 300–1000 Th as the standard. However, in many proteomics studies, the commonly used range is considerably narrower, typically around 400–800 Th. Since the authors did not perform a direct comparison to this commonly used mass range, it is difficult to fully assess the benefit of employing such a narrow window. In fact, the authors state that they used the 400–800 Th range in Figures 5–8, suggesting that the narrower range may not provide a substantial advantage in practice. Second, it appears that the most influential parameter in the entire study is the column inner diameter (i.d.), yet this parameter is only presented in the first figure. One limitation is that the column used is home-made, which may limit the applicability of the results to other mass spectrometry laboratories that primarily rely on commercial columns. A further point of confusion is that the main text focuses on a mass range of 385–585 Th, whereas the supplementary material shifts to a range of 425–625 Th. I could not find an explanation for this change, and clarification would be helpful.
Overall, I still value the contribution of the publication of the optimized details of all running parameters, however, given that the narrow mass range doesn’t seem to provide a major benefit compared to a commonly used method, I doubt that the advancement of this study is sufficient for publication in Nature Communications.

Reviewer #2

(Remarks to the Author)
In the revised version of this manuscript, the authors clearly lay out the impact of their chromatographic methods and include a trap-and-elute workflow that pushes the throughput of most present SCP work. I want to particularly highlight the improved tone of the overall manuscript in relation to previous work. In this context, I want to highlight that their statement in the abstract ‘routinely quantified over 7,000 proteins per individual HeLa cell at over 100 samples-per-day.’, as most of their work yields ~6800 proteins, while impressive, this statement should still be adapted to e.g., "approaching 7,000 proteins at over 100 SPD".
Additionally, I appreciate the clarification regarding the DDM amounts and the details regarding the reduction without alkylation used across experiments in the revised manuscript. However, the manuscript could explicitly state the risk of DDM carryover and impacted chromatographic separation indicated in Figure R3. While the impact is to be expected, many users assume any non-ionic detergent can be added without consequences. The authors’ TIC data clearly illustrate the progressive accumulation across consecutive injections, which is not sufficiently described in the methods or discussion. Please add a concise statement that DDM concentrations above 500 ng (2 µg) per injection were found to impact chromatographic separation under standard operation and that the ~100 ng were found sufficient for the indicated workflow.
Lastly, I find the author's discussion in response to reviewer 1 comment 4 and reviewer 2 comment 5 very interesting and agree that most combinations of acquisition parameters should be tailored for specific use cases. While Supplementary Data 1 gives a great overview of all methods used in this study, most readers would benefit from a comprehensive overview of their methods tested with suggestions by the authors, based on their experience, for which input amount, experimental considerations, or sample preparation these parameters would be optimal (or not recommended).

Version 2:

Reviewer comments:

Reviewer #1

(Remarks to the Author)
In the revised manuscript the authors addressed all my comments. I appreciate their effort to repeat experiments with a commercial column as well as address the comment regarding the isolation windows.
Given these additions, I now find the manuscript suitable for publication.

Open Access This Peer Review File is licensed under a Creative Commons Attribution 4.0 International License, which permits use, sharing, adaptation, distribution and reproduction in any medium or format, as long as you give appropriate credit to the original author(s) and the source, provide a link to the Creative Commons license, and indicate if changes were made.
In cases where reviewers are anonymous, credit should be given to 'Anonymous Referee' and the source.
The images or other third party material in this Peer Review File are included in the article's Creative Commons license, unless indicated otherwise in a credit line to the material. If material is not included in the article's Creative Commons license and your intended use is not permitted by statutory regulation or exceeds the permitted use, you will need to obtain permission directly from the copyright holder.
To view a copy of this license, visit https://creativecommons.org/licenses/by/4.0/

Point-by-point responses to the reviewers' comments

We thank the reviewers for their careful evaluation of our manuscript and for the highly constructive feedback. We have substantially revised the manuscript and performed extensive additional experiments and analyses in response to the reviewers' comments. Collectively, these revisions considerably strengthen the manuscript by expanding the scope of the workflow evaluation, clarifying methodological limitations and positioning relative to prior work, and further validating the robustness and quantitative performance of the approach.

Major additions and revisions include:

- Addition of a new high-throughput workflow analysis (new Figure 6), benchmarking both direct injection and trap-and-elute configurations across acquisition regimes up to 240 samples per day (SPD), including application to single HeLa cells.
- Extensive new quantitative accuracy analyses comparing narrow-range (425-625 m/z) and conventional wide-range (300-1000 m/z) acquisition strategies across a broad dilution series, demonstrating strong quantitative agreement within the intended ultra-low-input operating range (new Supplementary Note 4).
- New chromatographic characterization of the higher-flow pressure-drop methodology, including analyses of peak symmetry, FWHM distributions, and retention-time-dependent peak behavior, demonstrating that the approach does not substantially compromise chromatographic performance (new Supplementary Note 1).
- Expanded analyses and discussion related to phosphopeptide identification confidence, peptide-centric limitations of restricted scan ranges, and benchmarking relative to prior low-input and single-cell proteomics studies.
- Extensive revision of manuscript text throughout to better position the study relative to prior work, moderate novelty claims, clarify methodological scope and limitations, and improve accessibility for a broader readership.

We believe these revisions substantially strengthen the manuscript and address the reviewers' concerns in full.

Nature Communications manuscript
By Hendriks, Buch-Larsen et al.

General response to reviewers:

We have carefully gone through all of the comments made by the reviewers and addressed them point-by-point below. Our answers to the reviewer questions are indicated in **blue text**.

Reviewer #1 (Remarks to the Author):

This study is a joint effort of the Olsen team and Thermo Scientific, introducing Thermo's new instrument Orbitrap Astral Zoom, and specifically, application to very low protein loads in small single cells. The study meticulously examines many of the parameters of the MS and some of the sample preparation and LC methods and provides a valuable roadmap to the utilization of the instrument for single cell proteomics, including analysis of very small cells of 6-8 um. Among the MS examinations, they compare the Astral-Zoom to the previous version of the orbitrap Astral, they examine the use of FAIMS and thoroughly calibrate the mass ranges and window sizes. Overall, the research is very nicely presented and explained, and it will be very useful to the single-cell proteomics experts among the readers. For the broader audience, the manuscript shows the cutting-edge capabilities of single cell proteomics to date, however many of the details will not be understood by readers outside the proteomics field. Overall, I support publication of this study after answering the following comments:

Reply: We thank the reviewer for their thoughtful and supportive assessment of our study. We appreciate their recognition of the careful and systematic examination of MS, LC, and sample preparation parameters, and their view that this work provides a valuable roadmap for the use of the Orbitrap Astral Zoom platform for low-input samples and single-cell proteomics. We are also encouraged that the reviewer finds the manuscript clearly presented and illustrative of the current state-of-the-art in single-cell proteomics.

1. The entire study focuses on proteomic depth, but it mostly analyzes 60 samples per day. Going forward in the field will necessitate increasing the throughput of the single cell measurements. Otherwise, typical experiments of few thousands of cells will take months of measurement. Having such a thorough examination of all the LC-MS parameters is not complete without showing the impact of increased throughput to more than 100 SPD (and even higher).

Reply: We are grateful to the reviewer for this important and constructive suggestion, and we fully agree that increasing analytical throughput is essential for the future scalability of SCP experiments, particularly for studies involving thousands of single cells.

To address this point, we have now performed an extensive additional evaluation of higher-throughput acquisition strategies and incorporated the results of these new experimental data as a new Figure 6 (and as Figure R1 below). Specifically, we investigated how our optimized ultra-sensitive nDIA workflow scaled toward progressively higher-throughput operation using online LC setups with both direct injection (without a pre-column) and a trap-and-elute configuration. The latter enables substantially increased throughput through rapid loading onto a reusable pre-column and is increasingly being incorporated into contemporary high-throughput proteomics workflows (Guzman et al., 2026; Huang et al., 2026; Petrosius et al., 2025; Zheng et al., 2023).

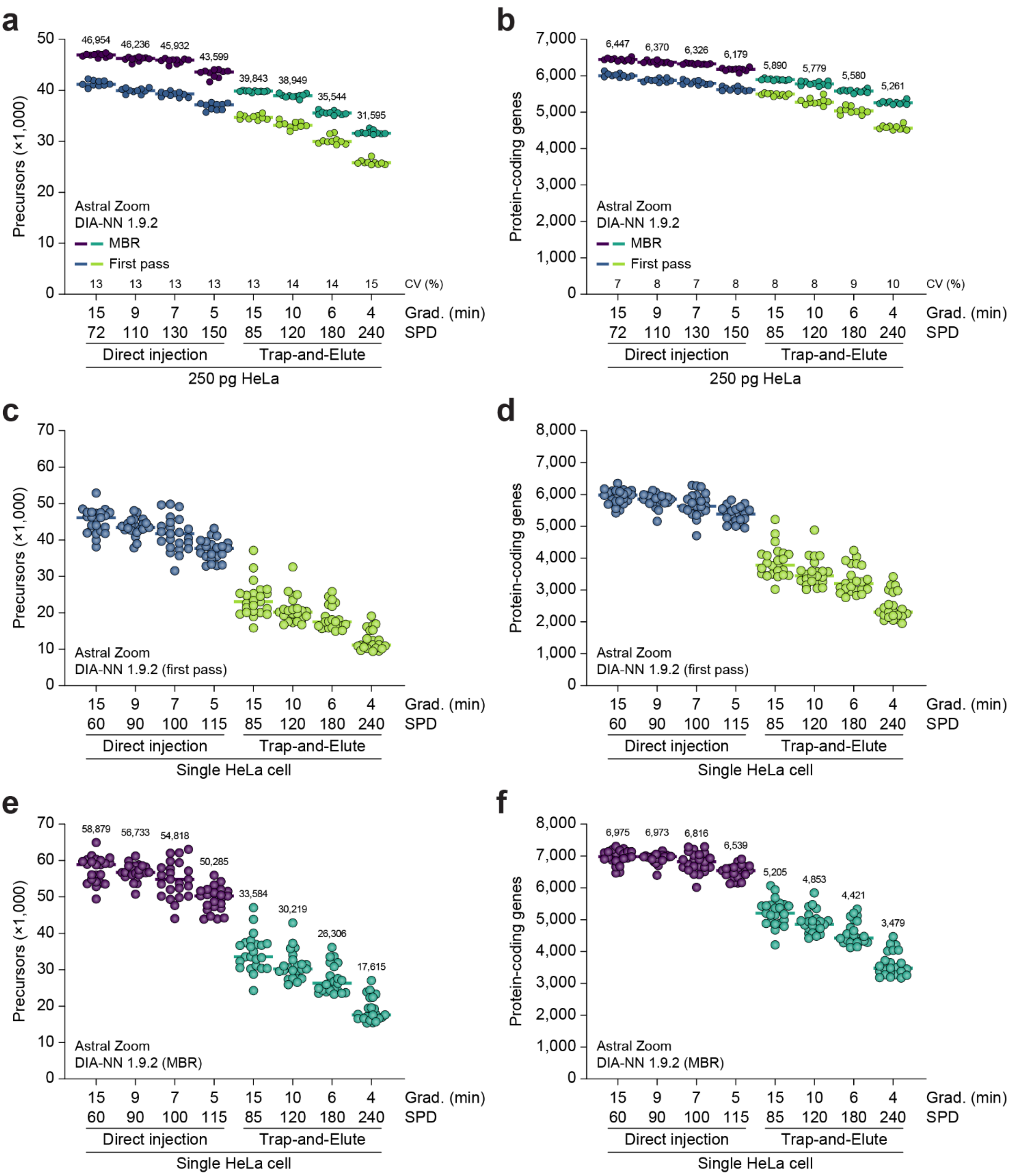

Figure R1 / Figure 6. Deep single-cell proteome coverage is maintained across high-throughput workflows. *(A) Number of precursors identified from 250 pg HeLa lysate using direct injection or trap-and-elute chromatography configurations across progressively higher-throughput workflows. All analyses performed using the Orbitrap Astral Zoom MS, and data analyzed using DIA-NN in either first pass or matching-between-runs (MBR) mode. Bars represent median values, exact medians (with MBR) displayed above each group of data. Numbers above the x-axis represent median coefficient of variation percentage, n = 10 technical replicates. "Grad."; analytical gradient length, "SPD"; samples-per-day. (B) As* ***A**,*

*but for protein-coding genes. (C) As **A**, but for single HeLa cells (median diameter ~26.7 µm), with automated single cell isolation and digestion using the CellenOne. Data analyzed using DIA-NN in first pass mode. n = 23 single cells per condition. (D) As **C**, but for protein-coding genes. Blank injections (n = 2 per method) analyzed alongside the single-cell throughput experiments yielded minimal identifications (0–7 protein-coding genes; see Source Data). (E) As **C**, but data analyzed with DIA-NN in MBR mode and inclusion of a computational 20-cell carrier measured for each method. (F) As **E**, but for protein-coding genes.*

Using low-input HeLa lysate, we demonstrate that direct injection workflows can maintain identification of ~6,200 protein-coding genes from 250 pg of HeLa lysate at throughput up to 150 SPD (5 min gradient), while trap-and-elute enabled throughput up to 240 SPD (4 min gradient) with ~5,300 protein-coding genes identified from 250 pg HeLa. Importantly, we also evaluated these approaches in the context of single HeLa cells. Even under the highest-throughput conditions evaluated here, we could still identify thousands of proteins, including a median of ~6,500 proteins at 115 SPD (5 min gradient) using direct injection, a median of ~4,900 proteins at 120 SPD (10 min gradient) using trap-and-elute, and a median of ~3,500 proteins at 240 SPD (4 min gradient) using trap-and-elute.

We consistently observed that direct injection provided greater proteome depth compared to trap-and-elute under the gradient conditions that we tested in this work, particularly in the context of single-cell analysis. This supports that direct injection on an analytical column generally offers superior peptide chromatography compared to trap-and-elute setups because it eliminates additional sources of band broadening, minimizes sample loss of hydrophilic peptides, and avoids the dilution effects inherent to transferring sample from a trap column to an analytical column. It also highlights a practical tradeoff between analytical throughput and proteome depth, although recent work from the Kelly lab has shown highly robust performance using trap-and-elute setups in the context of low-input analyses (Huang et al., 2026), which we also mention in the manuscript discussion.

We have added the following section to the Results:

"As recent SCP studies have increasingly emphasized higher-throughput acquisition regimes up to 120 SPD (Ye et al., 2025), we next investigated how our optimized ultra-sensitive nDIA workflow scaled toward progressively higher-throughput operation (Fig. 6). We considered both direct injection, the HPLC methodology used so far in this work, and trap-and-elute chromatography configuration, which facilitates substantially higher sample throughput by fast sample loading onto a reusable pre-column (Guzman et al., 2026; Huang et al., 2026; Petrosius et al., 2025; Zheng et al., 2023). For analysis of 250 pg HeLa lysate, direct

injection enabled acquisition at up to 150 SPD by shortening the analytical gradient from 15 to 5 min, while still identifying ~44,000 precursors and ~6,200 protein-coding genes (Fig. 6A-B), or ~93% and ~96% compared to the 72 SPD method, respectively. Using trap-and-elute, throughput could be further increased to 240 SPD using a 4 min analytical gradient (Fig. 6A-B), with ~32,000 precursors and ~5,300 protein-coding genes, or ~79% of precursors and ~89% of protein-coding genes compared to the 85 SPD method. Notably, when using matched 15 min analytical gradients, direct injection consistently yielded higher numbers of precursors and protein-coding genes compared to trap-and-elute acquisition, and even the 5 min direct injection gradient enabled identification of more precursors and protein-coding genes compared to the 15 min trap-and-elute gradient (Fig. 6A-B). Quantitative precision remained robust across all throughput conditions, with median protein CVs below 10% (Fig. 6B).

We next evaluated the same acquisition strategies in the context of single HeLa cells (Fig. 6C-F). Here, due to the larger injection volumes (~4 µL) required for SCP samples when using our workflow, direct injection throughput was modestly reduced compared to peptide dilution experiments, whereas trap-and-elute throughput stayed constant. Nevertheless, using direct injection we were able to identify a median of ~7,000 protein-coding genes from single HeLa cells at 60 SPD, with similar performance maintained at 90 SPD, and still identified ~6,800 and ~6,500 protein-coding genes at 100 and 115 SPD, respectively (Fig. 6C-F). Interestingly, compared to the 250 pg HeLa lysate experiments, trap-and-elute performance deteriorated more substantially in the context of single-cell analysis, with the number of identified protein-coding genes dropping gradually from ~5,200 to ~3,500 per cell when going from 85 SPD to 240 SPD (Fig. 6C-F). Overall, these experiments demonstrate that our SCP workflow can be configured across a broad range of analytical throughput regimes while maintaining substantial proteome depth and quantitative robustness."

We have added the following section to the Discussion, replacing the previous small paragraph mentioning sample throughput:

"*Recent SCP studies investigating HeLa and related cell models have increasingly emphasized the balance between proteome depth and analytical throughput (Bubis et al., 2025; Ye et al., 2025). In this study, our optimized workflow achieved the deepest label-free SCP coverage reported to date while maintaining comparatively high throughput across multiple acquisition regimes (Figure 5). Notably, we found that substantial proteome depth could still be retained at throughput exceeding 100 SPD, both for low-input HeLa lysate and single-cell analysis. In the context of single HeLa cells, direct injection workflows maintained proteome coverage approaching ~7,000 protein-coding genes even at throughput exceeding 100 SPD (Figure 6).*

We further evaluated trap-and-elute chromatography configurations, which are increasingly being incorporated into both high-throughput and low-input proteomics workflows (Guzman et al., 2026; Huang et al., 2026; Petrosius et al., 2025; Zheng et al., 2023). While trap-and-elute enabled throughput up to 240 SPD, we consistently observed reduced proteome depth compared to direct injection under matched gradient conditions, particularly in the context of single-cell analysis. Similar reductions in identifications upon transition from direct injection to trap-and-elute workflows have also been reported previously in low-input proteomics (Zheng et al., 2023), although other recent studies have demonstrated strong overall robustness and flexibility of trap-column configurations for low-input LC-MS workflows (Huang et al., 2026). Importantly, even under the highest-throughput conditions evaluated here (240 SPD), we were still able to identify ~3,500 protein-coding genes from single HeLa cells. Together, these findings suggest that the optimal balance between throughput, loading strategy, and proteome depth remains highly context-dependent and likely varies according to sample amount, chromatographic configuration, and analytical priorities."

Together, we believe that these additional experiments demonstrate that our optimized workflow is adaptable across a wide range of throughput conditions while still enabling deep proteome profiling from low-input and single-cell samples.

2. The use of narrow mass ranges has been previously shown to increase identification and reduce CVs. Nevertheless, this narrowing also has limitations as we are sampling fewer peptides. While the authors claim that they retain proteotypicity, they don't actually show that we don't lose protein level quantitative accuracy, compared to broad MZ ranges. Their analyses examine the CV within a condition, this way showing the precision within each method. However, it is also important to examine the correlation of the protein intensities with the ones obtained in the wider ranges and also higher loads. One has to ensure that the peptides within this range are actually representative of the true protein amounts and that we are not skewing results due to this restriction.

Reply: We appreciate the reviewer's constructive suggestion, and we agree that quantitative precision within a method does not necessarily demonstrate quantitative agreement between different acquisition strategies. To address this concern, we have now performed an extensive additional evaluation comparing our narrow-range 425-625 m/z DIA workflow against a conventional wide-range 300-1000 m/z acquisition strategy across a dilution series ranging from 5 pg to 2500 pg HeLa lysate. These data are now included as Supplementary Note 4 (and as Figure R2 below).

Consistent with the reviewer's expectation, we observed that the broader 300-1000 m/z method identified more precursors at higher sample loads, reflecting the increased peptide sampling achieved by the wider scan range. Nevertheless, when evaluating the agreement in protein abundance measurements between methods, we found very strong concordance across the intended operating range of the narrow-window workflow. In particular, from 25-250 pg – the range for which the ultra-sensitive 425-625 m/z method was designed – regression slopes approached unity (~0.95–0.99), with high linearity (R^2 ~0.98–0.99) and low inter-replicate deviation.

At very low loads (5-25 pg) and very high loads (2500 pg), we observed modest deviation from linearity and mild ratio compression. Interestingly, in both scenarios the narrow-range method slightly underestimated protein abundance relative to the broader scan-range method. However, median absolute deviation analysis demonstrated that these deviations were highly systematic and largely consistent across replicate-to-replicate, cross-load, and cross-method comparisons, suggesting that the increased variance at ultra-low input primarily reflects stochastic sampling effects rather than substantial distortion introduced by restricting the scan range.

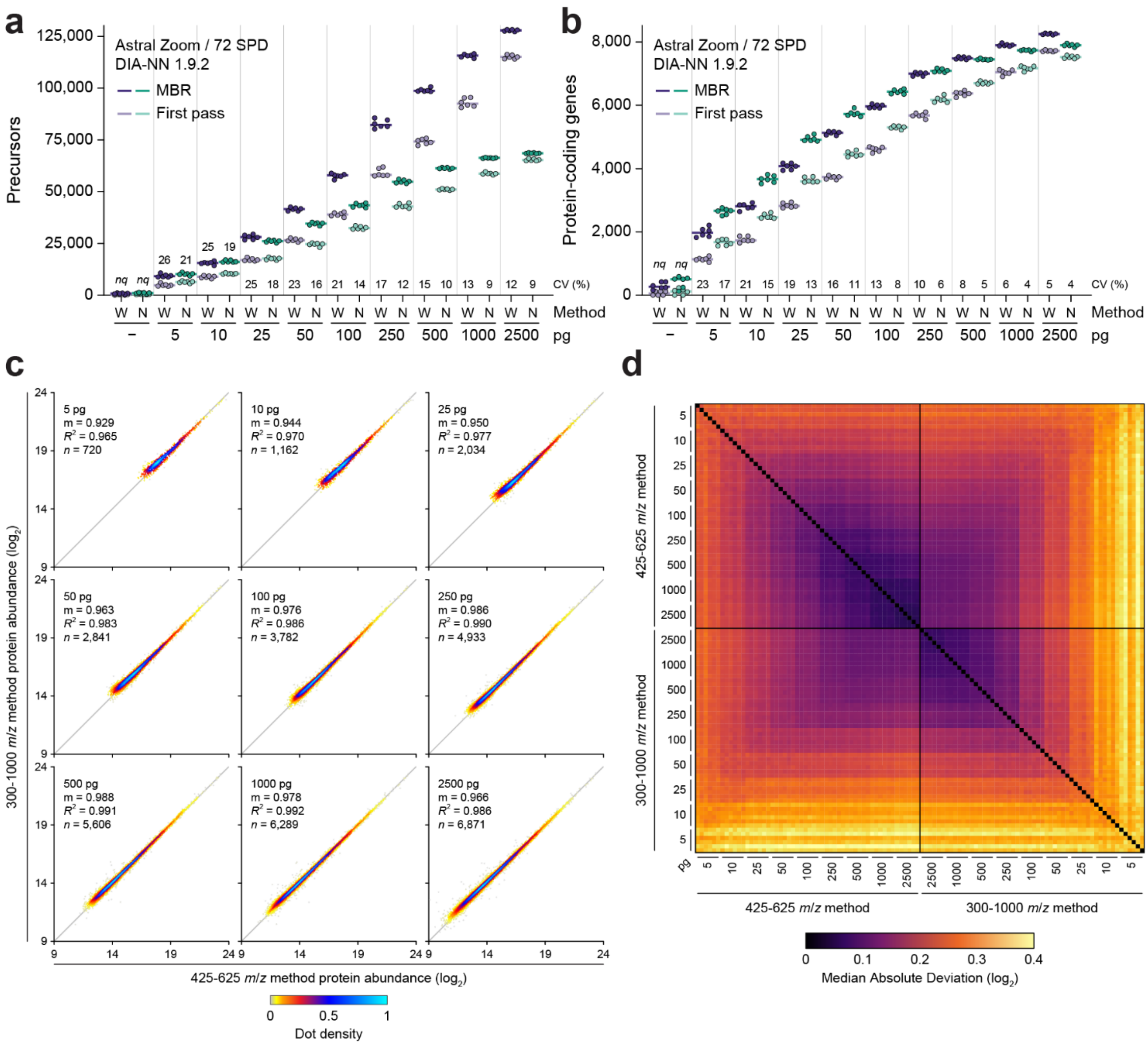

Figure R2 / Supplementary Note 4. Assessment of quantitative accuracy between 300–1000 and 425–625 m/z scan range methods. *(A) Dilution series experiment, ranging from 5 pg to 2500 pg of injected HeLa lysate, comparing a wide scan range of 300–1000 ("W") scan range to a narrow scan range of 425–625 ("N"). All injections were performed in ascending amounts, starting with a blank to ensure negligible sample carry-over. Number of identified precursors are indicated, bars represent median, with data analysis using DIA-NN v1.9.2 in first pass mode and MBR mode, as indicated. Numbers above the x-axis represent median coefficient of variation percentage, n = 6 technical replicates. (B) As* ***A****, but showing protein-coding genes. (C) Scatter plot analysis of median* log_2 *abundance values, based on first pass (no MBR) data, comparing the wide and narrow methods at each different load. Each dot represents one protein-coding gene quantifiable in both methodologies (with n indicated). The grey line is drawn at x=y (i.e. perfect accuracy). The m value indicates slope of linear regression,* R^2 *indicates the squared Pearson correlation. (D) Heatmap analysis, visualizing the median absolute deviation (MAD) between* log_2 *abundance values on a per-replicate basis, and calculated using first pass data.*

Supplementary Note 4 text:

*"To investigate how using a narrow scan range of 425−625 m/z would impact quantitative accuracy, we performed a dilution series experiment wherein we evaluated both the narrow-range method and a wide-range 300−1000 m/z method, with 5 pg of HeLa lysate as the lowest load and 2500 pg as the highest load (**A-B**). We observed that at loads of 50 pg and above, the 300−1000 method identified more precursors, whereas at 2500 pg loads the 300−1000 also identified more protein-coding genes. Notably, when factoring in MBR across all loads, the break-even points were at 25 pg and 500 pg, for precursors and protein-coding genes, respectively. When evaluating agreement in protein abundance measurements between the narrow- and wide-range methods, we found very strong agreement between both methods at loads ranging from 100 to 1000 pg (**C**). At 2500 pg, where the broader scan-range method identifies substantially more peptides, we observed a very modest ratio compression, and similarly at low loads of 5-25 pg we observed a modest reduction in linearity. Interestingly, in both low- and high-load scenarios, the narrow-range method underestimated protein abundance relative to the wide-range method. Reassuringly, when looking at median absolute deviation on a replicate-to-replicate basis, we found that the narrow-range 425−625 method was overall more robust compared to the wide-range method, with median deviation remaining below ~15% even at 5 pg loads (**D**). Notably, the deviation observed at 5 pg was largely consistent across replicate-to-replicate, cross-load, and cross-method comparisons, indicating that the increased variance primarily reflects the stochastic nature of ultra-low-input measurements rather than additional distortion introduced by the narrow scan-range method. Importantly, the intended operating range of 25-250 pg showed the strongest proportionality between methods, with slopes approaching unity and low inter-replicate deviation."*

We believe these additional analyses support that restricting precursor acquisition to 425-625 m/z does not introduce substantial bias in protein-level quantification within the intended low-input operating range of the method, while still enabling the substantial gains in sensitivity and proteome depth described throughout the manuscript.

3. Related to the previous comment, despite the increase in the protein IDs, the authors show an expected reduction in the number of precursors. It should be emphasized that this could be a major drawback if people are interested in peptide-level analyses, such as modified peptides, etc. This should be mentioned in the discussion and also referred to in the section of the phosphopeptide analysis.

Reply: We agree with the reviewer that gaining additional protein identification at the cost of precursors-per-protein is a very important consideration. Indeed, restricting precursor sampling to narrower m/z ranges can increase protein identification depth and quantitative precision, while simultaneously reducing the total number of peptides identified and overall protein sequence coverage. This may impact peptide-centric applications such as characterization of post-translational modifications.

Notably, the phosphoproteomics analysis was done with FAIMS, where we used a 400-800 m/z scan range, which while not as broad as e.g. the commonly used 380-980 m/z method, is also not as restricted as the 425-625 m/z method that we use in the absence of FAIMS.

To address this point, we have amended both the phosphopeptide analysis results section and the discussion.

Updated results sentence:
"*Although we used a partially restricted acquisition range of 400-800 m/z, which may not necessarily be optimal for peptide-level analyses such as phosphopeptide characterization, we were nonetheless able to find a median of 230 localized phosphorylation sites in single HeLa cells (Fig. 5A), which is about twice as high as observed previously.*"

Updated discussion sentence:
"*It should be noted that this comes at a cost of peptides identified and overall sequence coverage, which may impact peptide-centric applications such as characterization of post-translational modifications. Nevertheless, we find that the number of proteins identified was increased, and moreover the quantitative precision was improved for ultra-sensitive nDIA.*"

4. Since the authors include multiple analytical parameters, it could be valuable to add small illustrations to present them. For example, in Figure 1, they can add a small scheme of the MZ window size, mass range, number of windows, MS scans in the different conditions, etc. Similarly, a small cartoon showing the estimated protein amounts from cells of different sizes, instead of showing only diameter and volume. It is not always intuitive to switch between the discussion of protein amounts (in the diluted HeLa) to the single cell and PBMC discussion.

Reply: We thank the reviewer for this suggestion. Figure 1 already included a conceptual schematic of the narrow-window DIA (nDIA) strategy (Figure 1E), illustrating the restricted m/z acquisition range and increased ion injection times used to enhance sensitivity. We have now also annotated Figure 1, Figure S1, and Supplementary Note 1, with additional experimental details in the same style as all the other figures. In addition, all detailed HPLC and mass spectrometry acquisition parameters are provided in Supplementary Data 1, stratified by experiment and figure panel. To make this resource more visible to readers interested in the technical implementation, we now explicitly reference Supplementary Data 1 in the first paragraph of the results section:

"*A comprehensive overview of all LC-MS acquisition parameters for all experiments in this work is provided in Supplementary Data 1.*"

To further improve clarity and better connect the low-input dilution experiments to single-cell applications, we also revised Supplementary Figure 11 to include approximate protein amounts for representative cell types alongside the estimated cell volumes. We agree that this provides a more intuitive link between the analyzed peptide inputs and expected single-cell protein content.

5. What is the difference between Figure 1 plots and Figure S1?

Reply: We apologize for the lack of clarity. We have now annotated Figure 1, Figure S1, and Supplementary Note 1, with additional experimental details in the same style as all the other figures.

To directly answer the question; Figure 1 was based on first pass analysis using DIA-NN, whereas Figure S1 was the same analyses but with Match Between Runs (MBR) enabled. For technical optimization, we prefer to focus on first-pass identifications because these more directly reflect the intrinsic analytical performance of each LC-MS condition, without influence from transfer of identifications between runs or dependence on the composition of the broader dataset.

6. Figure 2C and related figures in the supplementary material should be separated into two graphs. The X-axis should not be doubled in a single graph.

Reply: To improve clarity, we revised Figure 2C and the related Supplementary Figure 3 by visually separating the two acquisition settings into distinct sub-graphs with independent x-axis labeling, and by adding a dividing line between the two m/z sweep series. This preserves the relationship between the paired gas-phase fractionation experiments while making the distinction between acquisition conditions clearer.

Reviewer #2 (Remarks to the Author):

In this manuscript, Hendriks and Buch-Larsen et al. describe a stepwise optimization based on previously published Astral and Astral Zoom methods for single-cell–equivalent inputs and small single-cells. The authors systematically revisit method parameters and demonstrate sensitive detection of phosphorylated peptides at single-cell inputs. While the manuscript is generally well structured and the experiments appear carefully executed, the level of methodological novelty currently does not, in my view, meet the threshold for publication in Nature Communications. My concerns are detailed below.

Reply: We are grateful for the reviewer's careful evaluation of our manuscript, and for recognizing that our study is generally well structured and that the experiments appear carefully executed. We appreciate the reviewer's concerns regarding the degree of methodological novelty, and we have carefully considered these points throughout the revision. In response, we have further clarified the conceptual and technical advances of the workflow, expanded discussion of the scope and limitations of the methodology, and incorporated additional analyses and clarifications as detailed below.

1. Novelty and relation to prior work. The manuscript would benefit from a clearer delineation of what methodological advances are truly novel beyond previous studies, some of them are indicated below: A substantial fraction of the optimization presented in Fig. 2 has already been described in previous publications, including Petrosius, Valdemaras et al., Molecular & Cellular Proteomics (2024), and Hsu et al., Journal of Proteome Research (2025) and their own work Guzman et al., Nature Biotechnology (2024). While the analysis depth of ~3,000 protein-coding genes from small cells is impressive exceeds previous reports, several prior studies have demonstrated deep coverage of small cells (e.g., THP-1 cells ~10 µm; bone marrow cells in work by Specht et al., Genome Biology (2021), Ctortecka et al., Nature Communications (2024) and Furtwängler et al., Science (2025)). Please adapt the tone regarding the novelty of 'small cell analysis' throughout the manuscript.

Reply: We thank the reviewer for this thoughtful perspective and agree that several individual components of the workflow presented here have been explored previously in the context of low-input and single-cell proteomics. These include optimization of FAIMS-assisted acquisition (Bubis et al., 2025; Petrosius et al., 2025), narrow-window DIA strategies (Guzman et al., 2024; Ye et al., 2025), and recent characterization of

the Orbitrap Astral and Astral Zoom platforms in specific analytical settings (Guzman et al., 2026; Hsu et al., 2025).

We have extensively revised the manuscript to more clearly delineate which aspects of our work represent methodological optimization and integration of existing concepts, versus the specific advances demonstrated here. In particular, we have moderated several statements regarding novelty throughout the manuscript and expanded discussion of related prior studies.

Importantly, while individual methodological elements have been reported previously, we believe the present work distinguishes itself through the systematic integration and benchmarking of low-input sample preparation, ultra-sensitive nDIA acquisition, FAIMS-assisted acquisition, chromatography optimization, and Orbitrap Astral Zoom operation into a unified label-free SCP workflow spanning dilution-series experiments, single HeLa cells, and very small primary PBMCs.

Regarding the studies highlighted by the reviewer, we note that the cited Astral Zoom work by Hsu et al. (Hsu et al., 2025) and more recent work by Guzman et al. (Guzman et al., 2026) focused primarily on higher-input proteomics rather than ultra-low-input or single-cell applications, whereas the work by Guzman et al. (Guzman et al., 2024) and Petrosius et al. (Petrosius et al., 2025) were performed on a first-generation Orbitrap Astral (not Orbitrap Astral Zoom) and investigated different subsets of methodological parameters and did not evaluate the combined workflow presented here.

We additionally agree that prior studies (Ctortecka et al., 2024; Furtwangler et al., 2025; Petrosius et al., 2025; Specht et al., 2021) have demonstrated proteomic profiling of relatively small cell types, including monocytes, THP-1 cells, bone marrow populations, and hematopoietic progenitors. Accordingly, we have moderated the tone throughout the manuscript regarding the novelty of small cell analysis. At the same time, we believe the present study substantially extends current capabilities by achieving deep label-free proteome coverage (>4,000 proteins) from very small primary PBMCs without reliance on carrier channels, while maintaining comparatively high throughput and broad immune pathway coverage.

Below is a full list of the changes we made to the manuscript to adapt our tone in line with the reviewer's suggestions.

We have toned down the novelty claims in our abstract, from:

"*surpasses previous limits in depth and reproducibility*"

To:

"*extends current depth and reproducibility*"

We added additional referencing to previous small-cell SCP work:

"*Recent studies have begun extending SCP workflows toward smaller hematopoietic and immune cell populations, although proteome depth and data completeness generally remain lower than for larger epithelial cell models (Ctortecka et al., 2024; Furtwangler et al., 2025; Specht et al., 2021).*"

We have adapted the tone regarding small cell analysis in the introduction, from:

"*Therefore, further improvements in sample preparation and MS sensitivity is required to study smaller cell types such as immune cells by single-cell proteomics.*"

To:

"*Therefore, further improvements in sample preparation and MS sensitivity are still required to achieve deep and reproducible proteome coverage from smaller immune cell types by single-cell proteomics.*"

We have softened language at the start of the Results section introducing the Astral Zoom, from:

"*This instrument boasts several new features*"

To:

"*This instrument incorporates several new features*"

We added additional referencing to work focused on low-input Astral and FAIMS-assisted methodology:

"*Building on recent studies exploring low-input Astral acquisition strategies and FAIMS-assisted SCP workflows (Bubis et al., 2025; Guzman et al., 2024; Petrosius et al., 2025), we systematically evaluated how these capabilities could be integrated and optimized for ultra-low-input DIA proteomics.*"

We have toned down claims at the end of Results section for deep HeLa SCP, from:

"*achieving unparalleled coverage and precision from individual HeLa cells*"

To:

"*achieving among the deepest label-free coverage from individual HeLa cells reported to date*"

We have softened language at the start of the PBMC Results section, from:

"*Small cells below a certain size (< ~10 µm in diameter) are frequently excluded from SCP analyses. This is because their low protein content leads to insufficient proteome coverage*"

To:

"*Very small cells (< ~10 µm in diameter) remain comparatively underrepresented in SCP analyses. This is because their low protein content often results in reduced proteome depth and data completeness*"

We have toned down the introductory statement for the PBMC Results section, from:

"*This has made robust proteome analysis technically unfeasible for very small cells, and their inclusion can introduce significant noise and missing data into SCP datasets.*"

To:

"*This has historically made deep and reproducible proteome profiling of very small cells considerably more challenging.*"

We have softened language further down the PBMC Results section, from:

"*Contemporary proteomics study of PBMCs relies on bulk analysis*"

To:

"*Most contemporary proteomics studies of PBMCs still rely on bulk analysis*"

Additionally, we have softened and clarified:

"*Here, to evaluate and benchmark our enhanced SCP methodology in the context of very small cells*"

To:

"*To evaluate our optimized SCP workflow in the context of very small primary immune cells*"

We have toned down language further down the PBMC Results section, from:

"*Strikingly, compared to the majority of bulk PBMC studies, we identified as many or more proteins from single PBMCs.*"

To:

"*Notably, despite using single-cell inputs, the depth of proteome coverage achieved here approached or exceeded that reported in several bulk PBMC proteomics studies.*"

Additionally, we have softened:

"*demonstrating the exquisite sensitivity of our methodology*"

To:

"*demonstrating the sensitivity achievable with our optimized workflow*"

We have greatly reduced exaggerated language in the final paragraph of the PBMC results section, from:

"*By pushing SCP sensitivity to its limits, we achieved comprehensive proteome profiling of individual PBMCs – immune cells nearly 100 times smaller in volume than HeLa cells – at throughput and depth rivaling bulk studies. Our approach uncovered thousands of immune-associated proteins, captured comprehensive functional pathway coverage, and resolved distinct immune cell populations based purely on proteomic signatures. These findings establish a foundation for high-resolution mapping of immune heterogeneity directly from patient-derived samples.*"

To:

"*Using our optimized SCP workflow, we achieved deep label-free proteome profiling of individual PBMCs, including very small immune cells with substantially (~50-fold) lower protein content than HeLa cells. Despite the limited cellular material, our workflow enabled broad coverage of immune-associated proteins and pathways, while maintaining relatively high analytical throughput. Furthermore, the proteomic data resolved distinct immune cell populations directly from heterogeneous PBMC samples without prior cellular labeling or sorting. Together, these findings demonstrate the applicability of our optimized workflow to challenging primary immune cell populations and support its utility for high-resolution profiling of immune heterogeneity from patient-derived samples*."

We added additional referencing and clarification at the start of the Discussion section:

"*While several individual aspects of low-input and SCP methodology have previously been explored independently (Bubis et al., 2025; Ctortecka et al., 2024; Furtwangler et al., 2025; Petrosius et al., 2025; Specht et al., 2021; Ye et al., 2025), our goal here was to benchmark and integrate these advances into a unified ultra-sensitive label-free SCP workflow.*"

We have clarified the following statement in the Discussion section, from:

"*These empirical gains align with what has been observed when using the Astral Zoom in the context of high-load and high-throughput proteomics (Guzman et al., 2026), highlighting that the instrument provides benefits across all proteomics applications.*"

To:

"*These empirical gains align with observations from recent Astral Zoom studies performed in higher-input and high-throughput proteomics settings (Guzman et al., 2026; Hsu et al., 2025), while extending evaluation of the platform into ultra-low-input and single-cell-relevant acquisition regimes.*"

We added additional referencing and discussion in the Discussion section:

"*While previous studies have demonstrated SCP analysis of hematopoietic and immune-related cell populations (Ctortecka et al., 2024; Furtwangler et al., 2025; Specht et al., 2021), these approaches frequently relied on booster-proteome carrier-based multiplexing strategies, longer acquisition times, or achieved lower proteome depth from very small cells.*"

We have clarified the following statement in the Discussion section, from:

"*Notably, we did not use computational or experimental carriers to increase identification numbers.*"

To:

"*Importantly, our measurements were obtained using a fully label-free workflow without reliance on experimental or computational carrier channels to boost identifications.*"

Finally, we have removed or rephrased any mention of "comprehensive", when it was in relation to our own results.

Taken together, we have revised the manuscript to better position this work as a systematic optimization and integration study that advances the achievable balance between sensitivity, depth, throughput, and applicability to very small primary cells.

2. Sample preparation

In Fig. 1b, the impact of DDM on adsorptive losses is evaluated using two different column IDs. While the conclusion that the 50 µm I.D. column performs better is reasonable, the use of 5 µg DDM appears excessive. Most low-input or single-cell proteomics studies use ~0.1% DDM during lysis at very low volumes, corresponding to ~500 ng DDM. Additionally, the subsequent comparison of DDM concentrations in Fig. 1c does not include the originally used 5 µg condition but lower, more widely used concentrations. It is unclear why this concentration was excluded, or alternatively why the experiments in Fig. 1b were performed with such a high amount of DDM. Moreover, the lack of difference between 200 ng and 1 µg DDM is unsurprising. It is encouraging that reduction and alkylation can be performed without introducing substantial chemical noise to single cell input samples. Please clarify why this optimized sample preparation protocol was subsequently not applied to the PBMC samples.

Reply: We thank the reviewer for this important comment. We apologize for the inaccurate DDM amounts reported in the original version of the manuscript. After carefully reviewing our data and laboratory logbooks, we have updated the amounts of DDM used to 2 µg in Figure 1B, and 100 ng and 500 ng in Figure 1C.

The initial experiment in Figure 1B was performed with 1 µL of 0.2% DDM, as that is a typical concentration used in the mastermix (Ye et al., 2025). During these experiments, we observed a large increase of hydrophobic signal towards the end of the gradient, which further accumulated with additional back-to-back runs (Figure R3). This late-gradient signal was consistent with accumulation of highly hydrophobic species, likely dominated by DDM-related signal. The increase of signal across additional runs suggested our setup was not able to clear this amount of DDM from the system during standard operation, leading to DDM accumulation in subsequent runs.

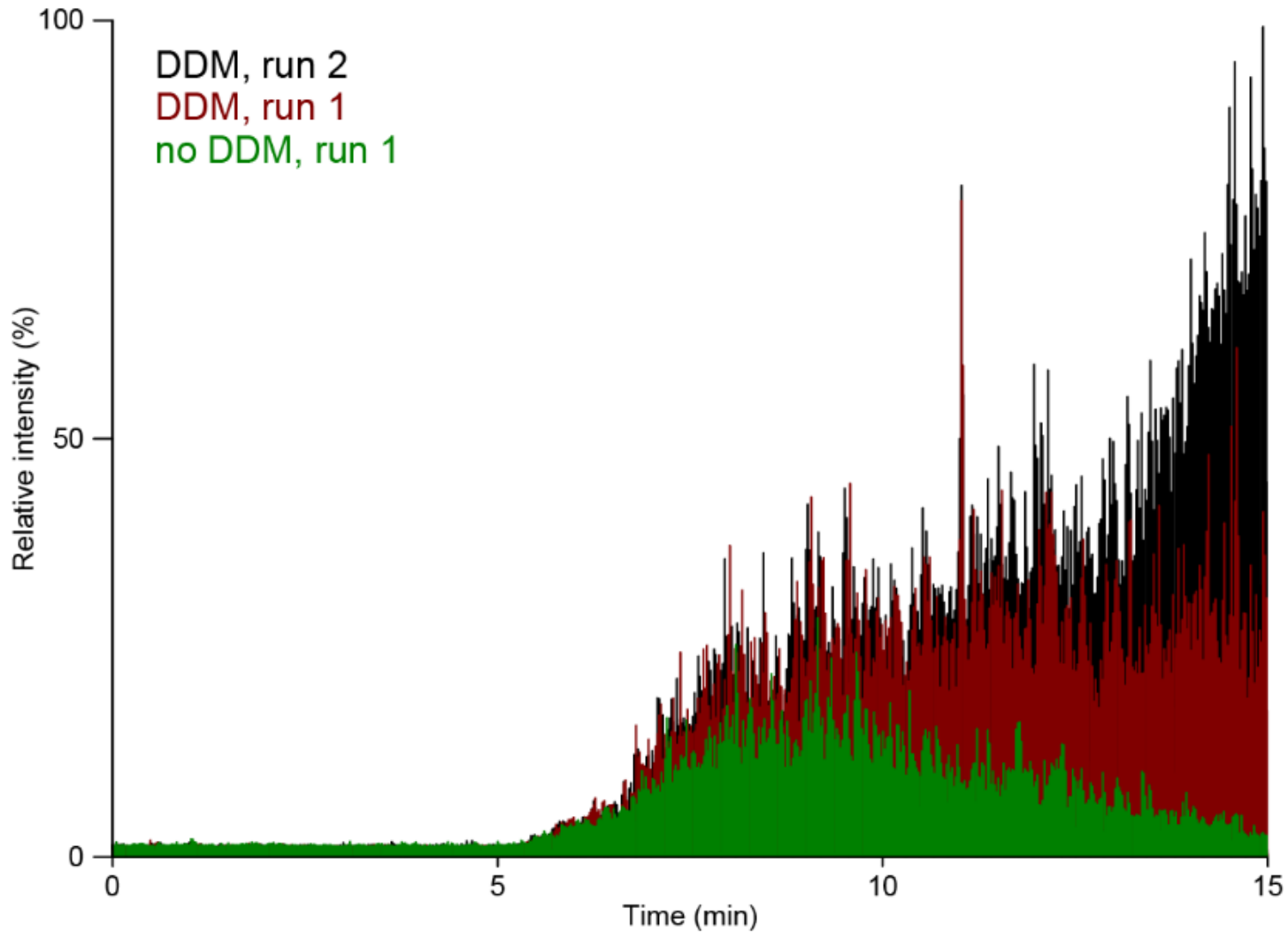

Figure R3. *Representative total ion current chromatograms illustrating accumulation of hydrophobic signal following repeated injections of high amounts of DDM. Shown are consecutive injections containing 2 µg DDM ("DDM, run 1" and "DDM, run 2") compared to a control injection without DDM.*

Thus, we performed the follow-up experiments in Figure 1C, where we diluted the DDM by 4-fold and 20-fold. Already with the 4-fold dilution (500 ng DDM per run), we no longer observed as much DDM signal, and furthermore there was no visible accumulation of DDM on the column when performing several back-to-back runs (without separate column washing programs).

Notably, for the large majority of lysate-based experiments in the manuscript, we inject 100 ng of DDM per run (2 µl injections of sample containing 0.005% DDM). For all main single-cell experiments (Figures 5-8), we use a modified mastermix containing 0.05% DDM, of which approximately 750 nl is dispensed per well, resulting in a total amount of 375 ng of DDM per single-cell injection. Importantly, this is lower than the amount of DDM (500 ng) we determined to be well tolerated by our HPLC and column setup.

We agree with the reviewer that even the smaller amounts of DDM that we tested (100 ng) would already be sufficient (at least in the context of lysate analysis), as this would still constitute a ~1,000-fold molar excess over e.g. 250 pg of HeLa lysate.

With regard to reduction and alkylation, we note that our single-cell methodology only introduces reduction into the workflow (via addition of DTT), but does not alkylate the peptides. We have so far not been able to find an alkylating agent that is compatible with our ultra-low-input workflow. Furthermore, as the single cells are lysed and digested in the presence of fresh DTT, and afterwards acidified, all peptides retain their reduced

state. For data processing, we search for unmodified cysteine residues (free sulfhydryl), rather than carbamidomethylated cysteine residues. We observe similar rates of identification of cysteine-bearing peptides in this manner (Figure 4G and Supplementary Figure 8F), compared to lysate-based workflows that both reduce and alkylate.

Finally, we would also like to clarify that all PBMC samples were processed using the optimized workflow, which entails reduction using DTT (but no alkylation).

3. Peak shapes

For the faster column loading and equilibration where pressure is rapidly reduced from 1000 to 250 bar, does this result in any observable peak broadening in comparison to methods at constant flow? Even at 'high-flow', the reported 4 µL sample volume for single cell samples requires at least ~2 minutes to load (with 500 µL) onto the column, but the active peptide elution time is <15 minutes (Fig. 3c). This appears relatively long and may compromise peak sharpness, please elaborate on FWHM and peak shape for the different chromatographic setups.

Reply: We acknowledge that chromatographic performance is of exceptional importance when designing LC-MS methodology, and during method development we already performed rudimentary analysis of peak properties to ensure acceptable performance. Notably, during initial preliminary testing we found that very rapid pressure drops (over just a few seconds) resulted in very large fluctuations in elution profiles and chromatographic 'splitting' of elution into two parts. In the final "Higher Flow" method used throughout the manuscript, pressure is gradually reduced during the first minute of the gradient. In that manner, the HPLC system remains highly pressurized during column equilibration, sample loading, and gradient initiation, which minimizes time lost to pressure ramping between steps. Importantly, column backpressure stabilizes almost entirely prior to the main peptide elution window (Figure R4), thereby minimizing chromatographic perturbation during active peptide separation. Although the loading phase itself remains relatively long for direct-injection SCP samples, the pressure-drop strategy substantially reduces non-productive overhead while preserving a comparatively long active peptide elution window.

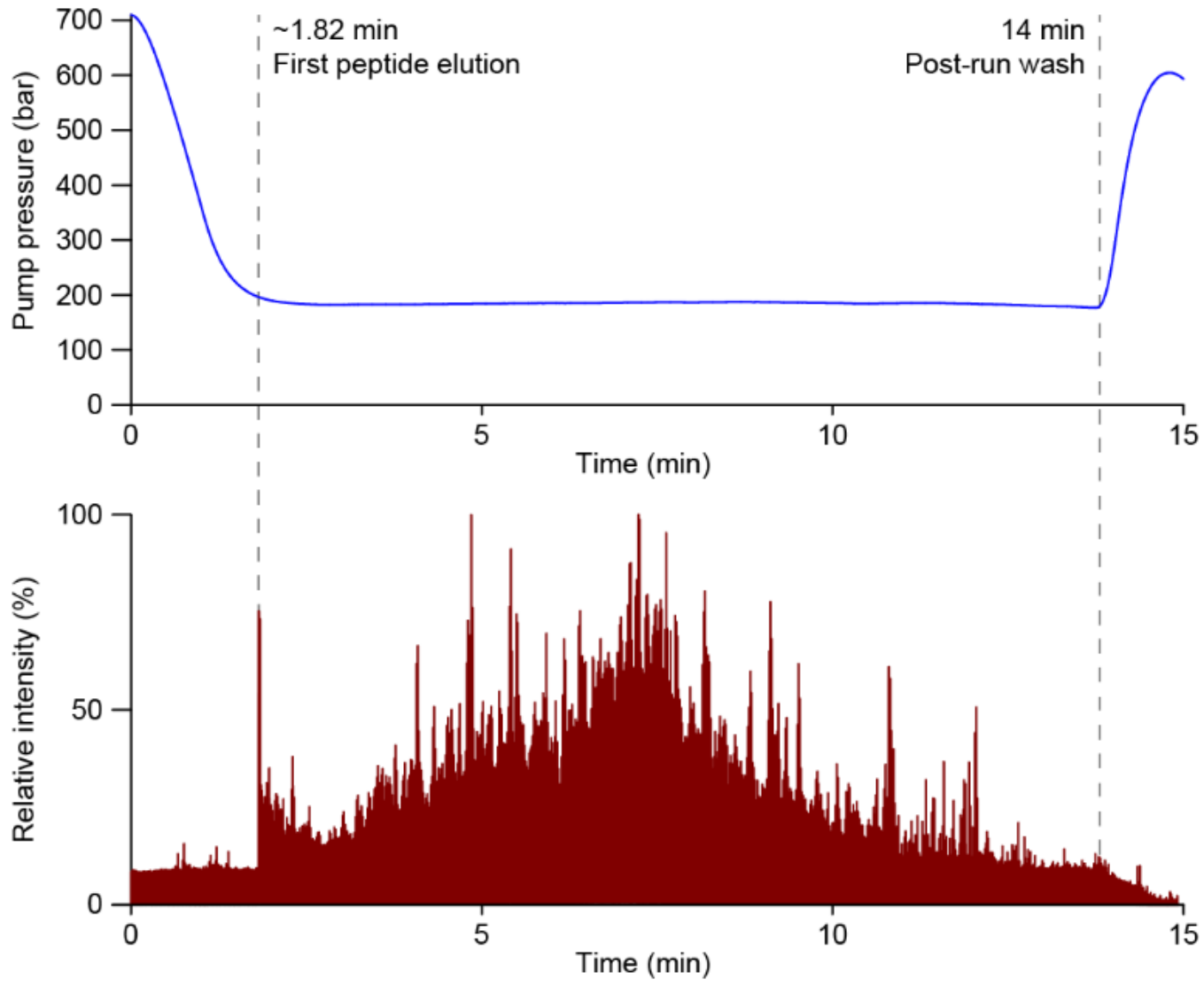

Figure R4. *Representative pump pressure profile and corresponding peptide elution profile for the "Higher Flow" method employing gradual pressure reduction during the first minute of acquisition. Pressure decreases rapidly prior to the main peptide elution window, enabling reduced column delay while minimizing chromatographic perturbation during active peptide separation.*

At the reviewer's request, we have now performed a more elaborate investigation of peak shape and FWHM profiles between the constant flow method and the "Higher Flow" method in which pressure is reduced during the first minute of acquisition and prior to peptide elution, and included this in the revised version of the manuscript (Supplementary Note 1 and Figure R5).

We investigated peak shape, specifically looking at peak symmetry and whether any fronting or tailing was introduced in the "Higher Flow" method. From this analysis (Fig. R5A), we could not observe any deviations in peak shape compared to standard constant-flow methods. Next, we looked at FWHM globally, and found a median of ~2.4 seconds for both constant-flow and higher-flow methods (Fig. R5B), with no notable differences between the overall FWHM distribution.

When investigating FWHM as a function of retention time (Fig. R5C), we did observe that the pressure-drop method results in a fluctuation of peak FWHM, visible as an oscillation between ~2.2 and ~2.6 sec median FWHM as the gradient progresses. We believe this could be a result of the pre-compressed gradient depressurizing in the HPLC and sample line before fully stabilized constant-flow conditions are established, which may transiently influence effective gradient formation and peptide elution behavior. However, we found that the overall effect is relatively minor and the median FWHM does not markedly increase compared to constant-flow methods. Notably, in this specific comparison the active gradient (defined as elution time

between 5th and 95th percentile of identifications) was also somewhat longer for the pressure-drop method (~8 min) compared to the constant-flow method (~7 min), and thus slightly broader peaks are to be expected. Overall, the oscillation we see in the pressure-drop method did not negatively impact identification rates, and observed q-values for identified proteins did not fluctuate as a function of retention time (Figure 1G and R5C). Importantly, the observed oscillations were highly consistent across replicate runs (Fig. R5D), allowing robust chromatographic alignment and consistent downstream analysis by DIA-NN and Spectronaut.

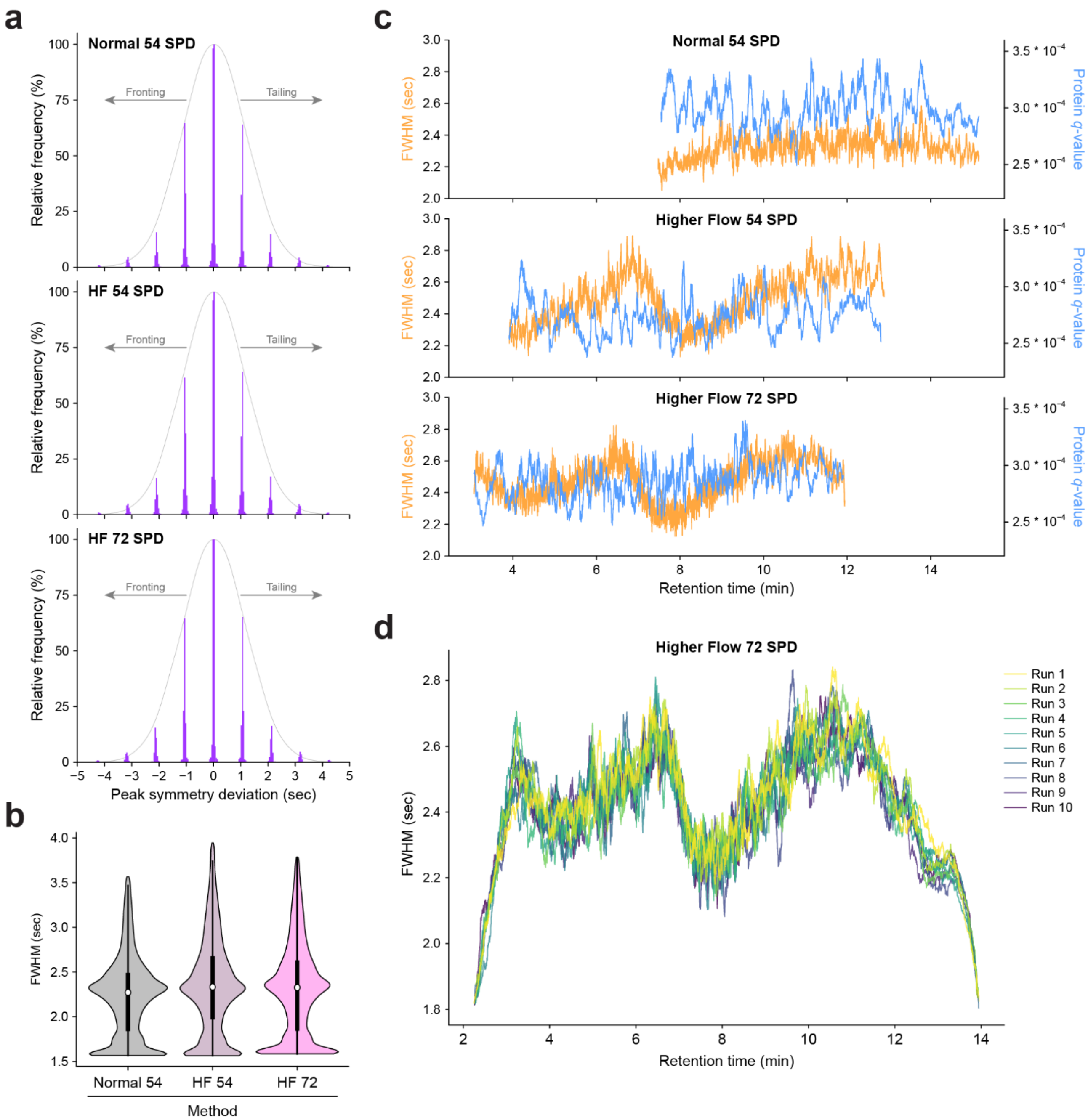

Figure R5 / Supplementary Note 1. Analysis of peak shape and width in the context of higher-flow methods with variable pressure. *(A) Representation of peak symmetry deviation, via determination of the peak apex relative to the center of the retention length. A normal distribution (in grey) was superimposed on the data. Underlying LC-MS data are detailed in* ***Figure 1G and Supplementary Figure 1J-L****. Peak analysis was performed on first pass DIA-NN output. n = 6-10 technical replicates. "Normal"; standard steady-flow chromatography, "HF"; method employing Higher Flow (i.e. 500 nL/min) at the start and end of the gradient to maximize effective peptide elution time, "SPD"; samples per day. (B) As* ***A****, but visualization the distribution of Full Width Half Maximum (FWHM) retention time for all precursors identified within the 5th and 95th percentile of identifications across the gradient. Circle; median, thick line limits; 1st and 3rd quartile, thin line limits; 5th and 95th percentile. (C) As* ***B****, but displaying a 300-precursor running average FWHM (in orange) and*

*corresponding protein q-values (in blue) as a function of retention time, limited within the 5th and 95th percentile of identifications across the gradient. (D) As **C**, but visualizing FWHM for all precursors across the entire gradient on a per-run basis, n = 10 technical replicates.*

Supplementary Note 1 text:

*"To investigate whether the higher-flow methodology employing rapid pressure reduction from ~1000 to ~250 bar impacted chromatographic peak quality, we performed a comparative analysis of peak symmetry and Full Width Half Maximum (FWHM) profiles between constant-flow and higher-flow methods (**A-D**). Peak symmetry distributions approximated a normal distribution centered near zero (**A**), and we did not observe notable increases in either peak fronting or peak tailing for the higher-flow methods compared to standard constant-flow chromatography. Likewise, precursor FWHM distributions were highly similar between methods (**B**), with median FWHM values of ~2.4 sec observed across all conditions.*

*When visualizing FWHM as a function of retention time (**C**), the higher-flow methods displayed a reproducible oscillatory fluctuation in FWHM throughout the active peptide elution window, with median FWHM periodically varying between approximately ~2.2 and ~2.6 sec. We speculate that this behavior reflects gradual depressurization of the pre-compressed gradient prior to establishment of lower-flow analytical conditions. Importantly, however, these oscillations did not result in substantial peak broadening overall, nor did they negatively impact identification confidence, as protein q-values remained stable throughout the gradient (**C**).*

*Finally, the oscillatory FWHM pattern observed for the higher-flow 72 SPD method was highly reproducible across technical replicates (**D**), likely facilitating robust chromatographic alignment and quantitative analysis despite the dynamic pressure transition. Collectively, these analyses demonstrate that the higher-flow methodology does not substantially compromise chromatographic peak shape or width relative to standard constant-flow operation, while enabling reduced column delay and increased effective peptide elution time."*

Overall, while the higher-flow methodology introduces modest and reproducible variation in chromatographic behavior, we do not observe substantial degradation of peak shape, peak width, or identification confidence relative to standard constant-flow operation.

4. Identification variability, and quantitative performance

Was the data shown in Fig. 1b analyzed separately from the other experiments? It is unclear how there is only a marginal decrease in identifications when reducing input from 250 pg to 125 pg.

Reply: In Figure 1B (which is in the absence of MBR analysis), from 250 pg (+DDM), we identified an average of 4,847 protein-coding genes from 45,432 precursors. From 125 pg (+DDM), we identified an average of 4,119 protein-coding genes from 34,703 precursors. This corresponds to ~85% of protein-coding genes and ~76% of precursors identified from 125 pg compared to 250 pg. This decrease follows the typical trend we also observed from the dilution series in e.g. Figure 3A, where reduction of load from 100 pg to 50 pg resulted in 4,455 and 3,759 protein-coding genes and 26,315 and 20,421 precursors identified, respectively (no MBR). In that case, we identified ~84% of protein-coding genes and ~78% of precursors from 50 pg compared to 100 pg. Only at the very limit of detection do we observe sharper drops when halving sample loads. We believe that this relatively marginal drop in IDs in the range of 5 to 250 pg likely reflects that the methodology was specifically optimized for sensitivity within this low-input range. For higher loads (e.g. 2500 pg and above), faster-scanning methods covering broader m/z ranges will be superior (see also Figure R2 or Supplementary Note 4).

MS1 vs. MS2 quantification is only shown down to 50 pg input, which is relatively high compared to other experiments detailed in the manuscript. Please justify this choice and elaborate on the high CVs of their 'standard' MS2 level quantification at 50pg input.

Reply: The MS1 vs. MS2 precision comparison was included as a focused benchmark of quantification behavior under a specific FAIMS-enabled acquisition regime, rather than as a complete low-input dilution series. For this comparison, 50 pg represented the lowest input level in the dataset available for the side-by-side evaluation of MS1- and MS2-based quantification under matched acquisition conditions. Extending this particular comparison to lower inputs would not have been directly comparable, because the MS1-optimized workflow uses substantially longer MS1 accumulation times (100 ms vs 25 ms) and more frequently scheduled MS1 scans (every 0.6 s rather than every ~1 s) compared to the MS2-optimized workflow used throughout the rest of our study.

Regarding precision, the median CV of ~9% for the standard MS2 quantification at 50 pg is modest for low-input proteomics and remains well within an acceptable range. It is somewhat higher than the ~6% achieved

with the dedicated MS1-optimized workflow, which is expected given that our MS2-centric acquisition strategy prioritizes DIA duty cycle and identification depth rather than maximal MS1 precision.

Based on the presented data, one could conclude that MS1-based quantification on the Astral may be beneficial for single-cell or low-input analyses. Why was this strategy not evaluated on the true single-cell datasets?

Reply: We appreciate the reviewer's insightful suggestion, and we agree that the highly precise MS1-based quantification observed in the low-input HeLa dilution experiments raises the question of whether such an approach may also benefit true single-cell proteomics datasets.

To investigate this, we compared MS1- and MS2-based quantification across our own single-cell dataset as well as several previously published SCP datasets (Bubis et al., 2025; Ye et al., 2025); i.e. the same SCP datasets we already included in identification comparison in the initial submission of the manuscript. Notably, the methodology used in previously published SCP work was more focused on MS1-based quant, with 60 ms or 100 ms injection times and MS1 scans every 0.6 sec (Bubis et al., 2025), or 30 ms injection times and MS1 scans every 0.6 sec (Ye et al., 2025).

While MS1-based quantification performed very well in controlled low-input technical dilution experiments (Figure 3H), we consistently observed equal or improved quantitative precision using MS2-based quantification across all evaluated single-cell datasets, both in our data and in re-analyses of published studies (Fig. R6).

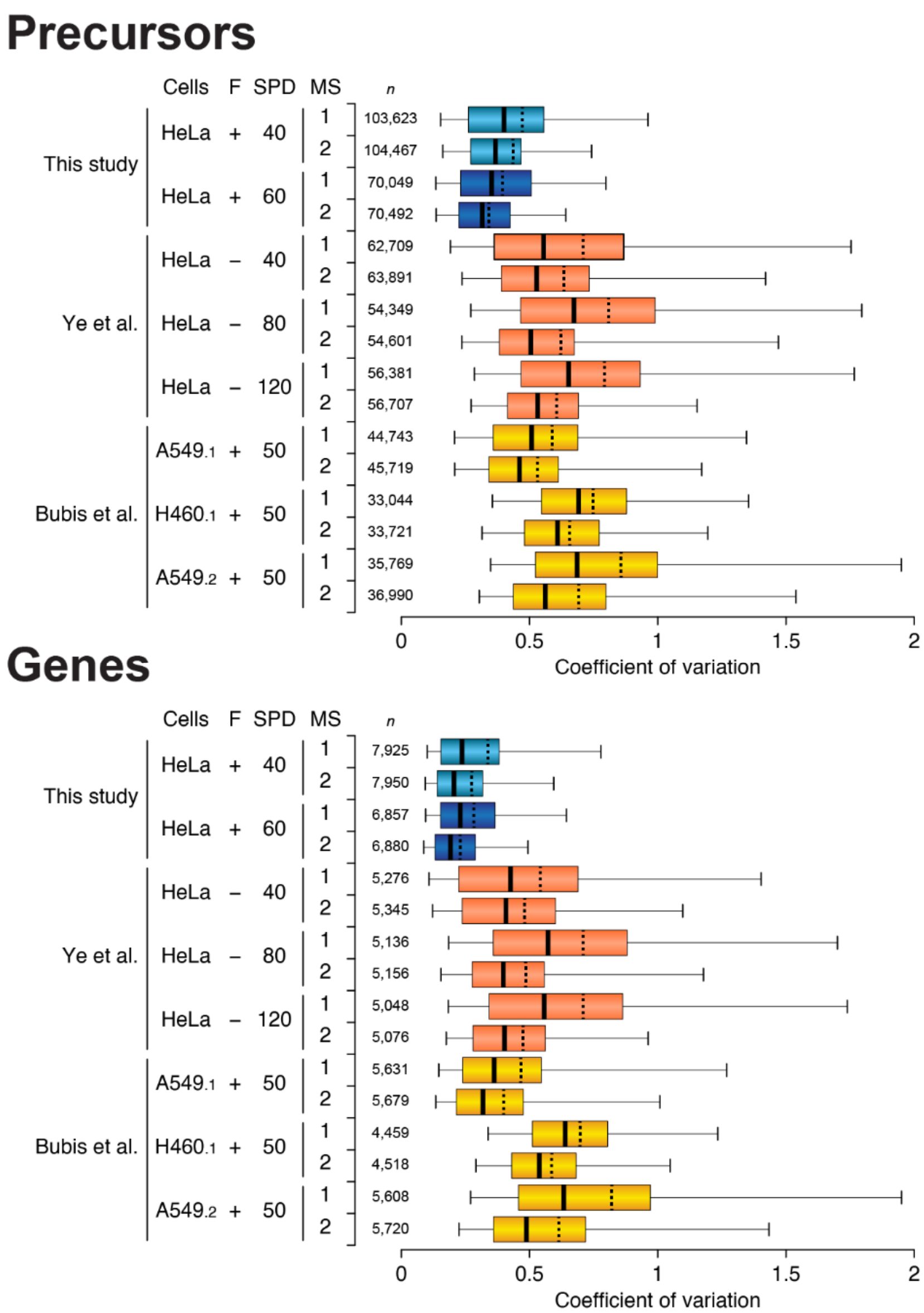

Figure R6. *Comparison of MS1- and MS2-based quantitative precision across single-cell proteomics datasets from this study and previously published SCP workflows (Bubis et al., 2025; Ye et al., 2025). Publicly available raw files were reprocessed together with data from this study using Spectronaut in Method Evaluation mode (see* ***Figure 5C-F****). Boxplots display precursor-level (top) and protein-level (bottom) coefficient of variation distributions for MS1-based quantification ("1") and MS2-based quantification ("2"). Thick line; median, dashed line; average, box limits; 1st and 3rd quartile, whisker limits; 5th and 95th percentile. The total number of quantified precursors or protein-coding genes (n) is indicated for each dataset.*

We speculate that MS1-based quantification may benefit particularly from the highly homogeneous nature of technical HeLa dilution series, where chromatographic reproducibility and feature alignment and normalization can be highly optimized under controlled technical conditions. In contrast, real-life single-cell datasets contain true biological cell-to-cell heterogeneity and sample preparation variability, potentially

reducing the relative advantage of MS1-based quantification. Under such conditions, MS2 fragment ion traces may provide more robust quantitative estimates.

Importantly, we do not view these approaches as mutually exclusive, and the relative advantages of MS1- versus MS2-based quantification may depend on the biological and analytical context. As the present manuscript is primarily focused on development and application of ultra-sensitive DIA methodology rather than a systematic comparison of MS1- and MS2-based quantification strategies, we prefer not to expand this discussion extensively within the main manuscript. We believe that rigorous benchmarking of the relative strengths and limitations of these quantification approaches across different SCP workflows and biological contexts would warrant a more dedicated future study.

5. Acquisition method design

The authors demonstrate that the new ultra-sensitive nDIA methods yield more identifications than the standard nDIA approach. However, especially in combination with FAIMS smaller isolation windows appear to increase CVs without providing a clear benefit in identifications (Fig. 2e). This seems consistent with prior observations (e.g., Bubis et al., Nature Methods, 2025) suggesting that larger isolation windows combined with longer injection times may be more appropriate for ultra-low input samples. Please elaborate on the benefits of nDIA in comparison to wider isolation window methods especially in the context of single-cell. In Fig. S8i, given that this is MS2-based quantification, limited benefit from FAIMS is expected. Have the authors evaluated whether MS1-level quantification would yield different results?

Reply: We thank the reviewer for these insightful comments. We agree that broader isolation windows increase ion transmission and ion population per scan, which can improve quantitative precision, particularly for MS2-based quantification at ultra-low input levels. This consideration strongly influenced the acquisition strategies used throughout the study.

In the absence of FAIMS, we found that narrower isolation windows remained advantageous, as there is no filtering to reduce singly-charged atmospheric and background contaminants (Figure 3C). In the absence of FAIMS, and in the context of a 300-1000 m/z scanning method, the 6 Th / 7.5 ms method resulted in reduced identification performance without notable improvement in quantitative precision (Fig. 1F), compared to the 4 Th / 5 ms method. In contrast, when FAIMS was enabled, the reduction in chemical background allows for the use of substantially broader isolation windows (20 Th) in combination with longer ion injection times,

which became the primary strategy employed throughout the single-cell analyses in Figures 5-8. Indeed, this configuration (FAIMS, 20 Th, 40 ms, 400-800 m/z) yielded precise MS2-based quantification in our dilution series experiment (Figure 3A), on par with the no-FAIMS method (4 Th, 17.5 ms, 425-625 m/z).

We therefore view the optimal acquisition strategy as context-dependent. In the absence of FAIMS, narrower DIA windows provide a favorable balance between selectivity and sensitivity. With FAIMS-enabled acquisition, however, broader isolation windows and extended injection times become feasible and advantageous, consistent with recent observations (Bubis et al., 2025). In our dilution series experiments (Figure 3A), the FAIMS-enabled workflow increasingly improved proteome coverage at input levels of 50 pg and below. For single HeLa cells (Figure 4H), both FAIMS and no-FAIMS approaches performed comparably, which is consistent with the relatively high (~250 pg) protein content of HeLa cells. In contrast, for substantially smaller PBMCs (Fig. 7 and Fig. S11B), where estimated protein content is considerably lower (~5-40 pg), the FAIMS-enabled workflow provided clear benefits. We therefore believe that the relative advantages of narrower versus broader isolation window strategies depend strongly on the effective protein input and overall sample complexity of the analyzed cells.

Regarding the reviewer’s question about MS1-level quantification in Figure S8I (related to Figure 4H), we performed additional MS1-based quantification analyses for the same single-cell dataset following the reviewer’s suggestion (Figure R7). Interestingly, while highly optimized MS1-based quantification performed very well in technical HeLa dilution experiments (Figure 3H), we did not observe improved quantitative precision relative to MS2-based quantification for this single-cell dataset (Figure S8I), nor did we observe a substantial difference between FAIMS and noFAIMS acquisition at the MS1 level.

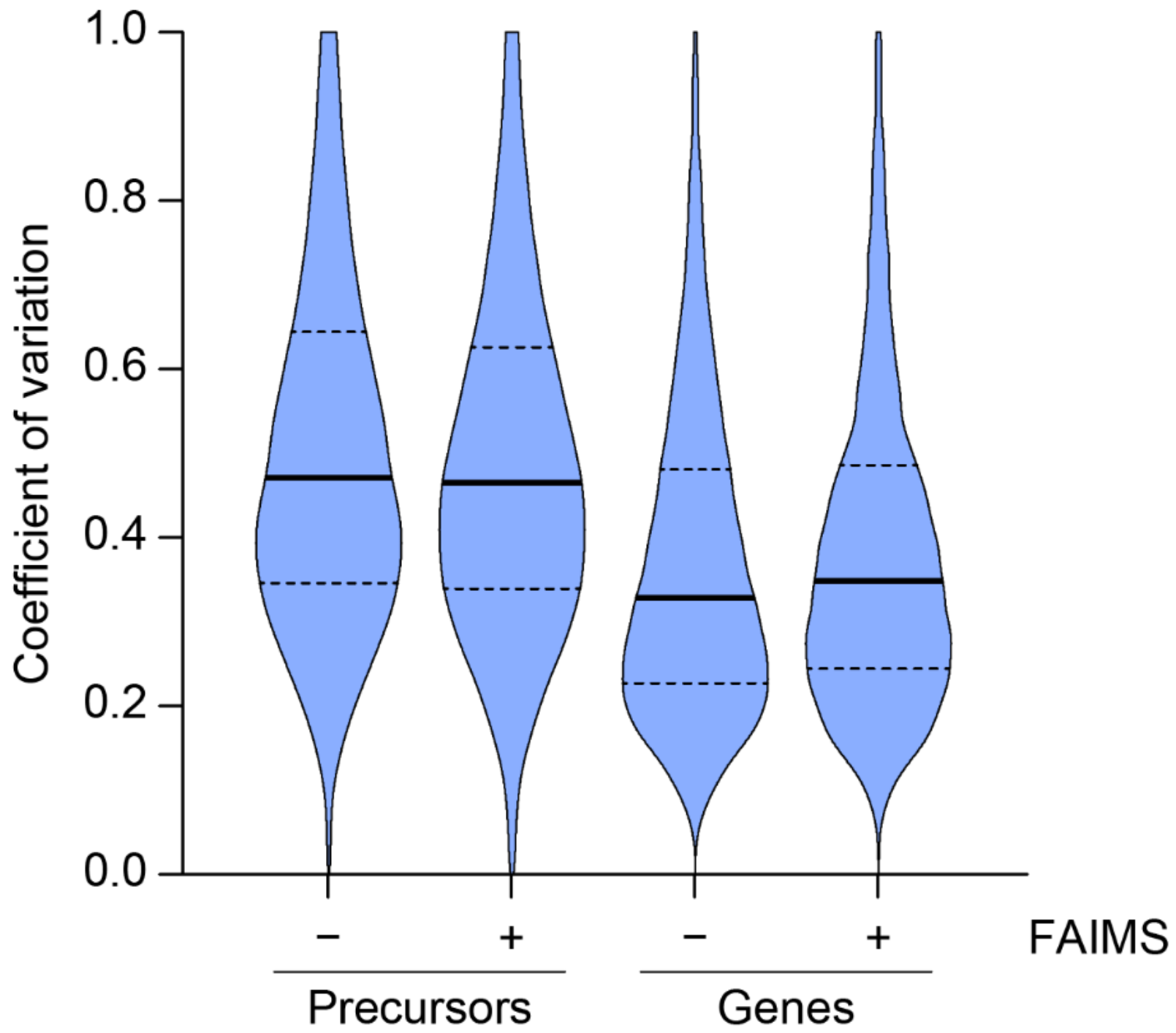

Figure R7. *Distribution of coefficient of variation for MS1-based quantification from the single HeLa cell dataset shown in Figure 4H, comparing acquisition with and without FAIMS. Quantification was performed at the precursor and protein-coding gene level using Spectronaut. Thick line; median, dashed lines; 1st and 3rd quartile. n = 12 single cells per condition.*

As also outlined above in the reply to concern #4 from this reviewer, we speculate that MS1-based quantification may benefit particularly from highly homogeneous technical dilution series, where chromatographic reproducibility and feature alignment can be optimized under highly controlled conditions, whereas single-cell datasets additionally contain true biological heterogeneity and increased sample preparation variability. As for the comparable quantitative performance between FAIMS and no-FAIMS for this single-HeLa dataset, this follows what we also observed for the HeLa dilution series (Figure 3A).

6. Single-cell and PBMC applications

For the reported 230 phosphosites in unenriched single-cell samples, please provide XICs for selected phosphopeptides and show ID score distributions relative to unmodified peptides. Are the cell sizes shown in Fig. 7b derived from the cellenONE? Given the large cell size variability of the sample, how do these measurements correlate with cell elongation and do the authors manually validate single-cell isolation?

Given the substantial differences in cell size among PBMC cell types, the observed unsupervised clustering may reflect variation in both cell type–specific markers and cell size. It would be informative to perform a similar analysis on the homogeneous HeLa dataset and overlay clustering with measured cell sizes to assess the extent to which cell size contributes to the observed separation.

Reply: We thank the reviewer for these thoughtful suggestions and have performed several additional analyses to address them.

For the phosphoproteomics analysis, we selected representative extracted ion chromatograms (XICs) for phosphopeptides, which are provided as Source Data. Furthermore, the corresponding Spectronaut (*.sne) analysis files are available through ProteomeXchange, allowing interested readers to further inspect phosphopeptide identifications and extract additional XICs directly from the analyzed datasets.

To assess the spectral quality of the phosphopeptide identifications, we compared Spectronaut Cscore and q-value distributions for phosphopeptides relative to all peptides, unmodified peptides, and several other commonly observed modifications, including NQ deamidation, KR methylation, and methionine oxidation. We observed that phosphopeptide identifications exhibited score and q-value distributions comparable to or even slightly better than unmodified peptides, whereas oxidized peptides generally exhibited lower-confidence identifications. We have incorporated these score and q-value distribution analyses in the revised manuscript (new Figure S9A-B and Figure R8).

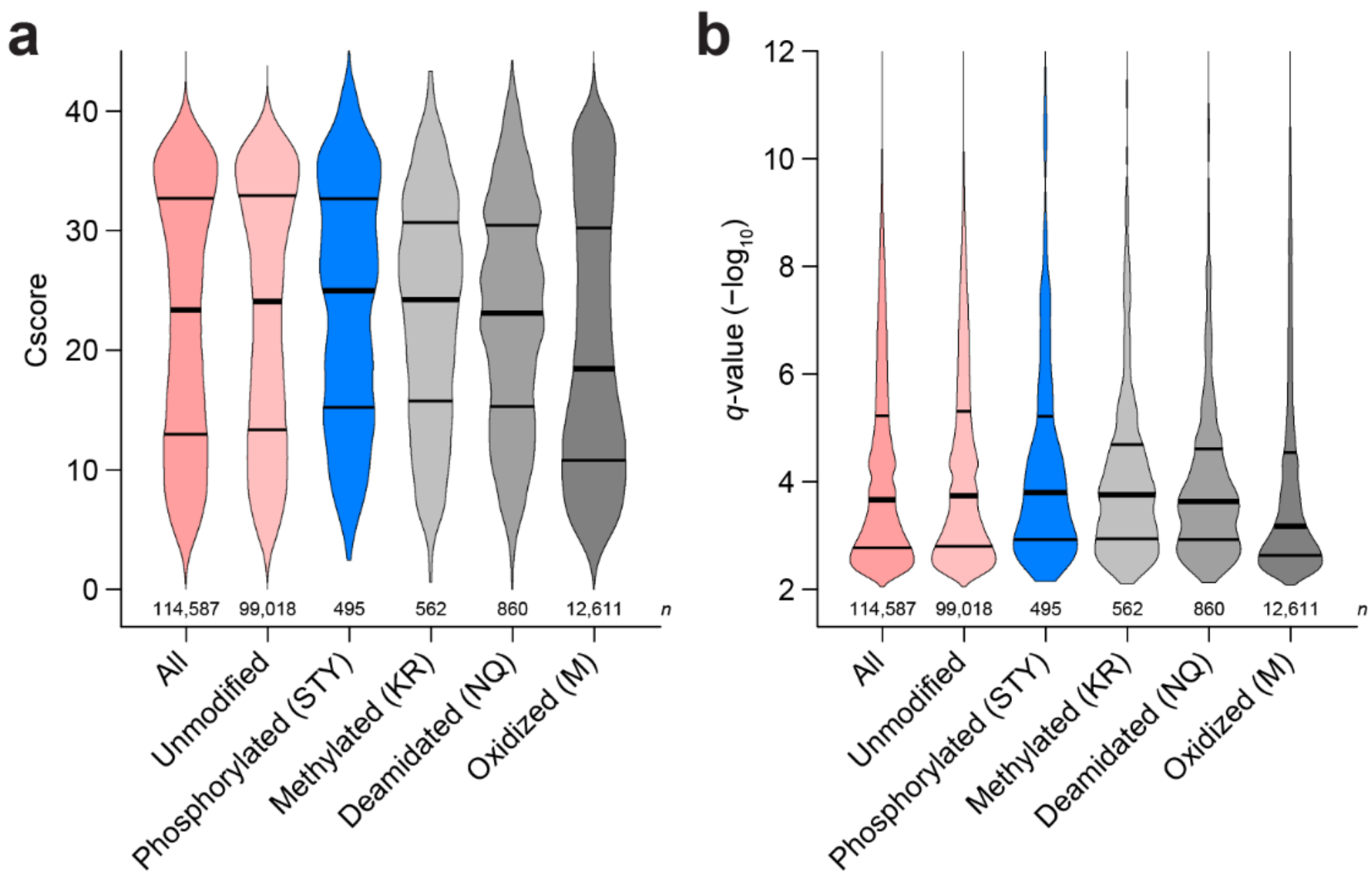

Figure R8. *(A) Violin plot distribution of Spectronaut Cscore values for the indicated classes of unmodified or modified peptides, as identified from n = 74 single HeLa cells (median diameter ~26.5 µm) analyzed at 40 SPD, see* ***Figure 5A****. Thick line; median, thin lines; 1st and 3rd quartile. Peptide n per distribution are indicated above the x-axis. (B) As* ***A****, but visualizing peptide* $-\log_{10}$ *q-value.*

These new data are described in the Results section as follows:

"*Reassuringly, phosphopeptide identifications displayed Cscore and q-value distributions comparable to, or slightly better than, unmodified peptides and other commonly observed peptide modifications (Fig. S9A-B), supporting the overall confidence of phosphosite identification despite the absence of phosphopeptide enrichment.*"

Regarding the PBMC cell size measurements shown in Figure 7B (i.e. Figure 8B in the revised manuscript), these values were derived from cellenONE image-based measurements. Single-cell isolations were manually validated during sorting. Following the reviewer's suggestion, we additionally evaluated whether the observed size variability correlated with cell elongation or morphology (Figure R9). We did not observe any notable relationship between cell size and elongation.

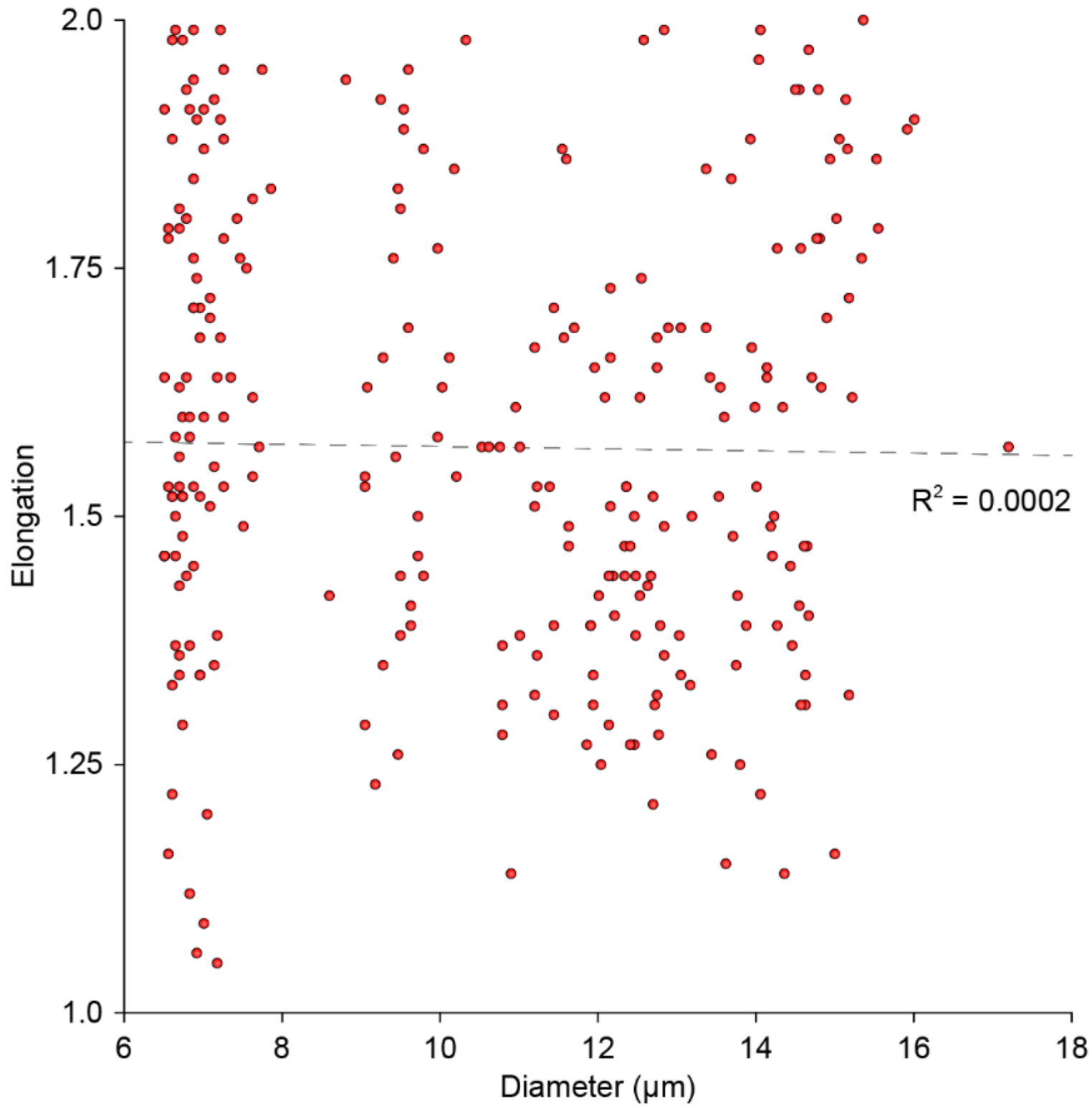

Figure R9. *Relationship between PBMC diameter and elongation as determined from cellenONE image-based measurements during single-cell isolation. Each dot represents one isolated PBMC. Linear regression analysis did not reveal a notable correlation between cell size and elongation ($R^2 = 0.0002$).*

Finally, to directly assess whether cell size alone could explain the observed clustering behavior, we performed an analogous UMAP analysis using the homogeneous single-HeLa-cell dataset from Figure 5 and overlaid the measured cell size quartiles (Figure R10). In contrast to the PBMC data, we did not observe clustering associated with cell size in the HeLa dataset. This suggests that the separation observed in the PBMC analyses is not solely driven by cell size differences, but more likely reflects underlying biological heterogeneity between PBMC subpopulations together with differences in cellular protein content.

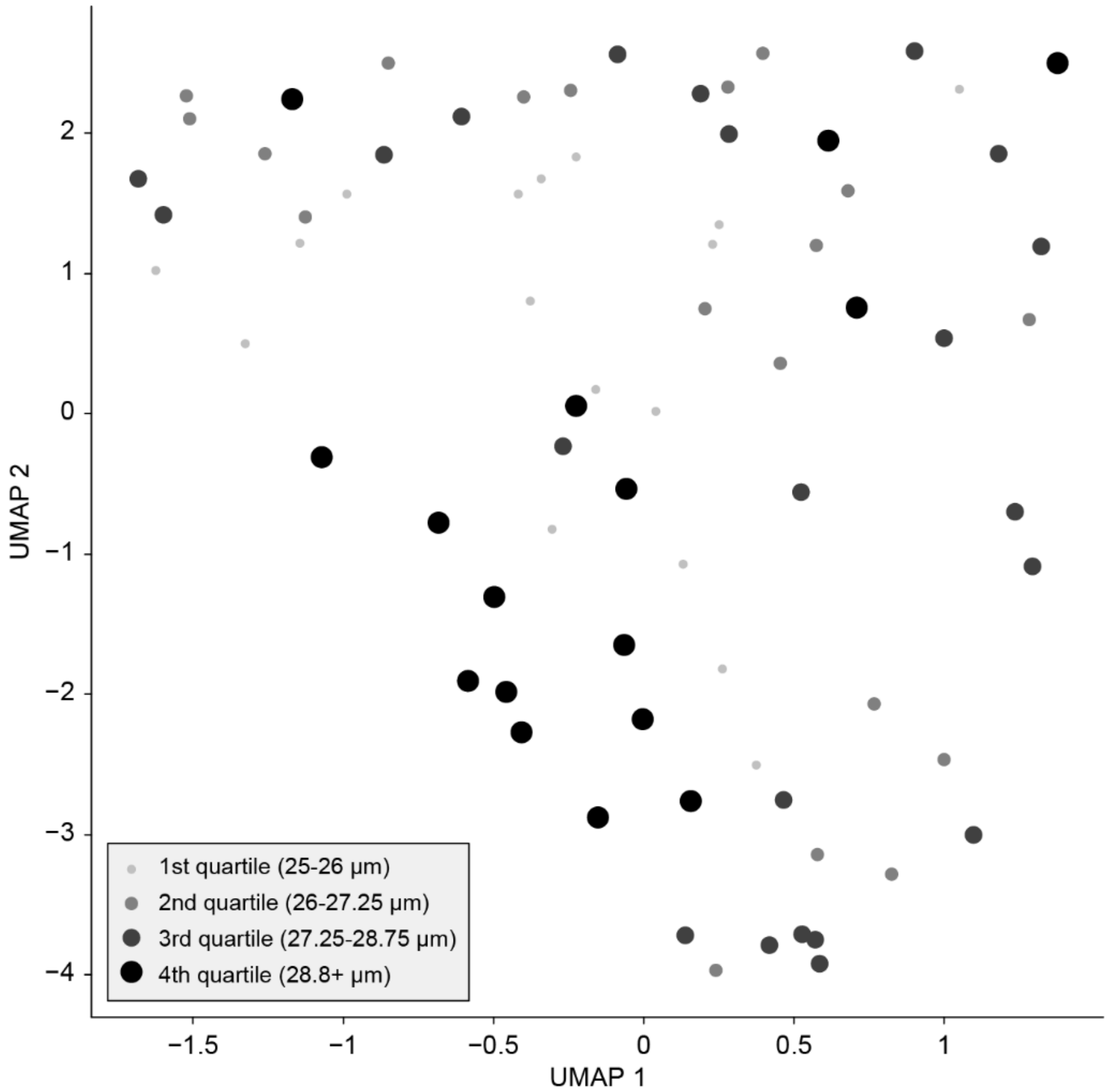

Figure R10. *UMAP analysis of the homogeneous single-HeLa-cell dataset from Figure 5, overlaid with measured cell size quartiles as determined during cellenONE isolation. Dot size and shading correspond to cell diameter quartiles. In contrast to the PBMC dataset, no clear clustering associated with cell size was observed.*

Minor points

In Fig. 1c, the 250 pg condition without DDM yields nearly 10,000 more precursors than the corresponding 250 pg condition in Fig. 1b. Please clarify the source of this discrepancy.

Reply: We apologize for the confusion. We have now annotated Figure 1, Figure S1, and Supplementary Note 1, with additional experimental details in the same style as all the other figures.

To directly answer the question; the experiments in Figure 1B were performed using a 15-minute analytical gradient, column delay of 6 min, ~7 min active gradient, at 72 SPD. The experiments in Figure 1C were performed using a 20-minute analytical gradient, column delay of 6 min, ~12 min active gradient, at 54 SPD. Thus, the increased number of identifications is almost certainly due to the longer active gradient.

In comparison, the gradient we present in Figure 1G (which is used throughout the vast majority of the manuscript) is a 15-minute analytical gradient, column delay of 2 min, ~12 min active gradient, at 72 SPD.

To summarize;

Figure	Gradient length	Column delay	Active elution	Samples-per-day
1B	15 min	6 min	7 min	72
1C	20 min	6 min	12 min	54
1G (and beyond)	15 min	2 min	12 min	72

We would also like to point out that Supplementary Data 1 contains a comprehensive overview of all LC-MS settings across all raw data files, mapped to all experiments and figure panels. To make this more apparent to readers interested in the technical implementation, we now explicitly reference Supplementary Data 1 in the first paragraph of the results section:

"A comprehensive overview of all LC-MS acquisition parameters for all experiments in this work is provided in Supplementary Data 1."

Throughout the manuscript the authors nicely pair IDs with CV plots, please also amend the CVs for Fig. 1b–c, f and g.

Reply: Following the reviewer's suggestion, we have now included CV violin plots for Figures 1B, C, F, and G. These new plots are available as Supplementary Figure 1B-C, E-F, H-I, and K-L, respectively. Notably, these CVs were derived from data processed without MBR.

Please explicitly state the flow rate corresponding to the term "high-flow."

Reply: The "high flow" or "higher flow" we refer to is 500 nL/min. We have changed all instances of "high flow" to "higher flow", and now specifically mention the flow rate, e.g. in the Results section:

"*higher flow (i.e. 500 nL/min)*"

In lines 486 onward, the authors suggest that the benefit of DDM primarily arises from improved peptide recovery rather than cell lysis. However, the detergent serves a dual function in cell lysis and peptide recovery, as no alternative lysis reagent was evaluated this statement needs to be adapted.

Reply: We fully agree with the reviewer, and we cannot formally decouple cell lysis and peptide recovery in this case. Thus, we have rephrased the statement as follows:

"*Although concentrations of 0.1% DDM are typically used in master mix, we did not observe a difference in protein recovery efficiency when decreasing the concentration to 0.01% DDM. This observation suggests that lower concentrations of DDM may already be sufficient to support efficient sample processing under our experimental conditions.*"

Please clarify which acquisition method was used for the FAIMS CV evaluation (Fig. 2d) and the PBMC data (Fig. 7-8).

Reply: We apologize for not including these details in the figure legends in the initial submission. For Figure 2D and the associated Figure S4, a 10 Th and 20 ms acquisition strategy was used, scanning across a range of 400-800 m/z. These details have been added to the figure (Th & ms) and legend (scan range) in the respective figures. PBMC data was acquired using 400-800 m/z, 20 Th, 40 ms; these details have been added to the Figure 7C-D and S11C-D legends.

Please clarify if the reported CV in Fig. 3h is derived from data processed with or without MBR?

Reply: CVs in Figure 3H were derived from data processed without MBR, using DIA-NN v1.9.2. This has been clarified in the figure legend.

Please clarify whether the no-FAIMS methods involved physical removal of the FAIMS unit or disabling FAIMS filtering.

Reply: No-FAIMS methods involve physical absence of the FAIMS unit.

We have clarified this in the Methods section:

"*We note that "no-FAIMS" always refers to physical absence of the FAIMS unit, rather than just disabling the device.*"

In line 415, the authors refer to “minimally processed” cells. Please clarify which processing steps are included or omitted under this definition.

Reply: Minimally processed is following the Lymphoprep protocol, as outlined in the Methods section, where a considerable pellet was retained after centrifugation. The “small” population was derived from several more steps of cell re-suspension and centrifugation to keep only the smallest cells.

We have now clarified at the end of the “Peripheral blood mononuclear cells preparation” Methods section, as follows:

“*We note that the above procedure results in PBMCs we refer to as “minimally processed” or “Mixed” population. Alternatively, PBMCs were washed several times with PBS and centrifuged at 500g, removing the larger cells that had pelleted, and keeping the supernatant containing only the smallest PBMCs; referred to as the “Small” population.*”

REFERENCES

Bubis, J.A., Arrey, T.N., Damoc, E., Delanghe, B., Slovakova, J., Sommer, T.M., Kagawa, H., Pichler, P., Rivron, N., Mechtler, K.*, et al.* (2025). Challenging the Astral mass analyzer to quantify up to 5,300 proteins per single cell at unseen accuracy to uncover cellular heterogeneity. Nat Methods *22*, 510-519.

Ctortecka, C., Clark, N.M., Boyle, B.W., Seth, A., Mani, D.R., Udeshi, N.D., and Carr, S.A. (2024). Automated single-cell proteomics providing sufficient proteome depth to study complex biology beyond cell type classifications. Nat Commun *15*, 5707.

Furtwangler, B., Uresin, N., Richter, S., Schuster, M.B., Barmpouri, D., Holze, H., Wenzel, A., Gronbaek, K., Theilgaard-Monch, K., Theis, F.J.*, et al.* (2025). Mapping early human blood cell differentiation using single-cell proteomics and transcriptomics. Science *390*, eadr8785.

Guzman, U.H., Martinez-Val, A., Ye, Z., Damoc, E., Arrey, T.N., Pashkova, A., Renuse, S., Denisov, E., Petzoldt, J., Peterson, A.C.*, et al.* (2024). Ultra-fast label-free quantification and comprehensive proteome coverage with narrow-window data-independent acquisition. Nat Biotechnol *42*, 1855-1866.

Guzman, U.H., Rykaer, M., Hendriks, I.A., Stewart, H., Denisov, E., Hagedorn, B., Petzoldt, J., Kreutzmann, A., Mueller, Y., Arrey, T.N.*, et al.* (2026). Higher-Throughput Proteome Profiling Enabled by Parallelized Pre-Accumulation and Optimized Ion Processing in the Orbitrap Astral Zoom Mass Spectrometer. Mol Cell Proteomics *25*, 101504.

Hsu, C., Shulman, N., Stewart, H.I., Petzoldt, J., Pashkova, A., Plubell, D.L., Denisov, E., Hagedorn, B., Damoc, N.E., MacLean, B.X.*, et al.* (2025). Evaluation of a Prototype Orbitrap Astral Zoom Mass Spectrometer for Quantitative Proteomics horizontal line Beyond Identification Lists. J Proteome Res *24*, 5742-5754.

Huang, S., Truong, T., Wang, C., Xie, X., Lin, H.L., and Kelly, R.T. (2026). Optimization of trap column properties and loading conditions for proteome profiling of single-cell-level sample inputs. Anal Bioanal Chem.

Petrosius, V., Aragon-Fernandez, P., Arrey, T.N., Woessmann, J., Uresin, N., de Boer, B., Su, J., Furtwangler, B., Stewart, H., Denisov, E.*, et al.* (2025). Quantitative Label-Free Single-Cell Proteomics on the Orbitrap Astral MS. Mol Cell Proteomics *24*, 100982.

Specht, H., Emmott, E., Petelski, A.A., Huffman, R.G., Perlman, D.H., Serra, M., Kharchenko, P., Koller, A., and Slavov, N. (2021). Single-cell proteomic and transcriptomic analysis of macrophage heterogeneity using SCoPE2. Genome Biol *22*, 50.

Ye, Z., Sabatier, P., van der Hoeven, L., Lechner, M.Y., Phlairaharn, T., Guzman, U.H., Liu, Z., Huang, H., Huang, M., Li, X.*, et al.* (2025). Enhanced sensitivity and scalability with a Chip-Tip workflow enables deep single-cell proteomics. Nat Methods *22*, 499-509.

Zheng, R., Matzinger, M., Mayer, R.L., Valenta, A., Sun, X., and Mechtler, K. (2023). A High-Sensitivity Low-Nanoflow LC-MS Configuration for High-Throughput Sample-Limited Proteomics. Anal Chem *95*, 18673-18678.

Nature Communications manuscript
By Hendriks, Buch-Larsen et al.

Point-by-point responses to the reviewers' comments

We sincerely thank the Reviewers for their careful evaluation of our revised manuscript and for their constructive comments. We are pleased that the reviewers found the major concerns raised during the previous review to have been satisfactorily addressed, and we appreciate the opportunity to clarify the remaining points.

In response to the comments received during this revision, we performed several additional experiments, further clarified key methodological aspects, and expanded the manuscript where appropriate. To preserve the overall flow of the manuscript, all new experimental data have been incorporated as Supplementary Notes.

Summary of revisions

- Performed a direct comparison of the 300–1000 Th, 400–800 Th, and optimized 425–625 Th acquisition strategies, further supporting the advantages of the narrow-range workflow under low-input conditions.
- Added a direct comparison between the original 385–585 Th and refined 425–625 Th acquisition windows, demonstrating a modest but consistent improvement with the optimized method.
- Validated the workflow using a commercially available analytical column with specifications matching our in-house columns, demonstrating essentially identical chromatographic and proteomic performance.
- Added representative TIC chromatograms illustrating DDM accumulation at excessive concentrations and confirming that the optimized DDM concentrations used throughout this study do not produce detectable chromatographic carryover.
- Included a new Table 1 summarizing our recommended LC-MS acquisition strategies for different sample input levels and analytical objectives.

We believe these revisions substantially strengthen the manuscript and address the reviewers' remaining concerns. Our point-by-point responses are presented in **blue text** throughout this document.

Reviewer #1 (Remarks to the Author):

I carefully reviewed the revised version of the manuscript and the authors' response to the reviewers. Overall, the authors have addressed all of my major comments. However, a few concerns arose from the revised version.

Reply: We thank the reviewer for the careful evaluation of our revised manuscript. We are pleased that the reviewer finds that the major concerns raised during the previous review have been satisfactorily addressed, and we appreciate the opportunity to clarify the remaining points below.

Primarily, the main claim of the manuscript is the establishment of a narrow mass range of 385–585 Th. However, several results presented in the revised version raise doubts regarding the importance and general applicability of this analytical approach. First, throughout the manuscript, the authors refer to the mass range of 300–1000 Th as the standard. However, in many proteomics studies, the commonly used range is considerably narrower, typically around 400–800 Th. Since the authors did not perform a direct comparison to this commonly used mass range, it is difficult to fully assess the benefit of employing such a narrow window. In fact, the authors state that they used the 400–800 Th range in Figures 5–8, suggesting that the narrower range may not provide a substantial advantage in practice.

Reply: We thank the reviewer for this helpful suggestion. We agree that directly comparing our optimized narrow-range method to a more commonly used intermediate scan range strengthens the manuscript. While broader scan ranges such as 300–1000 Th (or 380–980 Th) are frequently used in conventional DIA workflows (Guzman et al., 2024; Raja et al., 2025), narrower ranges around 400–800 Th are also commonly employed, particularly in conjunction with FAIMS-assisted low-input proteomics (Bubis et al., 2025). Notably, the 400–800 Th mass ranges used in **Figures 5-8** were all in the context of FAIMS analysis.

To more directly address this concern, we have now performed a new experiment where we directly compare the 300–1000 Th, 400–800 Th, and 425–625 Th methods, using 25 pg of HeLa lysate, representing ultra-low-input amounts comparable to that encountered for small PBMCs (**Figure R1** and **Supplementary Note 8**). In all cases, 4 Th isolation windows were used, and injection times were balanced to keep cycle time the same.

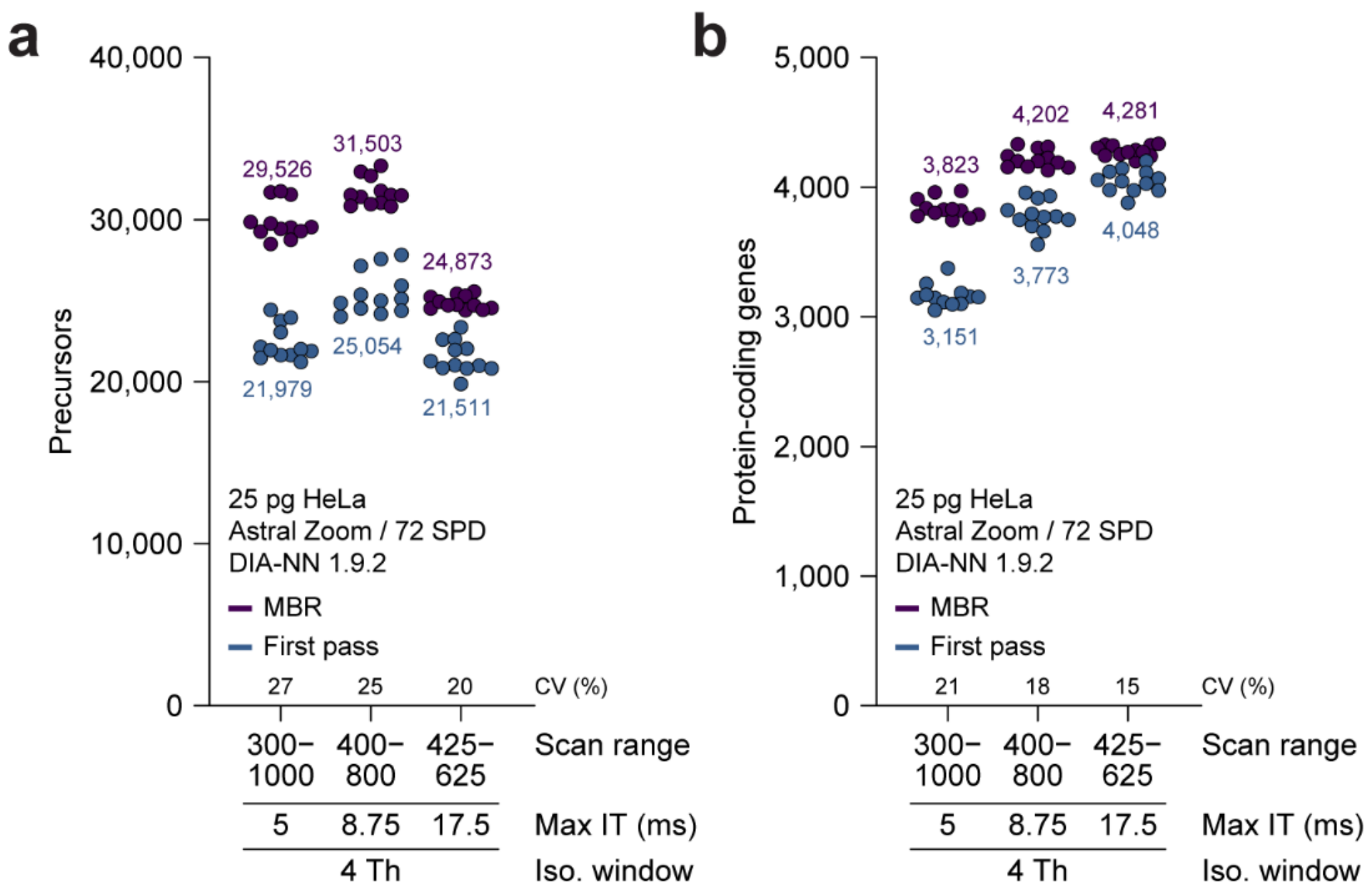

Figure R1 / Supplementary Note 8. Comparison of 300-1000, 400-800, and 425-625 methods. *(A) Number of precursors identified from 25 pg HeLa lysate. All analyses performed using the Orbitrap Astral Zoom MS, and data analyzed using DIA-NN in either first pass or matching-between-runs (MBR) mode. Exact median values are displayed above each group of data for MBR, and below each group of data for first pass. Numbers above the x-axis represent median coefficient of variation percentage, n = 12 technical replicates. "Max IT"; maximum injection time, "Iso. window"; isolation window. (B) As* ***A****, but for protein-coding genes.*

Based on this experiment, and when considering first-pass (no MBR) data, we found that the 425–625 Th method identified a comparable number of precursors relative to the 300–1000 Th method, yet considerably more protein-coding genes. These findings are consistent with the central concept underlying our ultra-sensitive nDIA approach: progressively restricting the acquisition range reduces precursor complexity while increasing ion accumulation time per isolation window. Although the broader 400–800 Th method yielded more peptide identifications overall, the more focused 425–625 Th window further enriched for the most information-dense precursor population, resulting in a higher number of protein identifications together with improved quantitative precision. Importantly, this trend is fully consistent with the observations already reported in the manuscript (**Figure 1F**, **Figure 2B**, **Figure 4D**, and **Supplementary Note 7**).

Together, these additional experiments further support our conclusion that, in the context of low-input proteomics without FAIMS, the 425–625 Th acquisition strategy provides the most favorable balance between proteome depth and quantitative precision compared to the wider 400–800 Th and 300–1000 Th acquisition ranges evaluated here.

These new data are included in **Supplementary Note 8** and described in the Results section with the following text:

"*In addition, direct comparison with broader 300 – 1000 m/z and intermediate 400 – 800 m/z acquisition strategies confirmed that the 425 – 625 m/z method provides the best overall balance between protein identification depth and quantitative precision under low-input conditions (Supplementary Note 8).*"

Finally, in response to Reviewer 2 (see below), and to further assist readers in selecting an appropriate acquisition strategy for different experimental settings, we have also included the new **Table 1** into the manuscript that lists general recommendations for MS methodology depending on sample load.

Second, it appears that the most influential parameter in the entire study is the column inner diameter (i.d.), yet this parameter is only presented in the first figure. One limitation is that the column used is home-made, which may limit the applicability of the results to other mass spectrometry laboratories that primarily rely on commercial columns.

Reply: We thank the reviewer for raising this important point. We agree that the use of a 50 µm internal diameter analytical column is one of the key components contributing to the performance of the workflow presented here. In our implementation, the smaller inner diameter allows short peptide elution delay to be maintained while operating at comparatively low analytical flow rates (125 nL/min), thereby preserving chromatographic performance and ionization efficiency. Together with the optimized MS acquisition strategy and low-input sample preparation, this forms an important part of the overall workflow.

We fully agree that in-house production and packing of laser-pulled silica columns is not something that is readily available to all laboratories. To address this, we therefore acquired a commercially available analytical column from Dr. Maisch with identical specifications to our in-house packed columns and directly compared their performance using a single cell equivalent load of 250 pg HeLa lysate (**Figure R2** and **Supplementary Note 1**). Both columns were 20 cm in length, 50 µm internal diameter, incorporated an integrated pulled emitter, and were packed with ReproSil-Pur 120 C18-AQ (1.9 µm) particles.

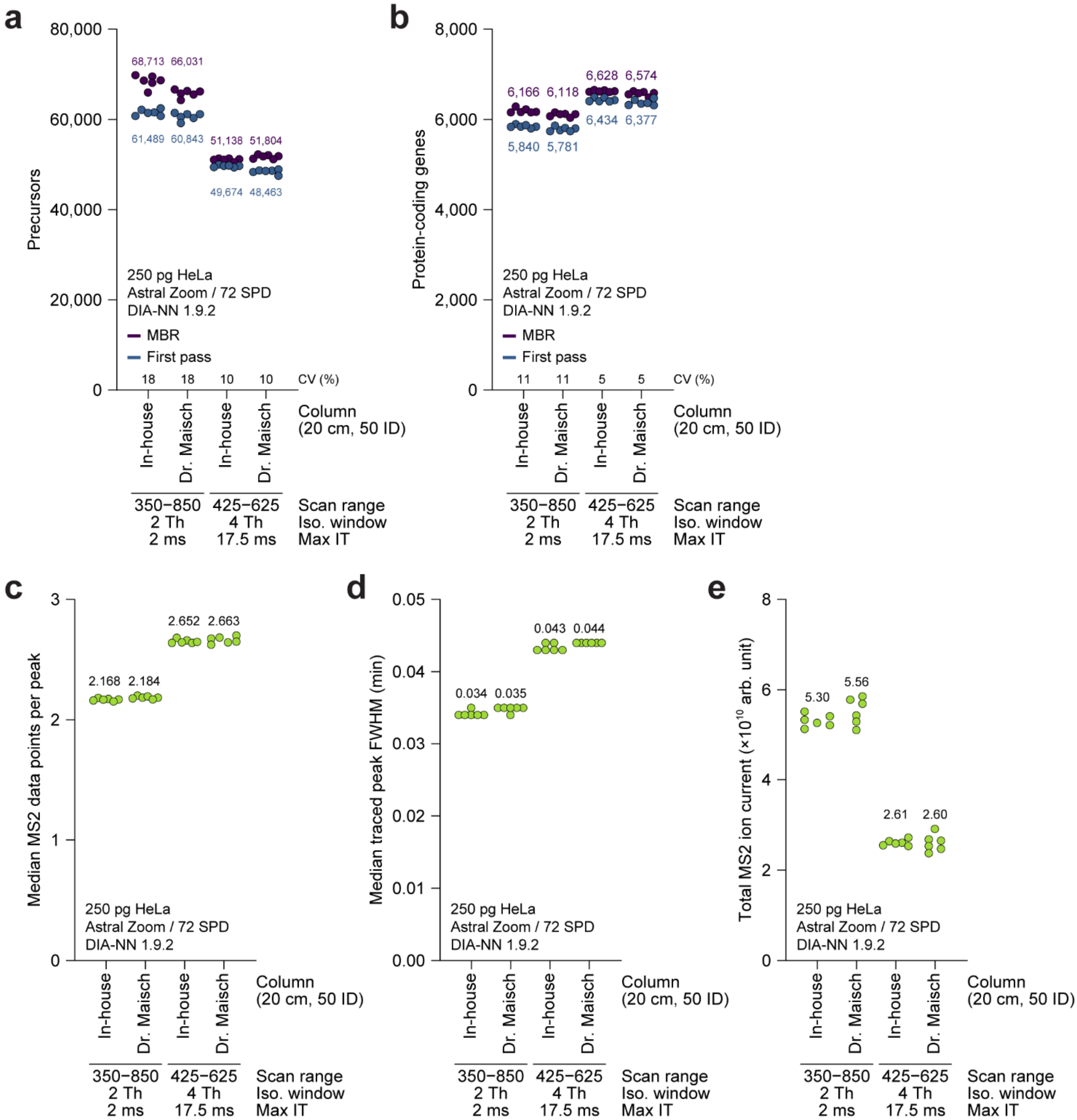

Figure R2 / Supplementary Note 1. Comparison of an in-house produced column with a commercial column from Dr. Maisch*. (A) Number of precursors identified from 250 pg HeLa lysate. All analyses performed using the Orbitrap Astral Zoom MS, and data analyzed using DIA-NN in either first pass or matching-between-runs (MBR) mode. Exact median values are displayed above each group of data for MBR, and below each group of data for first pass. Numbers above the x-axis represent median coefficient of variation percentage, n = 6 technical replicates. "Max IT"; maximum injection time, "Iso. window"; isolation window. (B) As* ***A****, but for protein-coding genes. (C) As* ***A****, but for median number of MS2 data points per quantified peak. (D) As* ***A****, but for median traced peak Full Width Half Maximum (FWHM). (E) As* ***A****, but for the total amount of quantified MS2 ion current per run.*

Based on this analysis of 250 pg HeLa lysate using both a broader fast-scanning method (350–850 Th, 2 Th, 2 ms) and our optimized high-sensitivity narrow-range method (425–625 Th, 4 Th, 17.5 ms), the commercial and in-house columns exhibited highly comparable performance across all evaluated metrics. In first-pass analysis (without MBR), the numbers of identified precursors and protein-coding genes differed by approximately 1%. Likewise, quantitative precision, the number of MS2 data points per peak, chromatographic peak width (FWHM), and quantified MS2 ion current were essentially indistinguishable between the two column formats.

These results indicate that the performance gains described in this study are not dependent on in-house column fabrication. Rather, comparable chromatographic performance can be achieved using commercially available analytical columns with matching specifications. Notably, the commercial column was evaluated using both a conventional broad-range DIA method and our optimized ultra-sensitive nDIA method, indicating that the observed agreement was not specific to a single acquisition strategy.

We have incorporated these data into the revised manuscript as the new **Supplementary Note 1**, together with the following text:
"*Importantly, we observed essentially identical chromatographic and proteomic performance when comparing our in-house packed 50 µm ID column with a commercially available analytical column of identical dimensions and stationary phase, demonstrating that this workflow can readily be implemented without in-house column fabrication (Supplementary Note 1).*"

A further point of confusion is that the main text focuses on a mass range of 385–585 Th, whereas the supplementary material shifts to a range of 425–625 Th. I could not find an explanation for this change, and clarification would be helpful.

Reply: We appreciate the reviewer pointing out this lack of clarity. The two acquisition windows represent successive stages of the methodological optimization, rather than different analytical strategies. The initial 385–585 Th window was selected based on the in silico analysis presented in **Figure 1D-F** and was therefore used throughout the early phases of method development. Subsequently, experimental gas-phase fractionation (**Figure 2C**) identified a modest shift in the optimum precursor distribution, leading us to refine the acquisition window to 425–625 Th for later stages of method development.

To further clarify that this refinement reflects an experimentally supported refinement rather than an arbitrary change in acquisition parameters, we additionally performed a direct comparison between the 385–585 Th and 425–625 Th methods using 250 pg HeLa lysate (**Figure R3** and **Supplementary Note 6**).

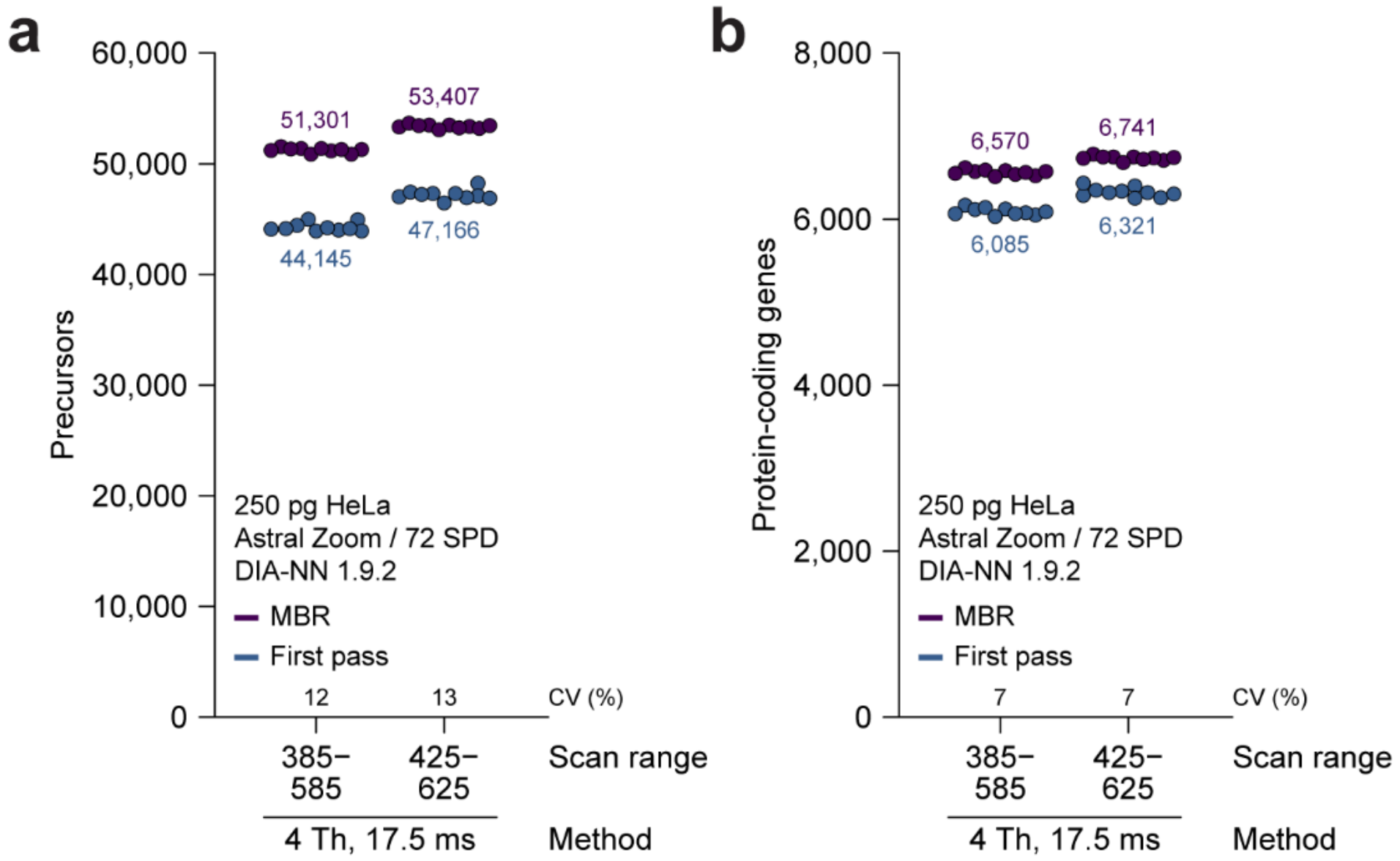

Figure R3 / Supplementary Note 6. Comparison of 385-585 and 425-625 methods*. (A) Number of precursors identified from 250 pg HeLa lysate. All analyses performed using the Orbitrap Astral Zoom MS, and data analyzed using DIA-NN in either first pass or matching-between-runs (MBR) mode. Exact median values are displayed above each group of data for MBR, and below each group of data for first pass. Numbers above the x-axis represent median coefficient of variation percentage, n = 10 technical replicates. (B) As* ***A****, but for protein-coding genes.*

These additional data demonstrate that the experimentally refined 425–625 Th window consistently outperformed the original 385–585 Th window, increasing precursor identifications by approximately 7% and protein identifications by approximately 4% while maintaining comparable quantitative precision. Importantly, both acquisition windows support the principal conclusions of the manuscript; however, the experimentally refined 425–625 Th window provided a consistent, albeit modest, improvement in identification performance and was therefore selected as the recommended acquisition window for low-input analyses.

We have included this data in the manuscript as the new **Supplementary Note 6**, and included the following sentence in the manuscript to highlight the rationale for switching to 425-625 Th:

"*Based on these experimental results, we refined our narrow acquisition window from 385–585 Th to 425–625 Th for subsequent method development; direct comparison between the two acquisition windows*

confirmed a modest but consistent improvement in identification performance with the refined method (Supplementary Note 6)."

Finally, we have also included the 425-625 method as our recommendation for low-input analysis in **Table 1**.

Overall, I still value the contribution of the publication of the optimized details of all running parameters, however, given that the narrow mass range doesn't seem to provide a major benefit compared to a commonly used method, I doubt that the advancement of this study is sufficient for publication in Nature Communications.

Reply: We thank the reviewer for sharing this perspective. We respectfully believe that the contribution of the present work extends beyond optimization of a single acquisition parameter. Rather, the manuscript presents a systematic optimization and integration of low-input sample preparation, chromatography, DIA acquisition strategy, Orbitrap Astral Zoom operation, and FAIMS-assisted analysis into a unified label-free single-cell proteomics workflow. Collectively, these developments enabled robust profiling of more than 7,000 proteins from individual HeLa cells, deep proteome coverage from very small primary PBMCs without carrier channels or labeling strategies, and analytical throughput exceeding 100 samples per day while maintaining substantial proteome depth.

Throughout the revision process, we have carefully moderated our claims to better distinguish methodological optimization from conceptual novelty, expanded discussion of related work, and incorporated the additional experiments requested during peer review, including comparisons with intermediate scan ranges, commercial analytical columns, and refined acquisition windows. We hope these revisions more clearly convey both the practical value and the broader applicability of the optimized workflow presented here.

Reviewer #2 (Remarks to the Author):

In the revised version of this manuscript, the authors clearly lay out the impact of their chromatographic methods and include a trap-and-elute workflow that pushes the throughput of most present SCP work. I want to particularly highlight the improved tone of the overall manuscript in relation to previous work.

Reply: We are grateful to the reviewer for the thoughtful re-evaluation of our manuscript and for the positive assessment of the revisions. We particularly appreciate the acknowledgement that the revised manuscript more clearly positions our work within the context of previous studies.

In this context, I want to highlight that their statement in the abstract 'routinely quantified over 7,000 proteins per individual HeLa cell at over 100 samples-per-day.', as most of their work yields ~6800 proteins, while impressive, this statement should still be adapted to e.g., "approaching 7,000 proteins at over 100 SPD".

Reply: We thank the reviewer for this helpful suggestion. We have revised the statement in the abstract from "over 7,000 proteins per individual HeLa cell" to "up to 7,000 proteins per individual HeLa cell", which more accurately reflects the performance observed across the different throughput conditions.

Additionally, I appreciate the clarification regarding the DDM amounts and the details regarding the reduction without alkylation used across experiments in the revised manuscript. However, the manuscript could explicitly state the risk of DDM carryover and impacted chromatographic separation indicated in Figure R3. While the impact is to be expected, many users assume any non-ionic detergent can be added without consequences. The authors' TIC data clearly illustrate the progressive accumulation across consecutive injections, which is not sufficiently described in the methods or discussion. Please add a concise statement that DDM concentrations above 500 ng (2 µg) per injection were found to impact chromatographic separation under standard operation and that the ~100 ng were found sufficient for the indicated workflow.

Reply: We thank the reviewer for this valuable suggestion. We agree that explicitly discussing the practical consequences of excessive DDM usage will improve the manuscript, particularly for readers seeking to implement similar low-input workflows.

To directly address this point, we have incorporated representative TIC chromatograms (from the experiments shown in Figure 1B) into **Figure R4** to illustrate the progressive accumulation of late-eluting hydrophobic signal observed when injecting 2 µg DDM (**Figure R4A**). We additionally included representative TIC chromatograms (from the Figure 1C experiments), demonstrating that under the optimized workflow (~100 ng DDM per injection; 500 ng in the experiment shown), no comparable accumulation is observed across consecutive injections (**Figure R4B**).

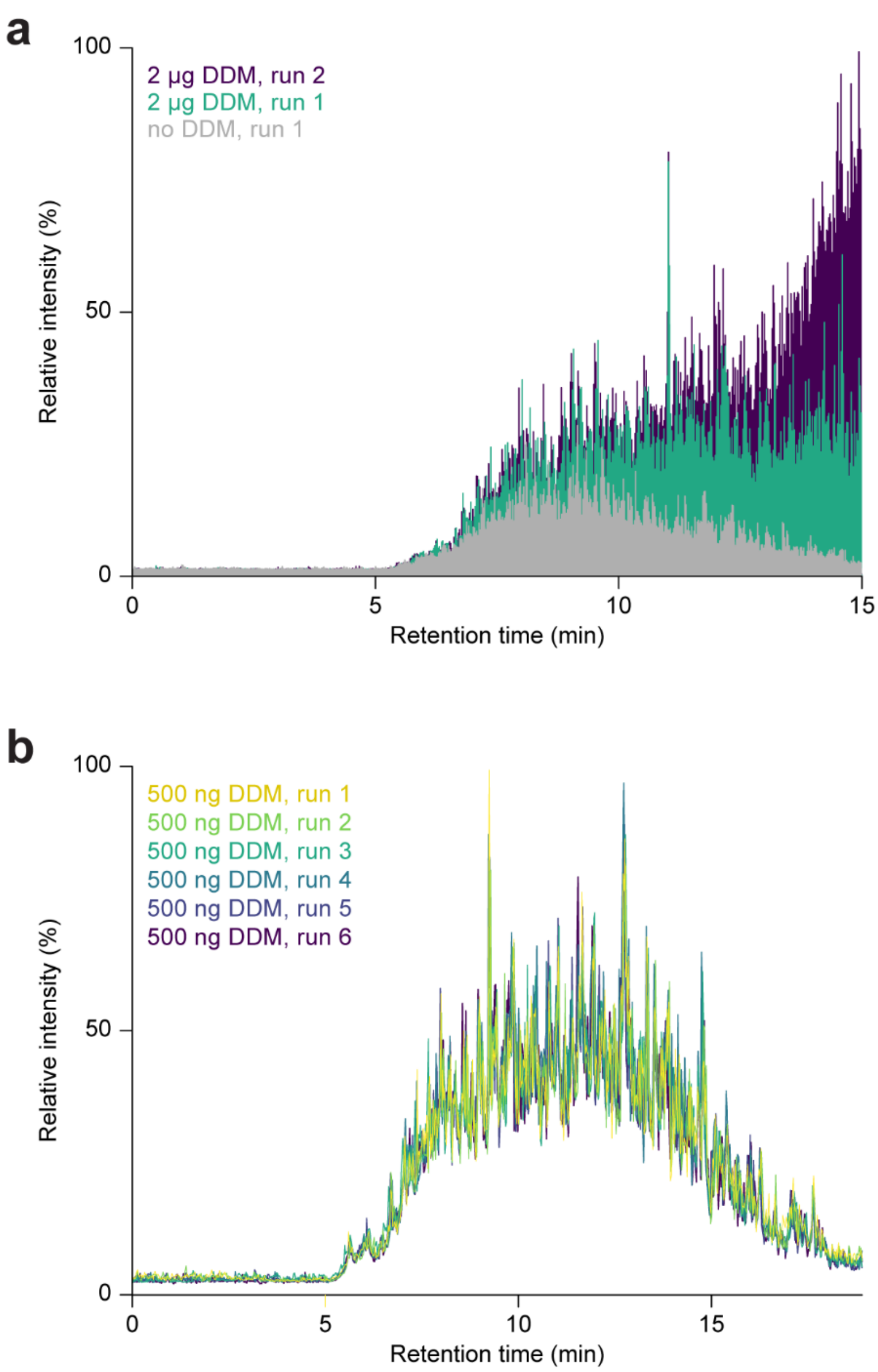

Figure R4 / Supplementary Note 2. Risk of DDM accumulation when using excessive amounts. *(A) Representative total ion current (TIC) chromatograms (data from figure 1B) illustrating accumulation of hydrophobic signal following repeated injections of high amounts of DDM along with 250 pg of HeLa lysate. Shown are consecutive injections*

containing 2 µg DDM ("DDM, run 1" and "DDM, run 2") compared to a control injection without DDM. (B) Representative TIC chromatograms (data from figure 1C) demonstrating no build-up of hydrophobic signal when injecting 500 ng DDM along with 250 pg of HeLa lysate, indicating that DDM is sufficiently cleared from the analytical column between runs.

We have included this data in the manuscript as the new **Supplementary Note 2**, and included the following sentence in the manuscript to clarify the practical concentration range of DDM that can be used without introducing detectable chromatographic carryover under our experimental conditions:
"*Notably, substantially higher DDM amounts resulted in gradual accumulation of hydrophobic material on the LC system, whereas the optimized concentrations used throughout this study did not (Supplementary Note 2).*"

Lastly, I find the author's discussion in response to reviewer 1 comment 4 and reviewer 2 comment 5 very interesting and agree that most combinations of acquisition parameters should be tailored for specific use cases. While Supplementary Data 1 gives a great overview of all methods used in this study, most readers would benefit from a comprehensive overview of their methods tested with suggestions by the authors, based on their experience, for which input amount, experimental considerations, or sample preparation these parameters would be optimal (or not recommended).

Reply: We thank the reviewer for this excellent suggestion and agree that a practical summary of the optimized workflows will improve the accessibility of the manuscript. To address this, we have added Table 1 to the main manuscript (and also below), which summarizes the recommended acquisition strategies based on sample input, analytical priorities, and our experience throughout this study. The table highlights the situations in which we recommend conventional DIA, sensitive narrow-range DIA, or ultra-sensitive FAIMS-assisted acquisition, together with practical considerations regarding chromatography configuration and sample throughput.

Intended application	Approximate input range	Single cells	Recommended workflow	Primary objective	Notes
Conventional DIA proteomics	>1 ng	–	Broad scan range (e.g. 300–1000 or 380–980 Th), 2–4 Th, 2.5–5 ms	Maximum peptide coverage	Highest peptide coverage; preferred for peptide-centric applications (e.g. PTM analysis) when sample amount is not limiting.
Low-input DIA proteomics	50 pg – 1 ng	Larger cells (>15 µm), e.g. HeLa	425–625 Th, 4 Th, 17.5 ms	Maximum protein depth	Best balance between proteome depth and quantitative precision for low-input samples.
Ultra-low-input DIA proteomics	<50 pg	Smaller cells (<15 µm), e.g. PBMCs	400–800 Th + FAIMS, 20 Th, 40 ms	Maximum analytical sensitivity	Highest sensitivity below ~50 pg by suppressing singly charged background. Reduced peptide coverage could make this workflow less suitable for peptide-centric applications.
Recommendations are based on the workflows evaluated in this study and are intended as general guidance. Optimal acquisition parameters may vary depending on LC configuration, chromatographic performance, sample complexity, and the mass spectrometer used.					

Table 1. *Recommended LC-MS acquisition strategies for different sample input levels.*

REFERENCES

Bubis, J.A., Arrey, T.N., Damoc, E., Delanghe, B., Slovakova, J., Sommer, T.M., Kagawa, H., Pichler, P., Rivron, N., Mechtler, K*., et al.* (2025). Challenging the Astral mass analyzer to quantify up to 5,300 proteins per single cell at unseen accuracy to uncover cellular heterogeneity. Nat Methods *22*, 510-519.

Guzman, U.H., Martinez-Val, A., Ye, Z., Damoc, E., Arrey, T.N., Pashkova, A., Renuse, S., Denisov, E., Petzoldt, J., Peterson, A.C*., et al.* (2024). Ultra-fast label-free quantification and comprehensive proteome coverage with narrow-window data-independent acquisition. Nat Biotechnol *42*, 1855-1866.

Raja, R., Biswas, B., Abraham, R., Wang, Y., Chang, C.Y., Hendriks, I.A., Buch-Larsen, S.C., Liu, H., Yang, X., Wang, C*., et al.* (2025). Interferon-induced PARP14-mediated ADP-ribosylation in p62 bodies requires the ubiquitin-proteasome system. EMBO J.

Point-by-point response to the reviewers' comments and concerns

REVIEWERS' COMMENTS

Reviewer #1 (Remarks to the Author):

In the revised manuscript the authors addressed all my comments. I appreciate their effort to repeat experiments with a commercial column as well as address the comment regarding the isolation windows. Given these additions, I now find the manuscript suitable for publication.

AUTHOR RESPONSE: Thank you very much.
